# Supplementary material for: Trends in inpatient and post-discharge mortality among young infants admitted to Kilifi County Hospital, Kenya: a retrospective cohort study
Source: BMJ Open. 2023 Jan 11;13(1):e067482. doi: 10.1136/bmjopen-2022-067482 (PMC9835934; doi:10.1136/bmjopen-2022-067482)

Supplementary materials

Table of Contents

Table S1**.** List of health workers strikes during study period.	Page 2
Table S2. Proportion of missing data	3
Table S3 Pathogens isolated from blood and CSF cultures of young infants resident in KHDSS during inpatient period.	4
Table S4. Annual admissions and case fatality ratios	5
Table S5**.** Univariate analysis of admission features associated with inpatient deaths.	6,7
Table S6**.** Multivariable regression analysis of factors associated with inpatient mortality (single vs multiple admissions).	8,9
Table S7**.** Estimated causes of post-discharge deaths during readmission at KCH (67 deaths).	10
Table S8. Univariate analysis of admission features associated with post-discharge deaths**.**	11,12
Table S9. Univariate analysis of admission features associated with inpatient deaths among babies born at KCH only.	13,14
Table S10. Multivariable regression analysis of factors associated with inpatient and post-discharge mortality among children born at KCH only.	15
Figure S1. A: Monthly all young infant admissions, B: all admissions (<60 months old), C: all admissions (<60 months old) case fatality and D: 2–59 months old case fatality before and after July 2013.	16

Table S1. **List of health workers strikes during study period.**

**Strike dates**	**Health workers on strike**	**Duration of strike in days**
5 to 13 December 2011	Doctors	9
1 to 15 March 2012	Nurses	15
13 September to 4 October 2012	Doctors	22
3 December 2012 to 13 January 2013	Nurses	42
16 January to 11 February 2013	Nurses	26
10 to 23 December 2013	Doctors & nurses	14
5 to 14 December 2016	Nurses	11
5 December 2016 to 15 March 2017	Doctors	102
5 June to 2 November 2017	Nurses	150

Talbert A, *et al. BMJ Open* 2023; 13:e067482. doi: 10.1136/bmjopen-2022-067482

Table S2. **Proportion of missing data**

**N=4,421**	N missing	% missing
Demographics		
Age in days	0	
Sex (female)	0	
BCG scar	76	1.7
Reported born premature	321	7.3
Reported low birth weight	322	7.3
**Anthropometry**		
Weight (kg)	43	1.0
MUAC (cm)	126	2.9
**Clinical features**		
Axillary temperature	34	0.8
Tachypnea	208	4.7
Tachycardia	43	1.0
Hypoxia (SaO2 <90%)	20	0.5
Lower chest wall indrawing	68	1.5
Wheeze	69	1.6
Stridor	71	1.6
Breathing difficulty	64	1.5
Cyanosis	70	1.6
Capillary refill ≥2 seconds	34	0.8
Temperature gradient	81	1.8
Weak pulse	71	1.6
Lethargy	70	1.6
Impaired consciousness	70	1.6
Bulging fontanel	72	1.6
Stiff neck	71	1.6
Convulsions	64	1.5
Sunken eyes	73	1.7
Reduced skin turgor	71	1.6
Pallor	70	1.6
**Laboratory features**		
HIV antibody positive	756	17
Malaria slide positive	372	8.4
Bacteraemia	2281	52
Haemoglobin	448	10
WBC	449	10
Platelets	448	10
Blood glucose (mmol/L)	1433	32

Table S3 **Pathogens isolated from blood and CSF cultures of young infants resident in KHDSS during inpatient period**.

**Blood culture Isolates**		**CSF culture isolates**	
**Pathogen (N=178)**	No. (%)	**Pathogen (N=24)**	No. (%)
Klebsiella pneumoniae	53 (30)	*Escherichia coli*	6 (25)
Escherichia coli	25 (14)	Group B Streptococcus	6 (25)
Staphylococcus aureus	22 (12)	*Klebsiella pneumoniae*	3 (13)
Group B Streptococcus	19 (11)	*Streptococcus pneumoniae*	3 (13)
Non-typhoidal Salmonella species	9 (5.1)	*Enterobacter cloacae*	3 (13)
Enterobacter cloacae	8 (4.5)	Non-typhoidal Salmonella species	2 (8.2)
Pseudomonas aeruginosa	6 (3.4)	*Acinetobacter lwoffi*	1 (4.2)
Streptococcus pneumoniae	5 (2.8)		
Streptococcus pyogenes	3 (1.7)		
Acinetobacter species	3 (1.7)		
Aeromonas hydrophila	3 (1.7)		
Group A Streptococcus	3 (1.7)		
Serratia marcescens	2 (1.1)		
Acinetobacter calcoaceticus/baumannii	2 (1.1)		
Acinetobacter lwoffi	1 (0.6)		
Aeromonas sobria	1 (0.6)		
Chryseobacterium indologenes	1 (0.6)		
Enterobacter aerogenes	1 (0.6)		
Enterococci species	1 (0.6)		
Haemophilus influenzae	1 (0.6)		
Proteus mirabilis	1 (0.6)		

CSF; cerebrospinal fluid, Out of the 33 Meningitis cases, only the 24 presented had positive CSF culture.

Talbert A, *et al. BMJ Open* 2023; 13:e067482. doi: 10.1136/bmjopen-2022-067482

Table S4. **Annual admissions and case fatality ratios (CFR).**

Admissions/Year	**2009**	**2010**	**2011**	**2012**	**2013**	**2014**	**2015**	**2016**	**2017**	**2018**	**2019**
<60 days	407	455	425	418	319	424	429	472	275	323	474
2 to 59 months	1734	1462	1222	1177	682	1050	1017	787	432	920	918
Proportion of YI admissions	19%	24%	26%	28%	32%	29%	30%	37%	39%	26%	34%
YI inpatient deaths	46	66	72	64	47	59	71	65	51	42	62
YI inpatient CFR	11%	15%	17%	15%	15%	14%	17%	14%	19%	13%	13%
YI post-discharge 1-year deaths	19	24	17	18	25	15	21	19	10	15	25
YI Post-discharge 1- year CFR	5.4%	6.4%	5.1%	5.3%	9.5%	4.3%	6.2%	4.8%	4.7%	5.6%	6.3%

Table S5. **Univariate analysis of admission features associated with inpatient deaths.**

**N=4,421**	**Deaths (N=645) N (%)**	**Crude SHR**	**P-value**	**Scaled Schoenfeld residuals P-value**
Demographics				
Age in days				
0 to 2	511 (19)	3.31 (2.39–4.58)	<0.001	0.14
3 to 7	56 (12)	2.07 (1.38–3.11)	<0.001	
8 to 28	40 (6.8)	1.15 (0.74–1.78)	0.54	
>28	38 (6.0)	Reference		
Sex (female)	268 (14)	0.99 (0.91–1.09)	0.93	0.77
Reported born premature	294 (29)	2.53 (2.32–3.77)	<0.001	0.06
Reported low birth weight	222 (38)	3.25 (2.97–3.56)	<0.001	0.18
Born at KCH				
Yes	382 (14)	Reference		
No	263 (16)	1.13 (0.97–1.32)	0.12	0.61
**Anthropometry**				
Weight (kg)				
<1.5	213 (38)	4.95 (4.13–5.93)	<0.001	0.09
1.5 to <2.5	174 (15)	1.86(1.53–2.26)	<0.001	
≥2.5	229 (8.5)	Reference		
Missing weight	29 (67)	10.7 (7.60–14.9)	<0.001	
MUAC (cm)				
<9.0	333 (25)	3.57 (3.07–4.15)	<0.001	0.08
9 to 10	106 (12)	1.73 (1.45–2.07)	<0.001	
10 to 11	96 (9.3)	1.48 (1.24–1.77)	<0.001	
≥11	75 (7.1)	Reference		
Missing MUAC	35 (28)	3.89 (3.02–5.00)	<0.001	
**Clinical features**				
Axillary temperature				
<36˚C	390 (29)	3.13 (2.82–3.47)	<0.001	0.34
36 to 37.5˚C	159 (9.3)	Reference		
>37.5˚C	84 (6.3)	0.77 (0.67–0.88)	<0.001	
Missing temperature	12 (35)	3.78 (2.65–5.39)	<0.001	
Respiratory rate/min				
Bradypnoea	108 (57)	6.09 (4.98–7.46	<0.001	0.71
Normal	329 (12)	Reference		
Tachypnoea	197 (13)	1.07 (0.90–1.27)	0.45	
Missing	11 (11)	0.91 (0.51–1.65)	0.76	
Heart rate/min				
Bradycardia	74 (47)	4.05 (3.21–5.11)	<0.001	0.50
Normal	403 (14)	Reference		
Tachycardia	163 (12)	0.88 (0.73–1.05)	0.15	
Missing	5 (23)	1.68 (0.72–3.93)	0.23	
Hypoxia (SaO2 <90%)	309 (33)	3.91 (3.58–4.26)	<0.001	0.48
Lower chest wall indrawing	448 (22)	2.86 (2.60–3.15)	<0.001	0.41
Wheeze	0	-		

Stridor	6 (32)	1.48 (0.89–2.47)	0.13	0.23
Breathing difficulty	481 (22)	3.62 (3.26–4.02)	<0.001	0.28
Cyanosis	98 (47)	4.01 (3.55–4.53)	<0.001	0.14
Capillary refill >2 seconds	61 (58)	5.61 (4.41–7.14)	<0.001	0.54
Temperature gradient	98 (38)	2.85 (2.51–3.23)	<0.001	0.36
Weak pulse	108 (69)	5.85 (5.21–6.57)	<0.001	0.71
Lethargy	64 (20)	1.20 (1.04–1.40)	0.02	0.34
Impaired consciousness	140 (56)	5.43 (4.91–6.00)	<0.001	0.42
Bulging fontanel	6 (19)	1.34 (0.90–1.99)	0.15	0.08
Stiff neck	4 (40)	2.08 (1.30–3.32)	0.002	0.17
Convulsions	16 (8.1)	0.75 (0.61–0.93)	0.01	0.53
Sunken eyes	5 (11)	1.04 (0.69–1.56)	0.85	0.52
Reduced skin turgor	21 (22)	1.18 (0.91–1.52)	0.22	0.56
Pallor	72 (33)	2.47 (2.15–2.83)	<0.001	0.63
**Laboratory features**				
Meningitis	8 (24)	8.06 (3.96–16.4)	<0.001	0.13
Anaemia (haemoglobin <110 g/L)	50 (11)	0.67 (0.51–0.90)	0.007	0.51
HIV antibody positive	29 (20)	1.39 (1.13–1.71)	0.002	0.28
Malaria slide positive	0	-		
Bacteraemia	63 (37)	2.92 (2.10–4.06)	<0.001	0.13
Blood glucose (mmol/L)				
<2.6	137 (16)	1.18 (1.04–1.33)	0.009	0.42
2.6 to 7.0	229 (12)	Reference		
>7.0	71 (31)	2.85 (2.47–3.29)	<0.001	
Missing blood glucose	208 (15)	1.18 (1.06–1.32)	0.002	
White blood cells (10^9cells/L)				
<4	14 (26)	2.43 (1.47–4.03)	0.001	
4-20	362 (11)	Reference		0.70
>20	210 (30)	2.97 (2.52–3.50)	<0.001	
Missing	59 (13)	1.19 (0.91–1.57)	0.20	
Platelets (10^9 /L)				
<150	139 (24)	1.89 (1.57–2.27)	<0.001	
≥150	447 (13)	Reference		0.10
Missing	59 (13)	1.01 (0.77–1.32)	0.95	

SHR: sub-distribution hazard ratios; the SHR are from the Fine and Gray's proportional sub-hazards model.

Table S6**. Multivariable regression analysis of factors associated with inpatient mortality (single vs multiple admissions).**

	Multiple admissions (N=4421)		Single admissions (N=4272)	
	Adjusted SHR*	P-value	Adjusted SHR*	P-value
Demographics				
Age in days				
0 to 2	2·12 (1·46–3·06)	<0·001	2·14 (1·49–3·10)	<0·001
3 to 7	3·88 (2·46–6·10)	<0·001	3·92 (2·48–5·16)	<0·001
8 to 28	1·42 (0·90–2·25)	0·13	1·50 (0·95–2·38)	0·08
>28	Reference		Reference	
Sex (female)	0·91 (0·78–1·07)	0·26	0·90 (0·77–1·06)	0·20
**Anthropometry**				
Weight (kg)				
<1.5	2·16 (1·75–2·67)	<0·001	2·26 (1·83–2·79)	<0·001
1.5 to <2.5	1·42 (1·16–1·74)	0·001	1·43 (1·17–1·76)	<0·001
≥2.5	Reference		Reference	
Missing weight	3·85 (2·59–5·71)	<0·001	3·78 (2·56–5·58)	<0·001
**Clinical features**				
Axillary temperature				
<36˚C	1·44 (1·17–1·78)	0·001	1·45 (1·17–1·79)	0·001
36 to 37·5˚C	Reference		Reference	
>37·5˚C	1·09 (0·84–1·41)	0·53	1·07 (0·83–1·39)	0·57
Missing temperature	1·03 (0·38–2·75)	0·96	0·9**2** (0·33–2·51)	0·87
Respiratory rate/min				
Bradypnoea	1·45 (1·09–1·93)	0·01	1·44 (1·08–1·92)	0·01
Normal	Reference		Reference	
Tachypnoea	0·80 (0·67–0·95)	0·01	0·79 (0·66–0·94)	0·009
Missing	1·51 (0·64–3·56)	0·34	1·48 (0·62–3·55)	0·38
Heart rate/min				
Bradycardia	1·40 (1·08–1·82)	0·01	1·42 (1·09–1·85)	0·008
Normal	Reference		Reference	
Tachycardia	1·14 (0·94–1·37)	0·18	1·15 (0·96–1·39)	0·14
Missing	0·41 (0·03–5·13)	0·49	0·45 (0·04–5·14)	0·52
Hypoxia (SaO2 <90%)	1·62 (1·37–1·92)	<0·001	1·60 (1·36–1·90)	<0·001
Capillary refill >2 seconds	1·34 (0·97–1·86)	0·08	1·31 (0·94–1·83)	0·11
Lower chest wall indrawing	1·41 (1·14–1·75)	0·002	1·42 (1·14–1·77)	0·002
Stridor	1·93 (0·92–4·03)	0·08	1·85 (0·89–3·85)	0·10
Breathing difficulty	1·45 (1·15–1·82)	0·001	1·44 (1·15–1·81)	0·002
Weak pulse	1·61 (1·19–2·17)	0·002	1·61 (1·18–2·18)	0·002
Bulging fontanel	2·45 (0·91–6·65)	0·08	2·41 (0·90–6·55)	0·08
Impaired consciousness	2·21 (1·72–2·84)	<0·001	2·17 (1·68–2·78)	<0·001
Pallor	1·30 (0·98–1·71)	0·07	1·28 (0·97–1·69)	0·08
**Laboratory features**				
Meningitis	5·45 (2·50–11·8)	<0·001	5·18 (2·39–11·2)	<0·001
HIV antibody positive	1·15 (0·81–1·63)	0·43	1·14 (0·80–1·62)	0·47

Talbert A, *et al. BMJ Open* 2023; 13:e067482. doi: 10.1136/bmjopen-2022-067482

Bacteraemia	2·21 (1·51–3·22)	<0·001	2·20 (1·51–3·20)	<0·001
White blood cells (10^9 cells/L)				
<4	2·17 (1·30–3·62)	0·003	2·11 (1·27–3·50)	0·003
4-20	Reference		Reference	
>20	1·71 (1·43–2·04)	<0·001	1·70 (1·43–2·03)	<0·001
unavailable	1·09 (0.82–1·44)	0·57	1·06 (0.79–1·41)	0·71
Model performance				
Bootstrapped AUC (95% CI)	0·88 (0·86–0·89)		0·88 (0·86–0·89)	

SHR; sub-distribution hazard ratios; *the SHR are from the Fine and Gray's proportional sub-hazards model, AUC; area under receiver operating characteristics. Meningitis: positive CSF culture, or positive CSF microscopy, or positive CSF antigen test, or elevated CSF WBC count (≥0.02x10^9/L in young infants aged 0-28 days OR, ≥0.01x10^9/L in young infants aged 29-59 days) PLUS a positive blood culture for a known pathogen

Table S7. **Estimated causes of post-discharge deaths during readmission at KCH (67 deaths**).

**Index admission diagnosis (N=67)**	**No. (%)**	**Causes of post-discharge deaths (N=67)**	**No. (%)**
Neonatal sepsis	15 (22)	Neonatal sepsis	16 (24)
Preterm complications	15 (22)	Preterm complications	15 (22)
Heart disease-Congenital	9 (13)	Heart disease-Congenital	10 (15)
Neonatal jaundice	5 (7.5)	Neonatal jaundice	5 (7.4)
Meningitis	4 (6.0)	Meningitis	5 (7.4)
Birth asphyxia	4 (6.0)	Birth asphyxia	5 (7.4)
Lower respiratory tract infection	4 (6.0)	Lower respiratory tract infection	4 (6.0)
Encephalopathy - unknown	0	Encephalopathy - unknown	1 (1.5)
Hydrocephalus	1 (1.5)	Hydrocephalus	1 (1.5)
Malnutrition	1 (1.5)	None specified	5 (7.4)
None specified	9 (13)		

Index admission diagnosis and causes of death were assigned by treating clinician.

Talbert A, *et al. BMJ Open* 2023; 13:e067482. doi: 10.1136/bmjopen-2022-067482

Table S8. **Univariate analysis of admission features associated with post-discharge deaths.**

**N=3625**	**Deaths (N=208)**	**Crude HR**	**P-value**	**Scaled Schoenfeld residuals P-value**
Demographics				
Age in days				
0 to 2	124 (5.6)	0.98 (0.67–1.44)	0.92	0.10
3 to 7	15 (3.7)	0.63 (0.34–1.16)	0.14	
8 to 28	36 (6.6)	1.17 (0.73–1.87)	0.52	
>28	33 (5.5)	Reference		
Sex (female)	89 (5.5)	0.98 (0.74–1.28)	0.86	0.79
Reported born premature	58 (8.0)	1.79 (1.32–2.44)	<0.001	0.07
Reported low birth weight	33 (9.3)	1.99 (1.37–2.90)	<0.001	0.13
Born at KCH				
Yes	102 (4.3)	Reference		
No	106 (7.6)	1.75 (1.34–2.30)	<0.001	0.12
Length of hospitalization (days)-log transformed	-	1.96 (1.68–2.27)	<0.001	0.38
Discharged over weekend				
No	173 (5.7)	Reference		
Yes	35 (4.8)	0.85 (0.59–1.23)	0.39	0.16
Type of discharge				
Normal discharge	180 (4.9)	Reference		0.75
Absconded	5 (12)	2.60 (1.07–6.33)	0.04	
Transferred/referred	23 (44)	11.8 (7.64–18.2)	<0.001	
**Anthropometry**				
Weight (kg)				
<1.5	30 (8.5)	2.49 (1.65–3.77)	<0.001	0.17
1.5 to <2.5	87 (9.2)	2.64 (1.97–3.54)	<0.001	
≥2.5	91 (3.7)	Reference		
Missing weight	0	-		
MUAC (cm)				
<9.0	88 (8.8)	4.05 (2.56–6.41)	<0.001	0.17
9 to 10	44 (5.8)	2.56 (1.55–4.24)	<0.001	
10 to 11	42 (4.5)	1.92 (1.15–3.18)	0.01	
≥11	23 (2.4)	Reference		
Missing MUAC	11 (12)	5.83 (2.84–12.0)	<0.001	
**Clinical features**				
Axillary temperature				
<36˚C	78 (8.1)	1.45 (1.07–1.96)	0.02	0.80
36 to 37.5˚C	88 (5.7)	Reference		
>37.5˚C	41 (3.3)	0.57 (0.40–0.83)	0.003	
Missing temperature	1 (5.0)	0.99 (0.14–7.12)	0.99	
Respiratory rate/min				
Bradypnoea	7 (8.8)	1.92 (0.90–4.13)	0.09	0.30
Normal	108 (4.7)	Reference		
Tachypnoea	87 (6.8)	1.44 (1.09–1.92)	0.01	
Missing	6 (7.2)	1.65 (0.72–3.76)	0.23	
Heart rate/min				

Bradycardia	9 (11)	2.10 (1.07–4.13)	0.03	0.73
Normal	137 (5.5)	Reference		
Tachycardia	62 (5.3)	0.97 (0.72–1.31)	0.85	
Missing	0	-		
Hypoxia (SaO2 <90%)	51 (8.2)	1.68 (1.23–2.31)	0.001	0.54
Lower chest wall indrawing	108 (6.8)	1.54 (1.17–2.02)	0.002	0.18
Wheeze	2 (4.4)	0.77 (0.19–3.10)	0.71	0.20
Stridor	0	-		
Breathing difficulty	109 (6.5)	1.40 (1.07–1.85)	0.02	0.26
Cyanosis	7 (6.3)	1.14 (0.54–2.43)	0.73	0.17
Capillary refill ≥2 seconds	4 (9.4)	1.81 (0.67–4.87)	0.24	0.40
Temperature gradient	12 (7.5)	1.44 (0.80–2.57)	0.23	0.22
Weak pulse	7 (15)	3.10 (1.46–6.59)	0.003	0.38
Lethargy	14 (5.8)	1.06 (0.63–1.80)	0.82	0.19
Impaired consciousness	6 (5.5)	0.98 (0.44–2.21)	0.96	0.50
Bulging fontanel	4 (15)	3.04 (1.13–8.18)	0.03	0.06
Stiff neck	1 (17)	2.84 (0.40–20.2)	0.30	0.15
Convulsions	9 (5.0)	0.88 (0.46–1.75)	0.75	0.31
Sunken eyes	6 (15)	3.31 (1.47–7.45)	0.004	0.20
Reduced skin turgor	8 (11)	2.19 (1.08–4.43)	0.03	0.20
Pallor	15 (10)	2.09 (1.23–3.53)	0.006	0.23
**Laboratory features**				
Meningitis	4 (16)	3.98 (1.45–10.9)	0.007	0.13
Anaemia (haemoglobin <110 g/L)	26 (6.2)	1.19 (0.79–1.80)	0.41	0.68
HIV antibody positive	7 (6.2)	1.17 (0.55–2.49)	0.69	0.76
Malaria slide positive	0	-		
Bacteraemia	10 (9.4)	1.02 (0.50–2.06)	0.96	0.28
Blood glucose (mmol/L)				
<2.6	51 (6.9)	1.31 (0.92–1.85)	013	0.25
2.6 to 7.0	86 (5.3)	Reference		
>7.0	10 (6.3)	1.21 (0.63–2.32)	0.57	
Missing blood glucose	61 (5.0)	0.95 (0.68–1.32)	0.75	
White blood cells (10^9/L)				
<4	1 (2.5)	0.45 (0.06–3.20)	0.42	0.79
4-20	157 (5.5)	Reference		
>20	29 (6.1)	1.10 (0.74–1.64)	0.63	
Missing	21 (5.4)	0.99 (0.63–1.56)	0.97	
Platelets (10^9 /L)				
<150	38 (8.6)	1.69 (1.18–2.41)	0.004	0.70
≥150	149 (5.1)	Reference		
Missing	21 (5.4)	1.08 (0.68–1.70)	0.75	
HR: hazard ratios; the HR are from the Cox proportional hazards model.				

12

Table S9. **Univariate regression analysis of factors associated with inpatient and post-discharge mortality among children born at KCH only.**

**N=1,358**	**Deaths (N=174) N (%)**	**Crude SHR**	**P-value**	**Scaled Schoenfeld residuals P-value**
Demographics				
Age in days				
0 to 2	151 (15)	2.48 (1.19–5.16)	0.02	0.26
3 to 7	8 (7.0)	1.22 (0.45–3.30)	0.69	
8 to 28	8 (6.8)	1.21 (0.46–3.23)	0.70	
>28	7 (5.5)	Reference		
Sex (female)	84 (14)	1.11 (0.83–1.48)	0.50	0.31
Born premature	101 (21)	2.65 (1.97–3.56)	<0.001	0.10
Born low birth weight	107 (21)	2.74 (2.03–3.71)	<0.001	0.44
**Anthropometry**				
Weight z score at birth				
<-2.0	32 (17)	1.49 (1.02–2.17)	0.04	0.12
≥-2.0	142 (12)	Reference		
MUAC (cm)				
<9.0	97 (23)	3.14 (2.05–4.83)	<0.001	0.17
9 to 10	26 (9.9)	1.26 (0.74–2.17)	0.40	
10 to 11	25 (7.3)	0.91 (0.53–1.57)	0.73	
≥11	26 (7.9)	Reference		
Missing MUAC	0	-		
**Clinical features**				
Axillary temperature				
<36˚C	113 (26)	3.02 (2.13–4.27)	<0.001	0.30
36 to 37.5˚C	41 (9.2)	Reference		
>37.5˚C	18 (3.9)	0.42 (0.24–0.72)	0.002	
Missing temperature	2 (50)	6.90 (1.84–25.8)	0.004	
Respiratory rate/min				
Bradypnoea	32 (52)	6.44 (4.39–9.45)	<0.001	0.17
Normal	91 (10)	Reference		
Tachypnoea	50 (12)	1.21 (0.86–1.69)	0.28	
Missing	1 (5.3)	0.49 (0.07–3.37)	0.47	
Heart rate/min				
Bradycardia	21 (43)	4.76 (3.06–7.43)	<0.001	0.81
Normal	98 (10)	Reference		
Tachycardia	55 (15)	1.48 (1.07–2.05)	0.02	

Missing	0	-		
Hypoxia (SaO2 <90%)	79 (29)	3.62 (2.71–4.83)	<0.001	0.81
Lower chest wall indrawing	125 (21)	3.57 (2.57–4.95)	<0.001	0.47
Wheeze	0	-		
Stridor	5 (63)	6.96 (3.09–15.7)	<0.001	0.60
Breathing difficulty	481 (22)	5.40 (3.69–7.88)	<0.001	0.18
Cyanosis	29 (48)	5.15 (3.54–7.50)	<0.001	0.18
Capillary refill >2 seconds	14 (64)	7.22 (4.43–11.7)	<0.001	0.58
Temperature gradient	26 (30)	2.69 (1.81–4.01)	<0.001	0.49
Weak pulse	25 (58)	6.52 (4.44–9.56)	<0.001	0.77
Lethargy	17 (16)	1.29 (0.79–2.10)	0.31	0.56
Impaired consciousness	48 (52)	6.75 (4.93–9.24)	<0.001	0.13
Bulging fontanel	1 (25)	2.03 (0.31–13.2)	0.46	0.59
Stiff neck	1 (5.3)	-		0.31
Convulsions	1 (2.9)	0.21 (0.03–1.48)	0.12	0.21
Sunken eyes	1 (17)	1.35 (0.19–9.77)	0.77	0.57
Reduced skin turgor	1 (5.9)	0.44 (0.06–3.19)	0.42	0.56
Pallor	21 (42)	4.00 (2.61–6.12)	<0.001	0.59
**Laboratory features**				
Meningitis	2 (29)	9.95 (2.37–41.8)	0.002	0.12
Haemoglobin <110 g/L	11 (13)	0.92 (0.51–1.66)	0.78	0.25
HIV antibody positive	8 (21)	1.68 (0.84–3.33)	0.14	0.14
Malaria slide positive	0	-		
Bacteraemia	14 (33)	3.00 (1.48–5.95)	0.002	0.20
Blood glucose (mmol/L)				
<2.6	33 (12)	1.02 (0.67–1.56)	0.91	0.95
2.6 to 7.0	58 (12)	Reference		
>7.0	11 (21)	1.88 (1.00–3.54)	0.05	
Missing blood glucose	72 (13)	1.11 (0.79–1.56)	0.53	
White blood cells (10^9 /L)				
<4	3 (33)	3.52 (1.25–9.91)	0.02	
4-20	100 (10)	Reference		0.08
>20	59 (28)	3.10 (2.27–4.24)	<0.001	
Missing	12 (8.2)	0.82 (0.45–1.48)	0.50	
Platelets (10^9 /L)				
<150	35 (20)	1.69 (1.18–2.44)	0.005	
≥150	127 (12)	Reference		0.50
Missing	12 (8.2)	0.66 (0.37–1.19)	0.17	

SHR; sub-distribution hazard ratios; the SHR are from the Fine and Gray's proportional sub-hazards model.

Table S10. **Multivariable regression analysis of factors associated with inpatient and post-discharge mortality among children born at KCH only.**

	Inpatient analysis		Post-discharge analysis	
	Adjusted SHR*	P-value	Adjusted HR	P-value
**Demographics**				
Age in days				
0 to 2	3.03 (1.33–6.94)	0.009	¶	
3 to 7	4.08 (1.48–11.3)	0.007	¶	
8 to 28	2.57 (0.90–7.29)	0.08	¶	
>28	Reference			
**Anthropometry**				
Low birth weight	1.55 (1.10–2.20)	0.01	2.76 (1.30–5.82)	0.008
**Clinical features**				
Axillary temperature				
<36˚C	1.49 (0.97–2.28)	0.07	1.44 (0.71–2.95)	0.31
36 to 37.5˚C	Reference		Reference	
>37.5˚C	0.67 (0.39–1.13)	0.13	0.22 (0.06–0.78)	0.02
Missing temperature	3.37 (0.85–13.4)	0.09	0.48 (0.09–2.64)	0.40
Respiratory rate/min				
Bradypnoea	2.22 (1.36–3.63)	0.001	¶	
Normal	Reference			
Tachypnoea	0.78 (0.55–1.11)	0.17	¶	
Missing	-			
Heart rate/min				
Bradycardia	1.88 (1.14–3.12)	0.01	3.56 (1.14–11.2)	0.03
Normal	Reference		Reference	
Tachycardia	1.57 (1.11–2.21)	0.01	1.54 (0.77–3.07)	0.22
Missing	0.25 (0.02–3.00)	0.28	-	
Hypoxia (SaO2 <90%)	1.60 (1.14–2.24)	0.006	¶	
Lower chest wall indrawing	1.42 (0.91–2.22)	0.12	¶	
Stridor	3.74 (1.87–7.49)	<0.001	¶	
Breathing difficulty	2.13 (1.25–3.64)	0.005	¶	
Capillary refill >2 seconds	1.94 (1.06–3.56)	0.03	¶	
Weak pulse	2.15 (1.27–3.65)	0.004	1.60 (0.24–10.5)	0.63
Pallor	2.36 (1.46–3.83)	<0.001	¶	
**Laboratory features**				
Bacteraemia	2.50 (1.20–5.22)	0.02	0.21 (0.03–1.81)	0.16
Model performance				
AUC (95% CI)	0.85 (0.82–0.88)		0.79 (0.72–0.85)	

SHR; sub-distribution hazard ratios; *the SHR are from the Fine and Gray's proportional subhazards model, HR-Hazard ratio from the Proportional Cox regression model, ¶; variables not selected for inclusion in the multivariable model, AUC; area under receiver operating characteristics.

Figure S1. **A: Monthly all young infant admissions, B: all admissions (<60 months old), C: all admissions (<60 months old) case fatality and D: 2–59 months old case fatality before and after July 2013.**

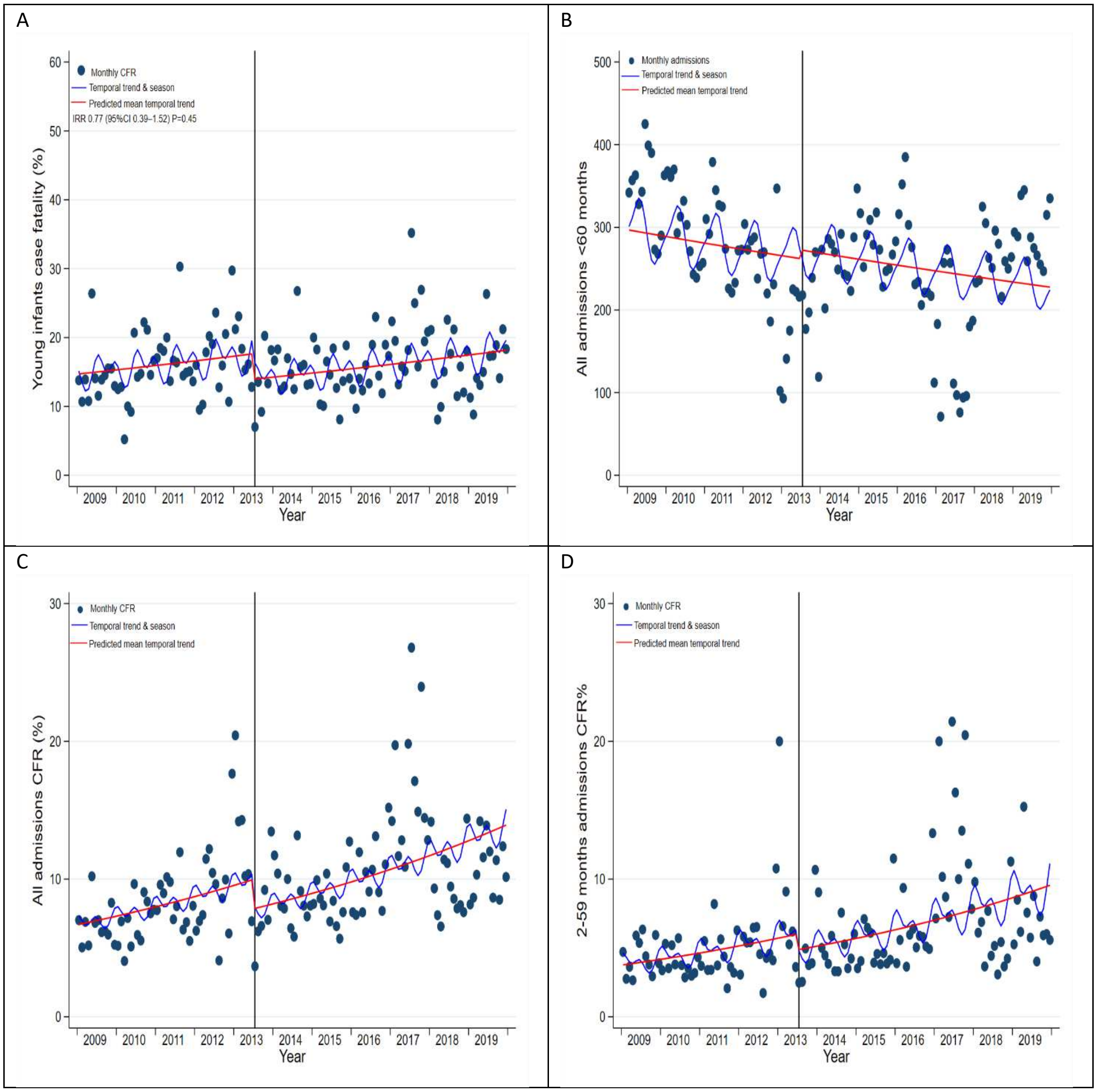

Supplement: Supplementary data [file bmjopen-2022-067482supp001.pdf]
