# Supplementary material for: Trends in inpatient and post-discharge mortality among young infants admitted to Kilifi County Hospital, Kenya: a retrospective cohort study
Source: BMJ Open. 2023 Jan 11;13(1):e067482. doi: 10.1136/bmjopen-2022-067482 (PMC9835934; doi:10.1136/bmjopen-2022-067482)

BMJ Open

BMJ Open is committed to open peer review. As part of this commitment we make the peer review history of every article we publish publicly available.

When an article is published we post the peer reviewers' comments and the authors' responses online. We also post the versions of the paper that were used during peer review. These are the versions that the peer review comments apply to.

The versions of the paper that follow are the versions that were submitted during the peer review process. They are not the versions of record or the final published versions. They should not be cited or distributed as the published version of this manuscript.

BMJ Open is an open access journal and the full, final, typeset and author-corrected version of record of the manuscript is available on our site with no access controls, subscription charges or pay-per-view fees (http://bmjopen.bmj.com).

If you have any questions on BMJ Open's open peer review process please email info.bmjopen@bmj.com

BMJ Open

# Inpatient and post-discharge mortality among young infants in rural Kenya.

| | |
|---|---|
| Journal: | *BMJ Open* |
| Manuscript ID | bmjopen-2022-067482 |
| Article Type: | Original research |
| Date Submitted by the Author: | 18-Aug-2022 |
| Complete List of Authors: | Talbert, Alison; KEMRI-Wellcome Trust Research Programme<br>Ngari, Moses; KEMRI-Wellcome Trust Research Programme<br>Obiero, Christina; KEMRI-Wellcome Trust Research Programme; University of Amsterdam, Department of Global Health<br>Nyaguara, A.; KEMRI-Wellcome Trust Research Programme<br>Mwangome, MK; KEMRI-Wellcome Trust Research Programme<br>Mturi, Neema; KEMRI-Wellcome Trust Research Programme<br>Ouma, Nelson; KEMRI-Wellcome Trust Research Programme<br>Otiende, M.; KEMRI-Wellcome Trust Research Programme<br>Berkley, James ; KEMRI-Wellcome Trust Research Programme; University of Oxford,  Centre for Tropical Medicine & Global Health |
| Keywords: | Epidemiology < TROPICAL MEDICINE, PAEDIATRICS, NEONATOLOGY |
| | |

SCHOLARONE™
Manuscripts

BMJ

*I, the Submitting Author has the right to grant and does grant on behalf of all authors of the Work (as defined in the below author licence), an exclusive licence and/or a non-exclusive licence for contributions from authors who are: i) UK Crown employees; ii) where BMJ has agreed a CC-BY licence shall apply, and/or iii) in accordance with the terms applicable for US Federal Government officers or employees acting as part of their official duties; on a worldwide, perpetual, irrevocable, royalty-free basis to BMJ Publishing Group Ltd ("BMJ") its licensees and where the relevant Journal is co-owned by BMJ to the co-owners of the Journal, to publish the Work in this journal and any other BMJ products and to exploit all rights, as set out in our licence.*

*The Submitting Author accepts and understands that any supply made under these terms is made by BMJ to the Submitting Author unless you are acting as an employee on behalf of your employer or a postgraduate student of an affiliated institution which is paying any applicable article publishing charge ("APC") for Open Access articles. Where the Submitting Author wishes to make the Work available on an Open Access basis (and intends to pay the relevant APC), the terms of reuse of such Open Access shall be governed by a Creative Commons licence – details of these licences and which Creative Commons licence will apply to this Work are set out in our licence referred to above.*

*Other than as permitted in any relevant BMJ Author's Self Archiving Policies, I confirm this Work has not been accepted for publication elsewhere, is not being considered for publication elsewhere and does not duplicate material already published. I confirm all authors consent to publication of this Work and authorise the granting of this licence.*

For peer review only

# Inpatient and post-discharge mortality among young infants in rural Kenya.

Alison Talbert MRCP [1], Moses Ngari PhD[1,2], Christina Obiero MPH[1,3], Amek Nyaguara PhD[1], Martha Mwangome PhD[1,2], Neema Mturi MRCP[1], Nelson Ouma BSc[1], Mark Otiende MSc[1], and James A Berkley FRCPCH[1,2,4]

Institutions of affiliation

1. KEMRI/Wellcome Trust Research Programme, Centre for Geographic Medicine Research -Coast, PO Box 230 - 80108, Kilifi, Kenya.
2. The Childhood Acute Illness & Nutrition Network (CHAIN), Nairobi, Kenya.
3. Department of Global Health, University of Amsterdam, Amsterdam, The Netherlands
4. Center for Tropical Medicine & Global Health, University of Oxford, Oxford, UK.

Corresponding author: Alison Talbert Email: ATalbert@kemri-wellcome.org

P.O Box 230,Kilifi, 80108 Kenya Phone: +254 729 218993

Word count 3810



For peer review only - http://bmjopen.bmj.com/site/about/guidelines.xhtml

# Abstract

**Objectives:** to describe admission trends and measure inpatient and post-discharge mortality and its associated exposures, among young infants (YI) admitted to a county hospital in Kenya

**Design:** retrospective cohort study

**Setting:** secondary level hospital

**Participants:** YI aged less than 60 days admitted to hospital January 2009 to December 2019: 12,271 admissions in 11,877 individuals. YI who were resident within a health and demographic surveillance system (KHDSS) ; were followed up for 1 year after discharge.

**Primary and secondary outcome measures:** Inpatient and 1 year post-discharge mortality

**Results:** Of 12,271 YI admissions, 4,421 (36%) were KHDSS-resident. Neonatal sepsis, preterm complications and birth asphyxia accounted for 83% of admissions. The proportion of YI among under-fives admissions increased from 19% in 2009 to 34% in 2019 ($P_{trend}$ =0·02). Inpatient case fatality was 16%, with 66% of deaths occurring within 48 hours of admission. The introduction of free maternity care in 2013 was not associated with a change in admissions or inpatient mortality among YI. During 1-year post-discharge, 208/3625 (5·7%) YI died, 64·3 (95%CI 56·2–73·7) per 1,000 infant-years; 49% of post-discharge deaths occurred within one month of discharge, and 49% of post-discharge deaths occurred at home. Both inpatient and post-discharge deaths were associated with low weight. Inpatient mortality was associated with clinical signs of disease severity, while post-discharge mortality was associated with length of hospitalization, leaving against advice and referral to a specialized hospital.




For peer review only - http://bmjopen.bmj.com/site/about/guidelines.xhtml

**Conclusions:** Young infants accounted for an increasing proportion of paediatric admissions and inpatient deaths. The post-discharge mortality rate of YI is more than twice that of children aged 2-59 months. The proportion of deaths occurring post-discharge is lower than among older children, but similarly, almost half of post-discharge deaths occur in the first month, and half occurred at home.

**Key words**

Young infant; mortality; inpatient; post-discharge; Africa; Kenya

290 words



For peer review only - http://bmjopen.bmj.com/site/about/guidelines.xhtml

For peer review only

## Article summary

## Strengths and limitations of this study

- Large sample size with systematic collection of data
- Linkage to a well-established demographic surveillance system, with few losses to follow up.
- Lack of accurate gestational age estimation and unknown birthweight of most participants.
- Data are from a single hospital



For peer review only - http://bmjopen.bmj.com/site/about/guidelines.xhtml

# Background

The United Nations Sustainable Development Goal 3 aims to ensure healthy living and promote wellbeing for all ages, with all countries aiming to reduce neonatal and under-five mortality to below 12 and 25 per 1,000 live births by 2030 respectively. In sub-Saharan Africa, child mortality has declined by ~58% in the last 30 years. However, the estimated neonatal and under-five mortality rates in sub-Saharan Africa remained high in 2019 (27 and 76 per 1,000 live births respectively) with a similar neonatal mortality rate of 27 per 1,000 live births in Kenya.(1) Combined neonatal and post-neonatal infant mortality accounts for over three quarters of all under-five deaths in Kenyan children.(2)

Young infants aged <60 days old (YI) comprise around half of hospital admissions in sub-Saharan Africa and continue to face high risk of in-hospital mortality and long-term neuro-disability.(3-6) Post-discharge mortality is emerging as a major problem in children in low- and middle-income countries (LMICs),(7) however, there are limited data among YI. A systematic review of paediatric post-discharge mortality in developing countries included 24 studies published up to July 2017 with 19 from Africa.(8) Four studies included YI. Although young age was reported as a risk factor of mortality, no studies specifically identified deaths among infants aged <60 days. We have previously demonstrated excess post-discharge mortality among all hospitalised children, suggesting that hospitalisation itself selects vulnerable children with a sustained increased risk of dying over the longer term.(7, 9)



For peer review only - http://bmjopen.bmj.com/site/about/guidelines.xhtml

89 Better understanding of YI deaths occurring during hospitalisation and after discharge from hospital is vital for development and use of targeted interventions aimed at improving survival.

91 This analysis aimed to describe admission trends and measure inpatient and post-discharge mortality and its associated exposures, including the introduction of free maternity care, among YI admitted to Kilifi County Hospital (KCH), Kenya and followed up through the Kilifi Health and Demographic Surveillance System (KHDSS).

For peer review only



For peer review only - http://bmjopen.bmj.com/site/about/guidelines.xhtml

# Methods

Study participants and design

KCH is a secondary-level referral hospital situated in Kilifi county along the Kenyan coast. It serves a rural and peri-urban population. It has a maternity unit. with approximately 6,000 deliveries per year, a general paediatric ward with a newborn unit for babies aged less than 1 month, and a paediatric High Dependency Unit (HDU) that also admits YIs. The year 2009 was selected as a starting point, because a previous analysis of mortality among YI covered admissions from 1990 to 2008(10). Free maternity care was introduced by the Kenyan government on 1st June 2013 and led to a marked increase in health facility births.(11)

The KHDSS, established in 2002, covers a population of 279,158 within an area of 900km$^2$ centred on KCH.(12) Census rounds visit each household every four months to ascertain vital status and migration in and out of the hospital catchment area.

We conducted a retrospective cohort study of YIs resident within the KHDSS who were admitted to KCH between January 1st, 2009 and December 31st, 2019. Children discharged alive and followed up in KHDSS census rounds until March 2021 were eligible for analyses of post-discharge mortality. During the study period, there were 9 health workers' strikes with the last nurses' strike lasting for 150 days (5 June to 2 November 2017).(13) **Supplementary Table S1.** For comparison, we also examined admissions aged 60 days to 59 months during the same period.



For peer review only - http://bmjopen.bmj.com/site/about/guidelines.xhtml

## Procedures

At admission, standardised medical history, and clinical examination, including anthropometric measurements were obtained by trained clinical staff. Blood samples were systematically taken for complete blood count, slide for malaria microscopy, and clinical chemistry, Human Immunodeficiency Virus (HIV) antibody test and blood culture at hospital admission, as described previously.(14) A lumbar puncture for cerebrospinal fluid (CSF) analysis was done at admission in infants in whom sepsis was suspected and deferred in those seriously ill or with other contraindications. Clinical and laboratory data were recorded in real time on a ward surveillance database linked to the KHDSS database. Empiric antibiotics were initiated according to national guidelines(15) with ampicillin/benzylpenicillin plus gentamicin as first-line intravenous therapy. Second-line and subsequent antimicrobial therapy was guided by blood culture results and clinical progress. Mechanical ventilation was not available at KCH.

## Statistical methods

### *Study variables*

Outcomes of interest were death in hospital and during 1 year after discharge. Exposures of interest were demographic, nutritional, clinical features, and haematological, biochemical, and microbiological findings at the time of admission. De-identified study data were deposited in the Harvard Dataverse depository.(16)



For peer review only - http://bmjopen.bmj.com/site/about/guidelines.xhtml

Weight at admission and mid-upper arm circumference (MUAC) were categorised as shown on **Table 1**. Because approximately 40% of the YI were underweight (<2·5kg), and 60% were aged ≤2 days at admission, admission weights rather than anthropometric Z scores using WHO standards were reported. Furthermore, most YI who were born at home or in other hospitals and referred to KCH were missing gestational age estimates and birth weight to be able to estimate gestational age at birth using the INTERGROWTH 21st Newborn Size Standards (INSS).

Prematurity was defined as gestation age <37 weeks and LBW as birth weight <2500 grams for YIs born at KCH. Admission blood glucose was categorized into <2·6, 2·6 to 7·0 and ≥7·0 mmol/l representing low, normal and high levels respectively.(15) Missing data were not assumed to be missing at random. We, therefore, created categorical variables and added a missing category which was included in the regression analysis.

Demographic, anthropometric, and clinical data are presented as frequencies and proportions for categorical variables and means (standard deviation (sd)) or median (interquartile range (IQR)) for continuous variables depending on the underlying distribution. Proportions of missing data for each variable are shown on **Supplementary Table S2**.

Monthly admissions and case fatality were plotted against time (month of admission) to visually inspect the trend from 2009 to 2019 and the predicted trend line superimposed on the curves. We used the Augmented Dickey Fuller test (ADF test) to test if the time series were stationary (no trend or seasonal effects). We also presented annual absolute admissions, proportion of YI among all admissions <60 months and case fatality. Monthly admissions and case fatality were



For peer review only - http://bmjopen.bmj.com/site/about/guidelines.xhtml

tested for annual linear trend using an extension of the Wilcoxon rank-sum test of trend across ordered groups.(17)

We used interrupted time series analysis to estimate the level and trend changes before and after introduction of free maternity care (1st June 2013). We created a time variable coded sequentially from 1 to 132 representing the months from January 2009 to December 2019 and free maternity care was coded as 0 before June 2013 and 1 from June 2013. We defined seasonal effect variable using month of the year modelled on harmonic terms using the fourier code in STATA. To measure the effect of free maternity care, we used the negative binomial regression model because of presence of overdispersion in the trends and reported regression coefficients transformed into incidence rate ratios (IRR). All the negative binomial regression models included the dependent variable of interest e.g. the monthly number of admissions, and the following independent variables: the time variable, the binary pre- and post- free maternity care variable and the seasonal effect variable.

Since YIs could be admitted more than once whilst <60 days old, we included multiple admissions using unique IDs and adjusted for clustering by individual with robust standard errors. To identify exposures associated with inpatient death, we treated being discharged alive as a competing event and fitted the proportional sub-distribution hazard model using the Fine-Gray competing risk model.(18) The measure of effect reported from the model was the sub-distribution hazard ratios (SHR) and their respective 95% confidence intervals (CI). To build the multivariable regression model, a backward stepwise approach was used where all the independent variables assessed in the univariate models were included in the model and only those with a P-value <0·1 retained in the final multivariable model.

For peer review only



For peer review only - http://bmjopen.bmj.com/site/about/guidelines.xhtml

For the post-discharge analysis, only data from those YI discharged alive and resident within the KHDSS were analysed. Time at risk was defined from date of discharge to 365 days later or censure at date of death or outmigration from the KHDSS. We performed a 'multiple discharges' analysis where YI with multiple admissions had their follow-up time reset at each successive discharge date. Exposures associated with post-discharge assessed using a Cox proportional hazards regression model with robust standard errors accounting for YI with multiple discharges. The proportional hazards assumption was assessed using the scaled Schoenfeld residuals test (**Supplementary Tables S3 and S4**). All exposures assessed in the univariate models were considered for inclusion in the multivariable Cox proportional hazards regression model using a backward stepwise approach. Both the inpatient and post-discharge multivariable regression models' discrimination performance were assessed using bootstrapped area under receiver operating characteristic curves (AUC) replicated 1000 times.

As sensitivity analysis, we assessed the YI born at KCH and enrolled to the Kilifi Perinatal and Maternal Research Project (KIPMAT), which had collected comprehensive birth data including birth weight and gestational age (weeks).(19) We estimated their birthweight Z scores using the INTERGROWTH Newborn Size Standards (INSS) and ran the regression models replacing admission weight with birthweight Z score.(20)

Statistical significance was evaluated using 95% CI and a two-tailed *P*-value <0·05. Statistical analyses were conducted using STATA Version 17.0 (College Station, TX, USA).

*Study size*



For peer review only - http://bmjopen.bmj.com/site/about/guidelines.xhtml

With 3,625 YIs discharged alive and included in the post-discharge analysis, a post-discharge mortality of 5·7% and a two-sided alpha of 0·05, the study had greater than 80% power, to detect hazard ratio of ≥2·0 of death between YIs with admission weight <1·5 Kg compared to those with weight ≥2·5 Kg.

Ethical considerations

Written consent was provided by the caregivers of all the surveillance study participants. Ethical approval to conduct this analysis was granted by the Kenya Medical Research Institute (KEMRI) National Ethics Review Committee (SCC 2778).

Patient and public involvement

There was no patient and public involvement in the planning or execution of this retrospective cohort study.

For peer review only



For peer review only - http://bmjopen.bmj.com/site/about/guidelines.xhtml

## Results

### Baseline characteristics

During the study period, there were 42,742 paediatric admissions to KCH, of which 12,271 (29%) admission events among 11,877 individuals were aged <60 days. Of the 12,271 YI admission events, 4,421 (36%) were resident in the KHDSS and included in the analysis (**Figure 1**). This comprised 4,272 individual YI: 4,131 with one admission, 133 two admissions and 8 three admissions within the first 60 days of life.

### KHDSS-resident admissions

Among the 4,421 YI admission events among KHDSS residents, 2,731 (62%) were ≤2 days old and 1,900 (43%) were female. Reported prematurity and low birth weight were 1,019 (23%) and 581 (13%) respectively. Low weight (<2·5kg) was observed in 1694 YIs (38%) while 1342 (30%) had MUAC <9·0cm. Common presenting clinical signs were lower chest wall indrawing (46%) and breathing difficulty (49%). Thirty percent had fever, 31% had hypothermia and 30% tachycardia. Nine hundred and thirty-two YI (21%) had hypoxia ($SaO_2$ <90%) at admission and 250 (5·7%) had impaired consciousness. Presenting signs at admission for all the YI stratified by KHDSS residence are shown on **Table 1**. Malaria was very rare (n=4, 0·09%) whilst 142 (3·2%) and 170 (3·9%) YI were HIV antibody positive and had bacteraemia respectively. **Supplementary Table S5** lists the bacterial isolates that were presumed pathogens, led by *Klebsiella pneumoniae, Escherichia coli, Staphylococcus aureus* and Group B Streptococcus.



For peer review only - http://bmjopen.bmj.com/site/about/guidelines.xhtml

**Table 1. Study participants characteristics at admission.**

| | All young infant admissions (N=12,271) [a] | Young infant admissions KHDSS residents (N=4,421) | Young infant admissions non-KHDSS residents (N=7,850) | p-value |
|---|---|---|---|---|
| **Demographics** | | | | |
| Age in days | | | | |
| 0 to 2 | 7856 (64) | 2731 (62) | 5125 (65) | <0·001 |
| 3 to 7 | 1384 (11) | 468 (11) | 916 (12) | |
| 8 to 28 | 1506 (12) | 587 (13) | 919 (12) | |
| >28 | 1525 (12) | 635 (14) | 890 (11) | |
| Sex (female) | 5245 (43) | 1900 (43) | 3345 (43) | 0·70 |
| Reported born premature | 2970 (24) | 1019 (23) | 1951 (25) | 0·005 |
| Reported low birth weight | 1782 (15) | 581 (13) | 1201 (15) | <0·001 |
| Anthropometry | | | | |
| Weight (kg) | | | | |
| <1·5 | 1767 (14) | 566 (13) | 1201 (15) | <0·001 |
| 1·5 to <2·5 | 3211 (26) | 1128 (26) | 2083 (27) | |
| ≥2·5 | 7193 (59) | 2684 (61) | 4509 (57) | |
| Missing | 100 (0·8) | 43 (1·0) | 57 (0·7) | |
| MUAC (cm) | | | | |
| <9 | 3933 (32) | 1342 (30) | 2591 (33) | <0·001 |
| 9 to 10 | 2492 (20) | 862 (20) | 1630 (21) | |
| 10 to 11 | 2926 (24) | 1035 (23) | 1891 (24) | |
| ≥11 | 2622 (21) | 1056 (24) | 1566 (20) | |
| Missing | 298 (2·4) | 126 (2·9) | 172 (2·2) | |
| Clinical features | | | | |
| Axillary temperature | | | | <0·001 |
| <36˚C | 3553 (29) | 1358 (31) | 2195 (28) | |
| 36 to 37•5˚C | 4692 (38) | 1711 (39) | 2981 (38) | |
| >37·5˚C | 3948 (32) | 1318 (30) | 2630 (34) | |
| Respiratory rate/min[b] | | | | |
| Bradypnoea | 540 (4·4) | 188 (4·3) | 352 (4·5) | 0·56 |
| Normal | 7333 (60) | 2647 (60) | 4686 (60) | |
| Tachypnoea | 4158 (34) | 1490 (34) | 2668 (34) | |
| Missing | 240 (2·0) | 96 (2·2) | 144 (1·8) | |
| Heart rate/min[c] | | | | |
| Bradycardia | 396 (3·2) | 158 (3·6) | 238 (3·0) | 0·11 |
| Normal | 8162 (67) | 2910 (66) | 5252 (67) | |
| Tachycardia | 3667 (30) | 1331 (30) | 2336 (30) | |
| Missing | 46 (0·4) | 22 (0·5) | 24 (0·3) | |
| Hypoxia[d] | 2668 (22) | 932 (21) | 1736 (22) | 0·19 |
| Lower chest wall indrawing | 5562 (45) | 2051 (46) | 3511 (45) | 0·13 |
| Wheeze | 112 (0·9) | 46 (1·0) | 66 (0·8) | 0·41 |



For peer review only - http://bmjopen.bmj.com/site/about/guidelines.xhtml

| | | | | |
|---|---|---|---|---|
| Stridor | 62 (0·5) | 19 (0·4) | 43 (0·6) | 0·48 |
| Breathing difficulty | 5966 (49) | 2172 (49) | 3794 (48) | 0·44 |
| Cyanosis | 560 (4·6) | 210 (4·8) | 350 (4·5) | 0·54 |
| Capillary refill >2 seconds | 301 (2·6) | 105 (2·4) | 196 (2·5) | 0·81 |
| Temperature gradient | 710 (5·8) | 258 (5·8) | 452 (5·8) | 0·73 |
| Weak pulse | 463 (3·8) | 157 (3·6) | 306 (3·9) | 0·05 |
| Lethargy | 971 (7·9) | 325 (7·4) | 646 (8·2) | 0·15 |
| Impaired consciousness[e] | 792 (6·5) | 250 (5·7) | 542 (6·9) | 0·007 |
| Bulging fontanel | 111 (0·9) | 32 (0·7) | 79 (1·0) | 0·21 |
| Stiff neck | 48 (0·4) | 10 (0·2) | 38 (0·5) | 0·05 |
| Convulsions | 689 (5·6) | 197 (4·5) | 492 (6·3) | <0·001 |
| Sunken eyes | 134 (1·1) | 44 (1·0) | 90 (1·2) | 0·44 |
| Reduced skin turgor | 308 (2·5) | 97 (2·2) | 211 (2·7) | 0·19 |
| Pallor | 633 (5·2) | 221 (5·0) | 412 (5·3) | 0·55 |
| **Laboratory features** | | | | |
| Meningitis [f] | 98 (0·8) | 33 (0·8) | 65 (0·8) | 0·87 |
| Haemoglobin <11 g/dl)[g] | 1207 (9·8) | 476 (11) | 731 (9·3) | 0·02 |
| HIV antibody positive | 441 (3·6) | 142 (3·2) | 299 (3·8) | 0·11 |
| Malaria slide positive | 5 (0·04) | 4 (0·09) | 1 (0·01) | 0·02 |
| Bacteraemia | 590 (4·8) | 170 (3·9) | 420 (5·4) | <0·001 |
| White blood cells ($10^{12}$ cells/L)[h] | | | | |
| <4 | 134 (1·1) | 54 (1·2) | 80 (1·0) | <0·001 |
| 4–20 | 8738 (71) | 3228 (73) | 5510 (70) | |
| >20 | 2202 (18) | 690 (16) | 1512 (19) | |
| unavailable | 1197 (9·8) | 449 (10) | 748 (9·5) | |
| Platelets ($10^9$ cells/L)[i] | | | | |
| <150 cells/L | 1615 (13) | 586 (13) | 1029 (13) | 0·59 |
| ≥150 | 9455 (77) | 3387 (77) | 6068 (77) | |
| unavailable | 1201 (9·8) | 448 (10) | 753 (9·6) | |
| Blood glucose (mmols/L) | | | | |
| <2·6 | 2479 (20) | 882 (20) | 1597 (20) | 0·29 |
| 2·6 to 7·0 | 5086 (41) | 1875 (42) | 3211 (41) | |
| >7·0 | 688 (5·6) | 231 (5·2) | 457 (5·8) | |
| unavailable | 4018 (33) | 1433 (32) | 2585 (33) | |

[a]-Eligible admissions were young infants aged <60days admitted from 2009 to 2019, [b]- Tachypnoea: respiratory rate ≥60 breaths/min, Bradypnoea: respiratory rate <30 breaths/min, [c]-Tachycardia: heart rate>160 beats/min, Bradycardia: heart rate<100 beats/min, [d]-Hypoxia: oxygen saturation<90%,[e]-Impaired consciousness level if 'prostrate' or 'unconscious', [f] Meningitis: positive CSF culture, or positive CSF microscopy, or positive CSF antigen test, or elevated CSF WBC count (≥20 in young infants aged 0-28 days OR, ≥10 in young infants aged 29-59 days) PLUS a positive blood culture for a known pathogen, [g] Anaemia: haemoglobin <11 g/dl, [h] Normal values WBC 4-20 x $10^{12}$ cells/L, Leucopoenia WBC <4 x $10^{12}$ cells/L, Leucocytosis WBC >20 x $10^{12}$ cells/L, [I] Normal values Platelets ≥150x$10^9$ cells/L, Thrombocytopenia <150x$10^9$ cells/L, KHDSS: Kilifi Health and Demographic Surveillance System, MUAC: Mid-upper arm circumference.



For peer review only - http://bmjopen.bmj.com/site/about/guidelines.xhtml

Admissions over time

The annual number of admissions are shown in **Supplementary Table S6.** The overall proportion of YI among all admissions under 5 years old was 28% (95%CI 27–29%), increasing from 19% in 2009 to 34% in 2019 (test of linear trend P=0·02) **Figure 2**. **Figure 3A** shows the upward trend of absolute YI admissions and downward trends for 2 to 59-month-olds and all admissions <60 months old (all P-values for tests for stationarity <0·05). There was no significant difference in monthly YI admissions before introduction of free maternity care in June 2013 (monthly median [IQR] of 76 [66–96] admissions) and after June 2013 (monthly median [IQR] of 95 [78–125] admissions) season-adjusted IRR 1·06 (95%CI 0·54–2·09) P=0·86 (**Supplementary Figure S1A**). The mean monthly YI admissions on day of birth did not differ before and after June 2013; season-adjusted IRR 0·88 (95%CI 0·44 to 1·76), P=0·72. The proportion of YI admissions to total admissions aged <60 months before and after June 2013 were not different; season-adjusted IRR 1·02 (95%CI 0·28–3·71) P=0·97 **Figure 3D**. We found no significant difference in monthly absolute admissions (all admissions <60 months old), before and after June 2013; season-adjusted IRR 1·01 (95%CI 0·51–2·00) P=0·97 (**Supplementary Figure S1B)**.

Inpatient deaths

Overall, 1,914/11,877 (16%) of YI died in hospital. The risk of inpatient death was not significantly different between 645/4,272 (15%) KHDSS residents and 1,269/7,605 (17%) non-residents of KHDSS (age- and sex-adjusted SHR 0·93 (95%CI 0·85–1·02) P=0·12) (**Figure 1**). The annual YI inpatient case fatality ratio was stable (11% in 2009 and 13% in 2019. P-value for trend=0·80), **Figure 2**. Monthly inpatient case fatality for YI, 2 to 59 months old and all <60 months old children are shown in **Figure 3B.**



For peer review only - http://bmjopen.bmj.com/site/about/guidelines.xhtml

During the study period there were 3,119 inpatient deaths among admissions <60 months old admitted at KCH, with YI admissions accounting for 61% (95%CI 60–63%) of the deaths and no significant linear trend from 2009 to 2019 (trend P=0·29). The mean monthly YI inpatient case fatality was 16% (sd 0·86) and 16% (sd 1·23) before and after June 2013 respectively; season-adjusted IRR 0·77 (95%CI 0·39–1·52) P=0·45 **Figure 3C**. The mean monthly case fatality for all admissions aged <60 months and admissions 2–59 months old did not differ before June 2013 and after June 2013; season-adjusted IRR 0·79 (95%CI 0·39–1·58) P=0·50 and IRR 0·81 (95%CI 0·39–1·69) P=0·57 respectively **Supplementary Figure S1 C** and **D**.

Among the 4,421 KHDSS-resident YI admissions, median [IQR] time to death was 2 [1–4] days, while the survivors were admitted for 5 [3–8] days. A total of 423/645 (66%) deaths occurred within the first 48 hours following admission. Forty-one YI left against medical advice, and 55 were referred to other hospitals for further care.

Admission diagnosis & case fatality ratio

The commonest reasons for hospital admission were neonatal sepsis (47%), preterm complications (20%) and birth asphyxia (16%) accounting for 83% of all YI admissions (**Table 2**). The case fatality ratios for YI with respiratory distress syndrome, preterm complications and birth asphyxia were 52%, 29% and 28% respectively (**Table 2**).



For peer review only - http://bmjopen.bmj.com/site/about/guidelines.xhtml

**Table 2. Discharge diagnosis assigned by clinician.**

| Discharge diagnosis[a] | No. (%) Diagnosis assigned by clinician at discharge | |
|---|---|---|
| | All admissions (N=4421) | Inpatient Deaths (N=645) |
| Neonatal sepsis | 2097 (47) | 201 (9·6) |
| Preterm complications | 889 (20) | 262 (29) |
| Birth asphyxia | 724 (16) | 201 (28) |
| Neonatal jaundice | 611 (14) | 56 (9·2) |
| Lower respiratory tract infection | 486 (11) | 41 (8·4) |
| Respiratory distress syndrome | 263 (6·0) | 136 (52) |
| Congenital anomalies | 215 (4·9) | 55 (26) |
| Meningitis[b] | 112 (2·5) | 11 (9·8) |
| Anaemia | 78 (1·8) | 14 (18) |
| Malnutrition | 36 (0·8) | 1 (2·8) |
| None specified | 69 (1·6) | 4 (5·8) |
| Others | 266 (6·0)[c] | 13 (4·9) |

[a] Young infant could be assigned up to 2 diagnoses

[b] Meningitis: positive CSF culture, or positive CSF microscopy, or positive CSF antigen test, or elevated CSF WBC count (≥20 in young infants aged 0-28 days OR, ≥10 in young infants aged 29-59 days) PLUS a positive blood culture for a known pathogen

[c] Accidents-3, Acute abdominal obstruction-15, bronchiolitis-12, burns-1, Candidiasis-1, Cellulitis abscess-21, Chickenpox-1, Chromosomal abnormality-5, CNS abscess-1, Conjunctivitis-2, Dehydration-2, Dental problems-1, Diabetes-1, Elective surgery-5, Encephalopathy-9, Epilepsy-7, Extra pulmonary TB-1, Febrile convulsions-5, Feeding difficulty-1, Gastroenteritis-15, Haemolytic uraemic syndrome-1, Hydrocephalus-11, LTB/croup-1, Immunosuppression-17, Malaria-2, Male genital problem-1, Meconium aspiration-33, Neonatal haemorrhage-14, Neonatal tetanus -10, Other skin disease-3, Otitis media-1, Poisoning (organophosphates)-1, Pyogenic arthritis-1, Rabies-1, Rash-4, renal failure-6, trauma/fractures/RTA-11, Urinary tract infection-10, upper respiratory tract infection (URTI)-24, Viral hepatitis-2, Viral infection-3.

Exposures associated with inpatient death

Variables assessed for association with inpatient death in univariate models are shown in **Supplementary Table S3**. In the multivariable analysis (**Table 3**), admissions at age ≤2 days and 3–7 days, compared to ≥28 days old, were associated with inpatient deaths. Very low admission weight (<1·5kg) and weight 1·5-2·4kg compared to ≥2·5kg were positively associated with inpatient deaths. Signs of clinical severity (bradypnoea, tachypnoea, bradycardia, hypoxia, lower chest wall indrawing, breathing difficulty, weak pulse, impaired consciousness and hypothermia, but not fever), meningitis, bacteraemia, leucopoenia and leucocytosis but not an HIV antibody



For peer review only - http://bmjopen.bmj.com/site/about/guidelines.xhtml

positive test (aSHR 1·15 (95%CI 0·81–1·63)) were positively associated with inpatient death. The multivariable model bootstrapped AUC was 0·88 (95%CI 0·86–0·89) **Table 3**.

**Table 3. Multivariable regression analysis of factors associated with inpatient and post-discharge mortality.**

| | Inpatient analysis | | Post-discharge analysis | |
|---|---|---|---|---|
| | **Adjusted SHR*** | **P-value** | **Adjusted HR** | **P-value** |
| **Demographics** | | | | |
| Age in days | | | | |
| 0 to 2 | 2·12 (1·46–3·06) | <0·001 | 0·78 (0·50–1·21) | 0·27 |
| 3 to 7 | 3·88 (2·46–6·10) | <0·001 | 0·57 (0·30–1·08) | 0·08 |
| 8 to 28 | 1·42 (0·90–2·25) | 0·13 | 1·19 (0·73–1·93) | 0·48 |
| >28 | Reference | | Reference | |
| Sex (female) | 0·91 (0·78–1·07) | 0·26 | 0·98 (0·74–1·31) | 0·91 |
| Admission days (log) | ¶ | | 1·93 (1·52–2·46) | <0·001 |
| Type of discharge | | | | |
| Normal | ¶ | | Reference | |
| Absconded | ¶ | | 2·73 (1·04–7·18) | 0·04 |
| Transferred/referred | ¶ | | 14·4 (9·22–22·6) | <0·001 |
| **Anthropometry** | | | | |
| Weight (kg) | | | | |
| <1.5 | 2·16 (1·75–2·67) | <0·001 | 2·01 (1·41–2·87) | <0·001 |
| 1.5 to <2.5 | 1·42 (1·16–1·74) | 0·001 | 0·88 (0·47–1·65) | 0·69 |
| ≥2.5 | Reference | | Reference | |
| Missing weight | 3·85 (2·59–5·71) | <0·001 | - | |
| **Clinical features** | | | | |
| Axillary temperature | | | | |
| <36˚C | 1·44 (1·17–1·78) | 0·001 | 1·06 (0·72–1·57) | 0·76 |
| 36 to 37·5˚C | Reference | | Reference | |
| >37·5˚C | 1·09 (0·84–1·41) | 0·53 | 0·67 (0.46–0·99) | 0·04 |
| Missing temperature | 1·03 (0·38–2·75) | 0·96 | 0·94 (0·09–9·30) | 0·96 |
| Respiratory rate/min | | | | |
| Bradypnoea | 1·45 (1·09–1·93) | 0·01 | 1·72 (0·81–3·65) | 0·16 |
| Normal | Reference | | Reference | |
| Tachypnoea | 0·80 (0·67–0·95) | 0·01 | 1·33 (0·98–1·79) | 0·07 |
| Missing | 1·51 (0·64–3·56) | 0·34 | 0·82 (0·10–6·49) | 0·85 |
| Heart rate/min | | | | |
| Bradycardia | 1·40 (1·08–1·82) | 0·01 | ¶ | |
| Normal | Reference | | | |
| Tachycardia | 1·14 (0·94–1·37) | 0·18 | ¶ | |
| Missing | 0·41 (0·03–5·13) | 0·49 | ¶ | |
| Hypoxia (SaO2 <90%) | 1·62 (1·37–1·92) | <0·001 | ¶ | |
| Capillary refill >2 seconds | 1·34 (0·97–1·86) | 0·08 | ¶ | |



For peer review only - http://bmjopen.bmj.com/site/about/guidelines.xhtml

| | | | | |
|---|---|---|---|---|
| Lower chest wall indrawing | 1·41 (1·14–1·75) | 0·002 | ¶ | |
| Stridor | 1·93 (0·92–4·03) | 0·08 | ¶ | |
| Breathing difficulty | 1·45 (1·15–1·82) | 0·001 | ¶ | |
| Weak pulse | 1·61 (1·19–2·17) | 0·002 | 2·33 (1·13–4·82) | 0·02 |
| Bulging fontanel | 2·45 (0·91–6·65) | 0·08 | 3·41 (1·49–7·79) | 0·004 |
| Impaired consciousness | 2·21 (1·72–2·84) | <0·001 | ¶ | |
| Pallor | 1·30 (0·98–1·71) | 0·07 | ¶ | |
| **Laboratory features** | | | | |
| Meningitis | 5·45 (2·50–11·8) | <0·001 | 2·21 (0·95–5·13) | 0·07 |
| HIV antibody positive | 1·15 (0·81–1·63) | 0·43 | 1·09 (0·50–2·35) | 0·83 |
| Bacteraemia | 2·21 (1·51–3·22) | <0·001 | ¶ | |
| White blood cells ($10^{12}$ cells/L) | | | | |
| <4 | 2·17 (1·30–3·62) | 0·003 | ¶ | |
| 4-20 | Reference | | ¶ | |
| >20 | 1·71 (1·43–2·04) | <0·001 | ¶ | |
| unavailable | 1·09 (0.82–1·44) | 0·57 | ¶ | |
| Model performance | | | | |
| Bootstrapped AUC (95% CI) | 0·88 (0·86–0·89) | | 0·76 (0·72–0·79) | |

**SHR;** sub-distribution hazard ratios; *the SHR are from the Fine and Gray's proportional sub-hazards model, HR-Hazard ratio from the Proportional Cox regression model, **¶;** variables not selected for inclusion in the multivariable model, AUC; area under receiver operating characteristics. Meningitis: positive CSF culture, or positive CSF microscopy, or positive CSF antigen test, or elevated CSF WBC count (≥20 in young infants aged 0-28 days OR, ≥10 in young infants aged 29-59 days) PLUS a positive blood culture for a known pathogen

Post-discharge death

There were 3,776 live discharges from 3,640 YI residents of KHDSS, of which 3,760 (from 3,625 individual YI) were followed up for 3,233 infant-years (**Figure 1**). During one-year follow-up, there were 208/3625 (5·7%) deaths: 64·3 (95%CI 56·2–73·7) deaths per 1,000 infant-years. The median [IQR] time to death after discharge was 35 [7–92] days. Of the 208 post-discharge deaths, 101 (49%), 160 (77%), 179 (86%) and 193 (93%) occurred within 1, 3, 6 and 9 months after discharge respectively. The annual YI post-discharge case fatality was 5·4% in 2009 and 6·3% in 2019 without evidence of linear trend (P-value for trend=0·77) (**Figure 2)**.



For peer review only - http://bmjopen.bmj.com/site/about/guidelines.xhtml

One hundred and one (49%) of the 208 post-discharge deaths occurred at home without hospital readmission, 67 (32%) occurred during readmission to KCH and 40 (19%) occurred at other health facilities. The five leading assigned causes of deaths for the 67 deaths at KCH were: neonatal sepsis (24%), preterm complications (22%), congenital heart disease (15%), neonatal jaundice (7·4%) and meningitis (7·4%) which were similar to index admission diagnosis **Supplementary Table S7**. Causes of other deaths were unknown.

Overall, we observed 853 (20%) deaths among 4,272 individual YIs: 645 inpatient and 208 post-discharge, hence 24% of deaths were post-discharge.

Exposures assessed for association with post-discharge mortality are shown on **Table 3**. In the multivariable Cox regression model, log days of hospital admission, leaving against advice, and referral to more specialized hospital were positively associated with post-discharge mortality. Other exposures associated with post-discharge mortality were low admission weight, fever, weak pulse, and bulging fontanel, whilst a meningitis diagnosis at admission had borderline effect (**Table 3**). The multivariable model bootstrapped AUC was 0·76 (95%CI 0·72–0·79).

Subgroup analysis

In a subgroup analysis including 1,358 admissions of YIs born at KCH, their median [IQR] gestational age was 38 (36–40) weeks and birth weight 2,778 (2,000-3,195) grams respectively. In the univariate regression model, born premature, born low birth weight and birth weight Z score <-2 were positively associated with inpatient mortality (**Supplementary Table S8**). In the multivariable model, low birth weight, admission age <8 days, bacteraemia and signs of clinical severity were associated with inpatient mortality (**Supplementary Table S9**).



For peer review only - http://bmjopen.bmj.com/site/about/guidelines.xhtml

For peer review only

Among the 1,142 YI followed up for 1,021 child-years of which 41/1,142 (3·6%) died, low birth weight (aHR 2·76 (95%CI 1·30–5·82)) was positively associated with post-discharge mortality in the multivariable model (**Supplementary Table S9**).

For peer review only



For peer review only - http://bmjopen.bmj.com/site/about/guidelines.xhtml

## Discussion

Trends in admissions and proportions of young infants

During the study period, we observed a marked increase in YI admissions and proportion of YI among admissions in under-fives increased from around a fifth in 2009, to more than one-third in 2019. However, this did not seem to be associated with the introduction of free maternity care in 2013. Lack of observable effect may be due to challenges faced during policy implementation arising from inadequate expansion of quality health care facilities and resources. Several authors reported an increase in mothers attending Kenyan health facilities for antenatal care and delivery,(11, 21) however our results suggest this occurred in the context of a general trend which we previously observed during 1990-2008.(10)

Conversely, the number of admitted children older than 60 days decreased alongside a reduction in local malaria transmission,(22) introduction of routine childhood pneumococcal conjugate and rotavirus immunisation,(23) and expansion in numbers of health facilities in Kilifi County.(24) Variation in annual admissions over the years was due to multiple health workers' strikes.(13) During these periods, the general paediatric ward was closed and only the sickest children were admitted to the paediatric HDU due to limited staffing and bed capacity. The time series analysis indicated an increase in inpatient mortality during strikes (**Figure 3C)**.

The leading diagnoses at admission in our analysis were neonatal sepsis, preterm complications, and birth asphyxia, similar to the period 1990–2008.(10) Over a third of admissions from KCH maternity were preterm and the hospital also received referrals of preterm and very low birthweight infants from sub-county hospitals and local health centres. There are few African



For peer review only - http://bmjopen.bmj.com/site/about/guidelines.xhtml

published datasets of neonatal or YI inpatient diagnoses; in a network of 7 Nigerian and Kenyan hospitals, prematurity accounted for over half (52%), and birth asphyxia almost a quarter (24%) of neonatal admissions.(25) The leading bacterial isolates from blood cultures in our study (*Klebsiella pneumoniae, Escherichia coli*, *Staphylococcus aureus*) were similar to those among YI in rural settings of Tanzania and Burkina Faso.(26) Kenya attained elimination status of maternal and neonatal tetanus in 2018, following immunisation campaigns in high-risk regions.(27) Compared to 1990–2008,(10)neonatal tetanus was uncommon at our centre with only 10 cases in 11 years.

For peer review only

### Inpatient deaths

The WHO has reported that in 2019, “47% of all under-5 deaths occurred in the newborn period with about one third dying on the day of birth and close to three quarters dying within the first week of life”.(28) We found YI accounted for more than 60% of under-fives inpatient deaths, similar to a retrospective study of 16 Kenyan public hospitals in which neonatal deaths comprised 66% of inpatient paediatric deaths.(5) We found respiratory distress syndrome, birth asphyxia and preterm complications had the highest inpatient mortality. Improvements in peripartum care of mothers and infants together with appropriate technology such as non-invasive ventilation for management of respiratory complications of preterm birth are priorities for reduction in neonatal mortality in hospitals in LMICs.(5)

### Post-discharge deaths

Less than a quarter (24%) of all deaths during 1-year of follow up occurred post-discharge. This reflects a high inpatient (16%) case fatality rate with many very early inpatient deaths compared



For peer review only - http://bmjopen.bmj.com/site/about/guidelines.xhtml

to 6·6% in children aged ≥60 days.(7) Nevertheless, the post-discharge YI mortality rate (64·3 per 1,000 child/years) was more than twice that of a cohort of children aged 2–59 months admitted to KCH between 2007- 2015.(29) This reflects post-discharge mortality rates being highest in younger age groups, such as in Tanzania among under 1-year olds: 72 per 1,000 child/years (95%C.I. 67·2–77·2) falling to 6·9 (95%C.I. 5·5–8·7) per 1,000 child/years in 4 to <5 year olds.(30)

A greater proportion of YI post-discharge deaths occurred in hospital than among older children,(7) implying that caregivers may be more likely to seek re-admission for YI or may live closer to KCH. About half of post-discharge deaths occurred within the first month, highlighting the need for formal 'down-referral' for continuity of care after discharge in high risk YI.

Analysis of exposures revealed that some were common for both inpatient and post-discharge mortality: low admission weight, axillary temperature, and respiratory rate. Birth weight was not available for most YI but low admission weight <2·5kg was common (40%) in our participants. Of known causes of post-discharge deaths, leading ones were related to problems in the early neonatal period. Meningitis was among the top 5 causes and bulging fontanel noted at admission was associated with increased risk of post-discharge death, suggesting that current treatment guidelines may not be sufficiently effective.

**Strengths and limitations of the study**

Strengths of this study are large sample size, systematic collection of data and linkage to a well-established demographic surveillance system, with few losses to follow up. Limitations are lack of accurate gestational age estimation and unknown birthweight of most participants. We did not have data collected at discharge, which may be of value in taking a risk-based approach to

For peer review only

post-discharge care. This analysis is from a single hospital and outcomes in other settings with a different patient profile and facilities may vary.

## Conclusions

Neonatal and YI admissions account for an increasing proportion of inpatient paediatric admissions, and their mortality rate remains high. Mortality reduction will depend on improvements in antenatal, peripartum and postpartum care of mothers and infants, as well as implementation of standardized neonatal care and paediatric protocols. Post-discharge mortality rates are higher, but account for a lower proportion of all deaths than among children age ≥60 days, likely because of the predominance of fatal conditions soon after birth with a correspondingly substantial proportion of infant mortality occurring in the first week of life.



For peer review only - http://bmjopen.bmj.com/site/about/guidelines.xhtml

Acknowledgements

We thank the parents, patients, and staff of Kilifi County Hospital and the KEMRI-Wellcome Trust Research Programme for their participation in the study. This study is published with the permission of the Director, KEMRI. For the purpose of open access, the authors have applied a CC BY public copyright license to any author-accepted manuscript version arising from this submission.

I, the Submitting Author has the right to grant and does grant on behalf of all authors of the Work (as defined in the below author licence), an exclusive licence and/or a non-exclusive licence for contributions from authors who are: i) UK Crown employees; ii) where BMJ has agreed a CC-BY licence shall apply, and/or iii) in accordance with the terms applicable for US Federal Government officers or employees acting as part of their official duties; on a worldwide, perpetual, irrevocable, royalty-free basis to BMJ Publishing Group Ltd ("BMJ") its licensees and where the relevant Journal is co-owned by BMJ to the co-owners of the Journal, to publish the Work in BMJ Global Health and any other BMJ products and to exploit all rights, as set out in our licence.

The Submitting Author accepts and understands that any supply made under these terms is made by BMJ to the Submitting Author unless you are acting as an employee on behalf of your employer or a postgraduate student of an affiliated institution which is paying any applicable article publishing charge ("APC") for Open Access articles. Where the Submitting Author wishes to make the Work available on an Open Access basis (and intends to pay the relevant APC), the terms of reuse of such Open Access shall be governed by a Creative Commons licence – details of these licences and which Creative Commons licence will apply to this Work are set out in our licence referred to above.

For peer review only

443 **Figures**

444 **Figure 1. Flow of study participants.**

445 **Figure 2. Annual proportion of YI admissions to all admissions <60 months, inpatient case fatality ratio (CFR) and post-discharge CFR.**

447 Proportions are plotted with 95% confidence intervals.

448 **Figure 3. A: Monthly hospital admissions (with predicted mean temporal trend), B: Monthly case fatality rates (with predicted mean temporal trend), C: Monthly young infant inpatient case fatality before and after June 2013 and D: Monthly proportions of young infants to admissions <60 months old.**

452

453

454 Ethics approval and consent to participate

455 Written consent was provided by the caregivers of all the surveillance study participants. Ethical approval to conduct this analysis was granted by the Kenya Medical Research Institute (KEMRI) National Ethics Review Committee (SCC 2778).

458 Consent for publication – not applicable

459 Availability of data and materials

460 Data are available in a public, open access repository. Deidentified participant data and analysis code have been deposited and may be requested at the Harvard Dataverse via this link https://doi.org/10.7910/DVN/0XJVFX

For peer review only



For peer review only - http://bmjopen.bmj.com/site/about/guidelines.xhtml

Competing interests

JAB declares the following: Chair of the DSMB for **"Efficacy and safety of whole-body chlorhexidine cleansing in reducing bacterial skin colonisation of hospitalised neonates - a pilot trial".** *St George's, University of London and global sites;* Treasurer of the Commonwealth Society for Paediatric Gastroenterology & Nutrition Other authors declare they have no competing interests.

Funding

Authors NM, AN, NO, MO, and staffing, facilities, and resources were funded by the Wellcome Trust (203077_Z_16_Z). MM was supported by a Wellcome Trust International Intermediate Fellowship (221997/Z/20/Z). JAB was supported by the Medical Research Council–Department for International Development–Wellcome Trust Joint Global Health Trials scheme (MR/M007367/1). JAB and MN were supported by the Bill & Melinda Gates Foundation (OPP1131320). CWO was supported by the Drugs for Neglected Diseases initiative/Global Antibiotic Research and Development Partnership (OXF-DND02). AT was supported by Crosslinks.

Role of the funding source

The funders did not have a role in study design, in the collection, analysis, and interpretation of data, in writing the report, or in the decision to submit the paper for publication.

Author contributions

AT: Conceptualization, investigation, methodology, formal analysis, writing – original draft, writing– review & editing; MN: Conceptualization, methodology, data curation, formal analysis, visualisation , writing – original draft, writing– review & editing; CWO: Conceptualization,

For peer review only

investigation, methodology, formal analysis, validation, writing – original draft, writing– review & editing; AN: Investigation, methodology, project administration, writing– review & editing; MM: Conceptualization, methodology, writing– review & editing; NM: Investigation, project administration, funding acquisition, resources, supervision, writing– review & editing; NO: Data curation, writing– review & editing, MO: Data curation, writing– review & editing; JAB: Conceptualisation, investigation, methodology, funding acquisition, supervision, validation, writing– review & editing.

AT and MN contributed equally to this paper. AT, the guarantor, accepts full responsibility for the finished work and the conduct of the study, had access to the data, and controlled the decision to publish.

## References

1. UNICEF . Levels & Trends in Child Mortality: Report 2020, Estimates developed by the United Nations Inter-agency Group for Child Mortality Estimation. New York: United Nations Children's Fund; 2020.

2. Collaborators GBDCM. Global, regional, national, and selected subnational levels of stillbirths, neonatal, infant, and under-5 mortality, 1980-2015: a systematic analysis for the Global Burden of Disease Study 2015. *Lancet*. 2016;388(10053):1725-74 doi 10.1016/S0140-6736(16)31575-6.

3. English M, Ngama M, Musumba C, et al. Causes and outcome of young infant admissions to a Kenyan district hospital. *Arch Dis Child*. 2003;88(5):438-43 doi 10.1136/adc.88.5.438.



For peer review only - http://bmjopen.bmj.com/site/about/guidelines.xhtml

506 4. van den Boogaard W, Manzi M, Harries AD, et al. Causes of pediatric mortality and case-fatality rates in eight Médecins Sans Frontières-supported hospitals in Africa. *Public Health Action*. 2012;2(4):117-21 doi 10.5588/pha.12.0038.

509 5. Irimu G, Aluvaala J, Malla L, et al. Neonatal mortality in Kenyan hospitals: a multisite, retrospective, cohort study. *BMJ Glob Health*. 2021;6(5) doi 10.1136/bmjgh-2020-004475.

511 6. Obiero CW, Mturi N, Mwarumba S, et al. Clinical features to distinguish meningitis among young infants at a rural Kenyan hospital. *Arch Dis Child*. 2021;106(2):130-6 doi 10.1136/archdischild-2020-318913.

514 7. CHAIN Network. Childhood mortality during and after acute illness in Africa and south Asia: a prospective cohort study. *Lancet Glob Health*. 2022;10(5):e673-e84 doi 10.1016/s2214-109x(22)00118-8.

517 8. Nemetchek B, English L, Kissoon N, et al. Paediatric postdischarge mortality in developing countries: a systematic review. *BMJ open*. 2018;8(12):e023445 doi 10.1136/bmjopen-2018-023445.

519 9. Moisi JC, Gatakaa H, Berkley JA, et al. Excess child mortality after discharge from hospital in Kilifi, Kenya: a retrospective cohort analysis. *Bull World Health Organ*. 2011;89(10):725-32, 32A doi 10.2471/BLT.11.089235.

522 10. Mwaniki MK, Gatakaa HW, Mturi FN, et al. An increase in the burden of neonatal admissions to a rural district hospital in Kenya over 19 years. *BMC Public Health*. 2010;10:591 doi 10.1186/1471-2458-10-591.

525 11. Lang'at E, Mwanri L, Temmerman M. Effects of implementing free maternity service policy in Kenya: an interrupted time series analysis. *BMC Health Serv Res*. 2019;19(1):645 doi 10.1186/s12913-019-4462-x.



For peer review only

For peer review only - http://bmjopen.bmj.com/site/about/guidelines.xhtml

12. KEMRI-Wellcome Trust Research Programme. Health-research-linked-to-a-demographic-surveillance-system [Available from: https://kemri-wellcome.org/programme/health-research-linked-to-a-demographic-surveillance-system/ [Accessed May 10 2022].

13. Ong'ayo G, Ooko M, Wang'ondu R, et al. Effect of strikes by health workers on mortality between 2010 and 2016 in Kilifi, Kenya: a population-based cohort analysis. *Lancet Glob Health*. 2019;7(7):e961-e7 doi 10.1016/s2214-109x(19)30188-3.

14. Berkley JA, Lowe BS, Mwangi I, et al. Bacteremia among children admitted to a rural hospital in Kenya. *N Engl J Med*. 2005;352(1):39-47 doi 10.1056/NEJMoa040275.

15. Ministry of Health, Kenya. Basic Paediatric Protocols 4th ed2016.

16. [dataset] Ngari M, Talbert A, Ouma N, et al. Replication Data for: Inpatient and post-discharge mortality among young infants admitted to a rural Kenyan hospital. Harvard Dataverse. 2022. Available from: https://doi.org/10.7910/DVN/0XJVFX.

17. Cuzick J. A Wilcoxon-type test for trend. *Stat Med*. 1985;4(1):87-90 doi 10.1002/sim.4780040112.

18. Fine JP, Gray RJ. A Proportional Hazards Model for the Subdistribution of a Competing Risk. *J Am Stat Assoc*. 1999;94(446):496-509 doi 10.1080/01621459.1999.10474144.

19. Seale AC, Barsosio HC, Koech AC, et al. Embedding surveillance into clinical care to detect serious adverse events in pregnancy. *Vaccine*. 2015;33(47):6466-8 doi 10.1016/j.vaccine.2015.07.086.

20. Papageorghiou AT, Kennedy SH, Salomon LJ, et al. The INTERGROWTH-21(st) fetal growth standards: toward the global integration of pregnancy and pediatric care. *Am J Obstet Gynecol*. 2018;218(2S):S630-S40 doi 10.1016/j.ajog.2018.01.011.

21. Orangi S, Kairu A, Malla L, et al. Impact of free maternity policies in Kenya: an interrupted time-series analysis. *BMJ Glob Health*. 2021;6(6) doi 10.1136/bmjgh-2020-003649.



For peer review only - http://bmjopen.bmj.com/site/about/guidelines.xhtml

551 22. Njuguna P, Maitland K, Nyaguara A, et al. Observational study: 27 years of severe malaria
552 surveillance in Kilifi, Kenya. *BMC Med*. 2019;17(1):124 doi 10.1186/s12916-019-1359-9.
553 23. Hammitt LL, Etyang AO, Morpeth SC, et al. Effect of ten-valent pneumococcal conjugate vaccine
554 on invasive pneumococcal disease and nasopharyngeal carriage in Kenya: a longitudinal surveillance
555 study. *Lancet (London, England)*. 2019;393(10186):2146-54 doi 10.1016/s0140-6736(18)33005-8.
556 24. Munywoki J, Kagwanja N, Chuma J, et al. Tracking health sector priority setting processes and
557 outcomes for human resources for health, five-years after political devolution: a county-level case study
558 in Kenya. *Int J Equity Health*. 2020;19(1):165 doi 10.1186/s12939-020-01284-3.
559 25. Nabwera HM, Wang D, Tongo OO, et al. Burden of disease and risk factors for mortality amongst
560 hospitalized newborns in Nigeria and Kenya. *PLoS One*. 2021;16(1):e0244109 doi
561 10.1371/journal.pone.0244109.
562 26. Mduma E, Halidou T, Kaboré B, et al. Etiology of severe invasive infections in young infants in
563 rural settings in sub-Saharan Africa. *PLoS One*. 2022;17(2):e0264322 doi 10.1371/journal.pone.0264322.
564 27. WHO Afro. Kenya now eliminates maternal and neonatal tetanus 2019 [Available from:
565 https://www.afro.who.int/news/kenya-now-eliminates-maternal-and-neonatal-tetanus[Accessed
566 February 2 2022].
567 28. World Health Organization. Newborns: improving survival and well-being 2020 [Available from:
568 https://www.who.int/news-room/fact-sheets/detail/newborns-reducing-mortality [Accessed May 13
569 2022].
570 29. Talbert A, Ngari M, Bauni E, et al. Mortality after inpatient treatment for diarrhea in children: a
571 cohort study. *BMC Med*. 2019;17(1):20 doi 10.1186/s12916-019-1258-0.
572 30. Mukasa O, Masanja H, DeSavigny D, et al. A cohort study of survival following discharge from
573 hospital in rural Tanzanian children using linked data of admissions with community-based demographic
574 surveillance. *Emerg Themes Epidemiol*. 2021;18(1):4 doi 10.1186/s12982-021-00094-4.



For peer review only - http://bmjopen.bmj.com/site/about/guidelines.xhtml

For peer review only



For peer review only - http://bmjopen.bmj.com/site/about/guidelines.xhtml

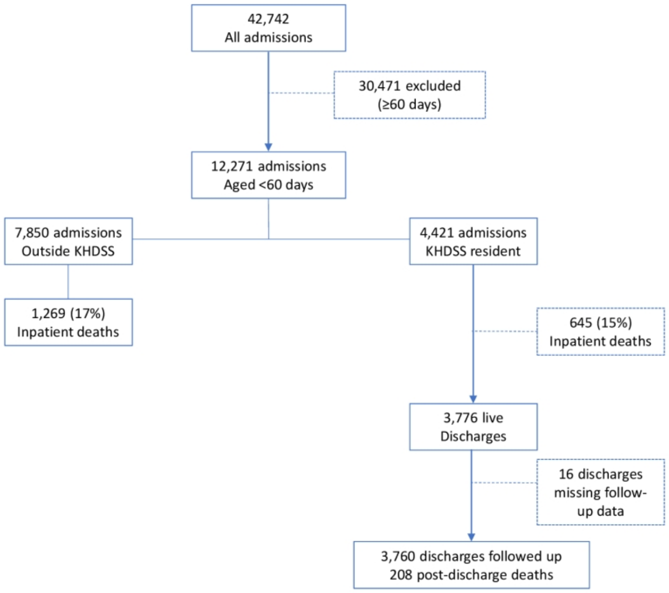


275x190mm (200 x 200 DPI)

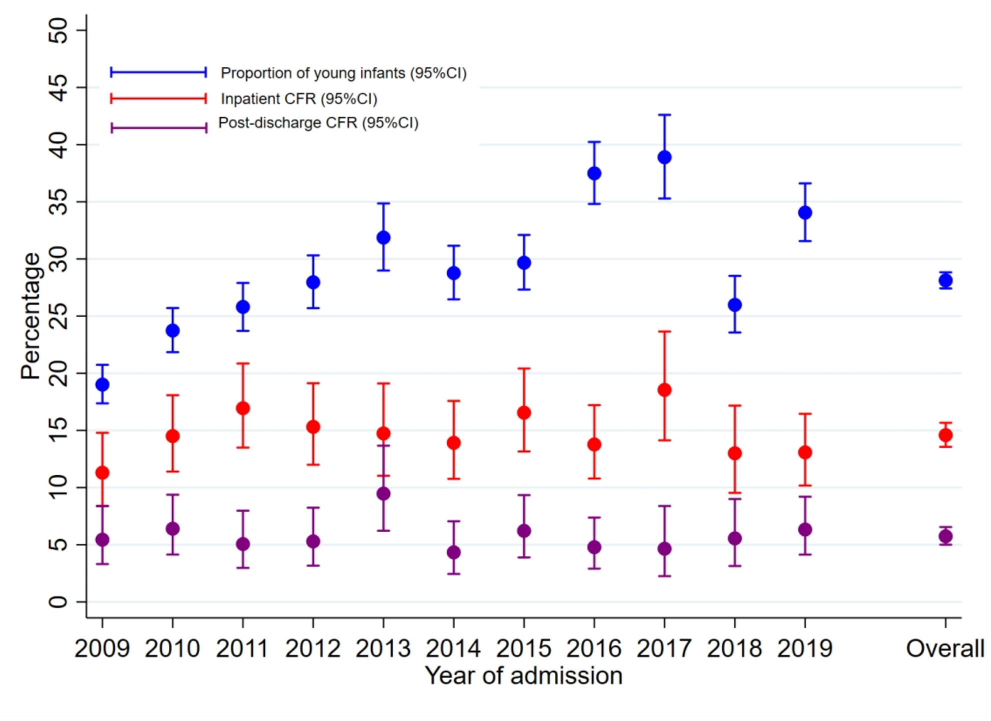


547x398mm (72 x 72 DPI)

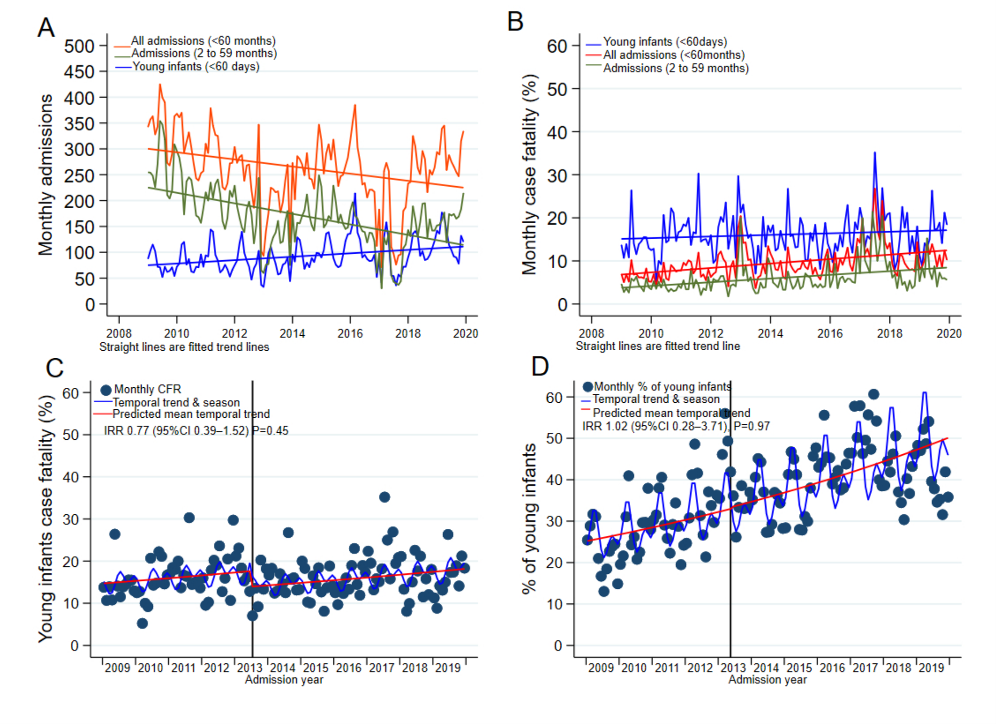


399x290mm (72 x 72 DPI)

**Supplementary materials**

**Table of Contents**


| | |
|---|---|
| Table S1. List of health workers strikes during study period. | Page 2 |
| Table S2. Proportion of missing data | 2,3 |
| Table S3. Univariate analysis of admission features associated with inpatient deaths. | 3, 4 |
| Table S4. Univariate analysis of admission features associated with post-discharge deaths. | 5,6 |
| Table S5 Pathogens isolated from blood and CSF culture of young infants resident of KHDSS during inpatient period. | 7 |
| Table S6. Annual admissions and case fatality ratios | 8 |
| Table S7. Estimated causes of post-discharge deaths during readmission at KCH (67 deaths). | 8 |
| Table S8. Univariate analysis of admission features associated with inpatient deaths among babies born at KCH only. | 9,10 |
| Table S9. Multivariable regression analysis of factors associated with inpatient and post-discharge mortality among children born at KCH only. | 11 |
| Figure S1. A: Monthly all young infant admissions, B: all admissions (<60 months old), C: all admissions (<60 months old) case fatality and D: 2–59 months old case fatality before and after July 2013. | 12 |


For peer review only

Table S1. **List of health workers strikes during study period.**

| Strike dates | Health workers on strike | Duration of strike in days |
|---|---|---|
| 5 to 13 December 2011 | Doctors | 9 |
| 1 to 15 March 2012 | Nurses | 15 |
| 13 September to 4 October 2012 | Doctors | 22 |
| 3 December 2012 to 13 January 2013 | Nurses | 42 |
| 16 January to 11 February 2013 | Nurses | 26 |
| 10 to 23 December 2013 | Doctors & nurses | 14 |
| 5 to 14 December 2016 | Nurses | 11 |
| 5 December 2016 to 15 March 2017 | Doctors | 102 |
| 5 June to 2 November 2017 | Nurses | 150 |

Table S2. **Proportion of missing data**

| **N=4,421** | N missing | % missing |
|---|---|---|
| Demographics | | |
| Age in days | 0 | |
| Sex (female) | 0 | |
| BCG scar | 76 | 1.7 |
| Reported born premature | 321 | 7.3 |
| Reported low birth weight | 322 | 7.3 |
| **Anthropometry** | | |
| Weight (kg) | 43 | 1.0 |
| MUAC (cm) | 126 | 2.9 |
| **Clinical features** | | |
| Axillary temperature | 34 | 0.8 |
| Tachypnea | 208 | 4.7 |
| Tachycardia | 43 | 1.0 |
| Hypoxia (SaO2 <90%) | 20 | 0.5 |
| Lower chest wall indrawing | 68 | 1.5 |
| Wheeze | 69 | 1.6 |
| Stridor | 71 | 1.6 |
| Breathing difficulty | 64 | 1.5 |
| Cyanosis | 70 | 1.6 |
| Capillary refill ≥2 seconds | 34 | 0.8 |
| Temperature gradient | 81 | 1.8 |
| Weak pulse | 71 | 1.6 |
| Lethargy | 70 | 1.6 |
| Impaired consciousness | 70 | 1.6 |
| Bulging fontanel | 72 | 1.6 |
| Stiff neck | 71 | 1.6 |
| Convulsions | 64 | 1.5 |
| Sunken eyes | 73 | 1.7 |
| Reduced skin turgor | 71 | 1.6 |
| Pallor | 70 | 1.6 |
| **Laboratory features** | | |
| HIV antibody positive | 756 | 17 |
| Malaria slide positive | 372 | 8.4 |
| Bacteraemia | 2281 | 52 |


For peer review only - http://bmjopen.bmj.com/site/about/guidelines.xhtml

| | | |
|---|---|---|
| Haemoglobin | 448 | 10 |
| WBC | 449 | 10 |
| Platelets | 448 | 10 |
| Blood glucose (mmols/L) | 1433 | 32 |

Table S3. **Univariate analysis of admission features associated with inpatient deaths.**

| **N=4,421** | **Deaths (N=645) N (%)** | **Crude SHR** | **P-value** | **Scaled Schoenfeld residuals P-value** |
|---|---|---|---|---|
| Demographics | | | | |
| Age in days | | | | |
| 0 to 2 | 511 (19) | 3.31 (2.39–4.58) | <0.001 | 0.14 |
| 3 to 7 | 56 (12) | 2.07 (1.38–3.11) | <0.001 | |
| 8 to 28 | 40 (6.8) | 1.15 (0.74–1.78) | 0.54 | |
| >28 | 38 (6.0) | Reference | | |
| Sex (female) | 268 (14) | 0.99 (0.91–1.09) | 0.93 | 0.77 |
| Reported born premature | 294 (29) | 2.53 (2.32–3.77) | <0.001 | 0.06 |
| Reported low birth weight | 222 (38) | 3.25 (2.97–3.56) | <0.001 | 0.18 |
| **Anthropometry** | | | | |
| Weight (kg) | | | | |
| <1.5 | 213 (38) | 4.95 (4.13–5.93) | <0.001 | 0.09 |
| 1.5 to <2.5 | 174 (15) | 1.86(1.53–2.26) | <0.001 | |
| ≥2.5 | 229 (8.5) | Reference | | |
| Missing weight | 29 (67) | 10.7 (7.60–14.9) | <0.001 | |
| MUAC (cm) | | | | |
| <9.0 | 333 (25) | 3.57 (3.07–4.15) | <0.001 | 0.08 |
| 9 to 10 | 106 (12) | 1.73 (1.45–2.07) | <0.001 | |
| 10 to 11 | 96 (9.3) | 1.48 (1.24–1.77) | <0.001 | |
| ≥11 | 75 (7.1) | Reference | | |
| Missing MUAC | 35 (28) | 3.89 (3.02–5.00) | <0.001 | |
| **Clinical features** | | | | |
| Axillary temperature | | | | |
| <36˚C | 390 (29) | 3.13 (2.82–3.47) | <0.001 | 0.34 |
| 36 to 37.5˚C | 159 (9.3) | Reference | | |
| >37.5˚C | 84 (6.3) | 0.77 (0.67–0.88) | <0.001 | |
| Missing temperature | 12 (35) | 3.78 (2.65–5.39) | <0.001 | |
| Respiratory rate/min | | | | |
| Bradypnoea | 108 (57) | 6.09 (4.98–7.46 | <0.001 | 0.71 |
| Normal | 329 (12) | Reference | | |
| Tachypnoea | 197 (13) | 1.07 (0.90–1.27) | 0.45 | |
| Missing | 11 (11) | 0.91 (0.51–1.65) | 0.76 | |
| Heart rate/min | | | | |
| Bradycardia | 74 (47) | 4.05 (3.21–5.11) | <0.001 | 0.50 |
| Normal | 403 (14) | Reference | | |
| Tachycardia | 163 (12) | 0.88 (0.73–1.05) | 0.15 | |
| Missing | 5 (23) | 1.68 (0.72–3.93) | 0.23 | |
| Hypoxia (SaO2 <90%) | 309 (33) | 3.91 (3.58–4.26) | <0.001 | 0.48 |
| Lower chest wall indrawing | 448 (22) | 2.86 (2.60–3.15) | <0.001 | 0.41 |

For peer review only



For peer review only - http://bmjopen.bmj.com/site/about/guidelines.xhtml

| | | | | |
|---|---|---|---|---|
| Wheeze | 0 | - | | |
| Stridor | 6 (32) | 1.48 (0.89–2.47) | 0.13 | 0.23 |
| Breathing difficulty | 481 (22) | 3.62 (3.26–4.02) | <0.001 | 0.28 |
| Cyanosis | 98 (47) | 4.01 (3.55–4.53) | <0.001 | 0.14 |
| Capillary refill >2 seconds | 61 (58) | 5.61 (4.41–7.14) | <0.001 | 0.54 |
| Temperature gradient | 98 (38) | 2.85 (2.51–3.23) | <0.001 | 0.36 |
| Weak pulse | 108 (69) | 5.85 (5.21–6.57) | <0.001 | 0.71 |
| Lethargy | 64 (20) | 1.20 (1.04–1.40) | 0.02 | 0.34 |
| Impaired consciousness | 140 (56) | 5.43 (4.91–6.00) | <0.001 | 0.42 |
| Bulging fontanel | 6 (19) | 1.34 (0.90–1.99) | 0.15 | 0.08 |
| Stiff neck | 4 (40) | 2.08 (1.30–3.32) | 0.002 | 0.17 |
| Convulsions | 16 (8.1) | 0.75 (0.61–0.93) | 0.01 | 0.53 |
| Sunken eyes | 5 (11) | 1.04 (0.69–1.56) | 0.85 | 0.52 |
| Reduced skin turgor | 21 (22) | 1.18 (0.91–1.52) | 0.22 | 0.56 |
| Pallor | 72 (33) | 2.47 (2.15–2.83) | <0.001 | 0.63 |
| **Laboratory features** | | | | |
| Meningitis | 8 (24) | 8.06 (3.96–16.4) | <0.001 | 0.13 |
| Anaemia (haemoglobin <11 g/dl) | 50 (11) | 0.67 (0.51–0.90) | 0.007 | 0.51 |
| HIV antibody positive | 29 (20) | 1.39 (1.13–1.71) | 0.002 | 0.28 |
| Malaria slide positive | 0 | - | | |
| Bacteraemia | 63 (37) | 2.92 (2.10–4.06) | <0.001 | 0.13 |
| Blood glucose (mmols/L) | | | | |
| <2.6 | 137 (16) | 1.18 (1.04–1.33) | 0.009 | |
| 2.6 to 7.0 | 229 (12) | Reference | | 0.42 |
| >7.0 | 71 (31) | 2.85 (2.47–3.29) | <0.001 | |
| Missing blood glucose | 208 (15) | 1.18 (1.06–1.32) | 0.002 | |
| White blood cells ($10^{12}$ cells/L) | | | | |
| <4 | 14 (26) | 2.43 (1.47–4.03) | 0.001 | |
| 4-20 | 362 (11) | Reference | | 0.70 |
| >20 | 210 (30) | 2.97 (2.52–3.50) | <0.001 | |
| Missing | 59 (13) | 1.19 (0.91–1.57) | 0.20 | |
| Platelets ($10^{9}$ cells/L) | | | | |
| <150 | 139 (24) | 1.89 (1.57–2.27) | <0.001 | |
| ≥150 | 447 (13) | Reference | | 0.10 |
| Missing | 59 (13) | 1.01 (0.77–1.32) | 0.95 | |
| SHR: sub-distribution hazard ratios; the SHR are from the Fine and Gray's proportional sub-hazards model. | | | | |



For peer review only - http://bmjopen.bmj.com/site/about/guidelines.xhtml

Table S4. **Univariate analysis of admission features associated with post-discharge deaths.**

| N=3625 | Deaths (N=208) | Crude HR | P-value | Scaled Schoenfeld residuals P-value |
|---|---|---|---|---|
| Demographics | | | | |
| Age in days | | | | |
| 0 to 2 | 124 (5.6) | 0.98 (0.67–1.44) | 0.92 | 0.10 |
| 3 to 7 | 15 (3.7) | 0.63 (0.34–1.16) | 0.14 | |
| 8 to 28 | 36 (6.6) | 1.17 (0.73–1.87) | 0.52 | |
| >28 | 33 (5.5) | Reference | | |
| Sex (female) | 89 (5.5) | 0.98 (0.74–1.28) | 0.86 | 0.79 |
| Reported born premature | 58 (8.0) | 1.79 (1.32–2.44) | <0.001 | 0.07 |
| Reported low birth weight | 33 (9.3) | 1.99 (1.37–2.90) | <0.001 | 0.13 |
| Length of hospitalization (days)-log transformed | - | 1.96 (1.68–2.27) | <0.001 | 0.38 |
| Discharged over weekend | | | | |
| No | 173 (5.7) | Reference | | |
| Yes | 35 (4.8) | 0.85 (0.59–1.23) | 0.39 | 0.16 |
| Type of discharge | | | | |
| Normal discharge | 180 (4.9) | Reference | | 0.75 |
| Absconded | 5 (12) | 2.60 (1.07–6.33) | 0.04 | |
| Transferred/referred | 23 (44) | 11.8 (7.64–18.2) | <0.001 | |
| **Anthropometry** | | | | |
| Weight (kg) | | | | |
| <1.5 | 30 (8.5) | 2.49 (1.65–3.77) | <0.001 | 0.17 |
| 1.5 to <2.5 | 87 (9.2) | 2.64 (1.97–3.54) | <0.001 | |
| ≥2.5 | 91 (3.7) | Reference | | |
| Missing weight | 0 | - | | |
| MUAC (cm) | | | | |
| <9.0 | 88 (8.8) | 4.05 (2.56–6.41) | <0.001 | 0.17 |
| 9 to 10 | 44 (5.8) | 2.56 (1.55–4.24) | <0.001 | |
| 10 to 11 | 42 (4.5) | 1.92 (1.15–3.18) | 0.01 | |
| ≥11 | 23 (2.4) | Reference | | |
| Missing MUAC | 11 (12) | 5.83 (2.84–12.0) | <0.001 | |
| **Clinical features** | | | | |
| Axillary temperature | | | | |
| <36˚C | 78 (8.1) | 1.45 (1.07–1.96) | 0.02 | 0.80 |
| 36 to 37.5˚C | 88 (5.7) | Reference | | |
| >37.5˚C | 41 (3.3) | 0.57 (0.40–0.83) | 0.003 | |
| Missing temperature | 1 (5.0) | 0.99 (0.14–7.12) | 0.99 | |
| Respiratory rate/min | | | | |
| Bradypnoea | 7 (8.8) | 1.92 (0.90–4.13) | 0.09 | 0.30 |
| Normal | 108 (4.7) | Reference | | |
| Tachypnoea | 87 (6.8) | 1.44 (1.09–1.92) | 0.01 | |
| Missing | 6 (7.2) | 1.65 (0.72–3.76) | 0.23 | |
| Heart rate/min | | | | |
| Bradycardia | 9 (11) | 2.10 (1.07–4.13) | 0.03 | 0.73 |
| Normal | 137 (5.5) | Reference | | |
| Tachycardia | 62 (5.3) | 0.97 (0.72–1.31) | 0.85 | |



For peer review only - http://bmjopen.bmj.com/site/about/guidelines.xhtml

| | | | | |
|---|---|---|---|---|
| Missing | 0 | - | | |
| Hypoxia (SaO2 <90%) | 51 (8.2) | 1.68 (1.23–2.31) | 0.001 | 0.54 |
| Lower chest wall indrawing | 108 (6.8) | 1.54 (1.17–2.02) | 0.002 | 0.18 |
| Wheeze | 2 (4.4) | 0.77 (0.19–3.10) | 0.71 | 0.20 |
| Stridor | 0 | - | | |
| Breathing difficulty | 109 (6.5) | 1.40 (1.07–1.85) | 0.02 | 0.26 |
| Cyanosis | 7 (6.3) | 1.14 (0.54–2.43) | 0.73 | 0.17 |
| Capillary refill ≥2 seconds | 4 (9.4) | 1.81 (0.67–4.87) | 0.24 | 0.40 |
| Temperature gradient | 12 (7.5) | 1.44 (0.80–2.57) | 0.23 | 0.22 |
| Weak pulse | 7 (15) | 3.10 (1.46–6.59) | 0.003 | 0.38 |
| Lethargy | 14 (5.8) | 1.06 (0.63–1.80) | 0.82 | 0.19 |
| Impaired consciousness | 6 (5.5) | 0.98 (0.44–2.21) | 0.96 | 0.50 |
| Bulging fontanel | 4 (15) | 3.04 (1.13–8.18) | 0.03 | 0.06 |
| Stiff neck | 1 (17) | 2.84 (0.40–20.2) | 0.30 | 0.15 |
| Convulsions | 9 (5.0) | 0.88 (0.46–1.75) | 0.75 | 0.31 |
| Sunken eyes | 6 (15) | 3.31 (1.47–7.45) | 0.004 | 0.20 |
| Reduced skin turgor | 8 (11) | 2.19 (1.08–4.43) | 0.03 | 0.20 |
| Pallor | 15 (10) | 2.09 (1.23–3.53) | 0.006 | 0.23 |
| **Laboratory features** | | | | |
| Meningitis | 4 (16) | 3.98 (1.45–10.9) | 0.007 | 0.13 |
| Anaemia (haemoglobin <11 g/dl) | 26 (6.2) | 1.19 (0.79–1.80) | 0.41 | 0.68 |
| HIV antibody positive | 7 (6.2) | 1.17 (0.55–2.49) | 0.69 | 0.76 |
| Malaria slide positive | 0 | - | | |
| Bacteraemia | 10 (9.4) | 1.02 (0.50–2.06) | 0.96 | 0.28 |
| Blood glucose (mmols/L) | | | | |
| <2.6 | 51 (6.9) | 1.31 (0.92–1.85) | 013 | 0.25 |
| 2.6 to 7.0 | 86 (5.3) | Reference | | |
| >7.0 | 10 (6.3) | 1.21 (0.63–2.32) | 0.57 | |
| Missing blood glucose | 61 (5.0) | 0.95 (0.68–1.32) | 0.75 | |
| White blood cells ($10^{12}$ cells/L) | | | | |
| <4 | 1 (2.5) | 0.45 (0.06–3.20) | 0.42 | 0.79 |
| 4-20 | 157 (5.5) | Reference | | |
| >20 | 29 (6.1) | 1.10 (0.74–1.64) | 0.63 | |
| Missing | 21 (5.4) | 0.99 (0.63–1.56) | 0.97 | |
| Platelets ($10^{9}$ cells/L) | | | | |
| <150 | 38 (8.6) | 1.69 (1.18–2.41) | 0.004 | 0.70 |
| ≥150 | 149 (5.1) | Reference | | |
| Missing | 21 (5.4) | 1.08 (0.68–1.70) | 0.75 | |
| HR: hazard ratios; the HR are from the Cox proportional hazards model. | | | | |

For peer review only

Table S5 **Pathogens isolated from blood and CSF culture of young infants resident of KHDSS during inpatient period**.

| **Blood culture Isolates** | | **CSF culture isolates** | |
|---|---|---|---|
| **Pathogen full names (N=178)** | No. (%) | **Pathogen full names (N=24)** | No. (%) |
| *Klebsiella pneumoniae* | 53 (30) | *Escherichia coli* | 6 (25) |
| *Escherichia coli* | 25 (14) | Group B Streptococcus | 6 (25) |
| *Staphylococcus aureus* | 22 (12) | *Klebsiella pneumoniae* | 3 (13) |
| Group B Streptococcus | 19 (11) | *Streptococcus pneumoniae* | 3 (13) |
| Non-typhoidal Salmonella species | 9 (5.1) | *Enterobacter cloacae* | 3 (13) |
| *Enterobacter cloacae* | 8 (4.5) | Non-typhoidal Salmonella species | 2 (8.2) |
| *Pseudomonas aeruginosa* | 6 (3.4) | *Acinetobacter lwoffi* | 1 (4.2) |
| *Streptococcus pneumoniae* | 5 (2.8) | | |
| *Streptococcus pyogenes* | 3 (1.7) | | |
| Acinetobacter species | 3 (1.7) | | |
| *Aeromonas hydrophila* | 3 (1.7) | | |
| Group A Streptococcus | 3 (1.7) | | |
| *Serratia marcescens* | 2 (1.1) | | |
| *Acinetobacter calcoaceticus/baumannii* | 2 (1.1) | | |
| *Acinetobacter lwoffi* | 1 (0.6) | | |
| *Aeromonas sobria* | 1 (0.6) | | |
| *Chryseobacterium indologenes* | 1 (0.6) | | |
| *Enterobacter aerogenes* | 1 (0.6) | | |
| Enterococci species | 1 (0.6) | | |
| *Haemophilus influenzae* | 1 (0.6) | | |
| *Proteus mirabilis* | 1 (0.6) | | |

CSF; cerebrospinal fluid, Out of the 33 Meningitis cases, only the 24 presented had positive CSF culture.



For peer review only - http://bmjopen.bmj.com/site/about/guidelines.xhtml

Table S6. **Annual admissions and case fatality ratios (CFR).**

| Admissions/Year | 2009 | 2010 | 2011 | 2012 | 2013 | 2014 | 2015 | 2016 | 2017 | 2018 | 2019 |
|---|---|---|---|---|---|---|---|---|---|---|---|
| <60 days | 407 | 455 | 425 | 418 | 319 | 424 | 429 | 472 | 275 | 323 | 474 |
| 2 to 59 months | 1734 | 1462 | 1222 | 1177 | 682 | 1050 | 1017 | 787 | 432 | 920 | 918 |
| Proportion of YI admissions | 19% | 24% | 26% | 28% | 32% | 29% | 30% | 37% | 39% | 26% | 34% |
| YI inpatient deaths | 46 | 66 | 72 | 64 | 47 | 59 | 71 | 65 | 51 | 42 | 62 |
| YI inpatient CFR | 11% | 15% | 17% | 15% | 15% | 14% | 17% | 14% | 19% | 13% | 13% |
| YI post-discharge 1-year deaths | 19 | 24 | 17 | 18 | 25 | 15 | 21 | 19 | 10 | 15 | 25 |
| YI Post-discharge 1- year CFR | 5.4% | 6.4% | 5.1% | 5.3% | 9.5% | 4.3% | 6.2% | 4.8% | 4.7% | 5.6% | 6.3% |

Table S7. **Estimated causes of post-discharge deaths during readmission at KCH (67 deaths**).

| Index admission diagnosis (N=67) | No. (%) | Causes of post-discharge deaths (N=67) | No. (%) |
|---|---|---|---|
| Neonatal sepsis | 15 (22) | Neonatal sepsis | 16 (24) |
| Preterm complications | 15 (22) | Preterm complications | 15 (22) |
| Heart disease-Congenital | 9 (13) | Heart disease-Congenital | 10 (15) |
| Neonatal jaundice | 5 (7.5) | Neonatal jaundice | 5 (7.4) |
| Meningitis | 4 (6.0) | Meningitis | 5 (7.4) |
| Birth asphyxia | 4 (6.0) | Birth asphyxia | 5 (7.4) |
| Lower respiratory tract infection | 4 (6.0) | Lower respiratory tract infection | 4 (6.0) |
| Encephalopathy - unknown | 0 | Encephalopathy - unknown | 1 (1.5) |
| Hydrocephalus | 1 (1.5) | Hydrocephalus | 1 (1.5) |
| Malnutrition | 1 (1.5) | None specified | 5 (7.4) |
| None specified | 9 (13) | | |

Index admission diagnosis and causes of death were assigned by treating clinician.



For peer review only - http://bmjopen.bmj.com/site/about/guidelines.xhtml

Table S8. **Univariate analysis of admission features associated with inpatient deaths among babies born at KCH only.**

| **N=1,358** | **Deaths (N=174) N (%)** | **Crude SHR** | **P-value** | **Scaled Schoenfeld residuals P-value** |
|---|---|---|---|---|
| Demographics | | | | |
| Age in days | | | | |
| 0 to 2 | 151 (15) | 2.48 (1.19–5.16) | 0.02 | 0.26 |
| 3 to 7 | 8 (7.0) | 1.22 (0.45–3.30) | 0.69 | |
| 8 to 28 | 8 (6.8) | 1.21 (0.46–3.23) | 0.70 | |
| >28 | 7 (5.5) | Reference | | |
| Sex (female) | 84 (14) | 1.11 (0.83–1.48) | 0.50 | 0.31 |
| Born premature | 101 (21) | 2.65 (1.97–3.56) | <0.001 | 0.10 |
| Born low birth weight | 107 (21) | 2.74 (2.03–3.71) | <0.001 | 0.44 |
| **Anthropometry** | | | | |
| Weight z score at birth | | | | |
| <-2.0 | 32 (17) | 1.49 (1.02–2.17) | 0.04 | 0.12 |
| ≥-2.0 | 142 (12) | Reference | | |
| MUAC (cm) | | | | |
| <9.0 | 97 (23) | 3.14 (2.05–4.83) | <0.001 | 0.17 |
| 9 to 10 | 26 (9.9) | 1.26 (0.74–2.17) | 0.40 | |
| 10 to 11 | 25 (7.3) | 0.91 (0.53–1.57) | 0.73 | |
| ≥11 | 26 (7.9) | Reference | | |
| Missing MUAC | 0 | - | | |
| **Clinical features** | | | | |
| Axillary temperature | | | | |
| <36°C | 113 (26) | 3.02 (2.13–4.27) | <0.001 | 0.30 |
| 36 to 37.5°C | 41 (9.2) | Reference | | |
| >37.5°C | 18 (3.9) | 0.42 (0.24–0.72) | 0.002 | |
| Missing temperature | 2 (50) | 6.90 (1.84–25.8) | 0.004 | |
| Respiratory rate/min | | | | |
| Bradypnoea | 32 (52) | 6.44 (4.39–9.45) | <0.001 | 0.17 |
| Normal | 91 (10) | Reference | | |
| Tachypnoea | 50 (12) | 1.21 (0.86–1.69) | 0.28 | |
| Missing | 1 (5.3) | 0.49 (0.07–3.37) | 0.47 | |
| Heart rate/min | | | | |
| Bradycardia | 21 (43) | 4.76 (3.06–7.43) | <0.001 | 0.81 |
| Normal | 98 (10) | Reference | | |
| Tachycardia | 55 (15) | 1.48 (1.07–2.05) | 0.02 | |



For peer review only - http://bmjopen.bmj.com/site/about/guidelines.xhtml

| | | | | |
|---|---|---|---|---|
| Missing | 0 | - | | |
| Hypoxia (SaO2 <90%) | 79 (29) | 3.62 (2.71–4.83) | <0.001 | 0.81 |
| Lower chest wall indrawing | 125 (21) | 3.57 (2.57–4.95) | <0.001 | 0.47 |
| Wheeze | 0 | - | | |
| Stridor | 5 (63) | 6.96 (3.09–15.7) | <0.001 | 0.60 |
| Breathing difficulty | 481 (22) | 5.40 (3.69–7.88) | <0.001 | 0.18 |
| Cyanosis | 29 (48) | 5.15 (3.54–7.50) | <0.001 | 0.18 |
| Capillary refill >2 seconds | 14 (64) | 7.22 (4.43–11.7) | <0.001 | 0.58 |
| Temperature gradient | 26 (30) | 2.69 (1.81–4.01) | <0.001 | 0.49 |
| Weak pulse | 25 (58) | 6.52 (4.44–9.56) | <0.001 | 0.77 |
| Lethargy | 17 (16) | 1.29 (0.79–2.10) | 0.31 | 0.56 |
| Impaired consciousness | 48 (52) | 6.75 (4.93–9.24) | <0.001 | 0.13 |
| Bulging fontanel | 1 (25) | 2.03 (0.31–13.2) | 0.46 | 0.59 |
| Stiff neck | 1 (5.3) | - | | 0.31 |
| Convulsions | 1 (2.9) | 0.21 (0.03–1.48) | 0.12 | 0.21 |
| Sunken eyes | 1 (17) | 1.35 (0.19–9.77) | 0.77 | 0.57 |
| Reduced skin turgor | 1 (5.9) | 0.44 (0.06–3.19) | 0.42 | 0.56 |
| Pallor | 21 (42) | 4.00 (2.61–6.12) | <0.001 | 0.59 |
| **Laboratory features** | | | | |
| Meningitis | 2 (29) | 9.95 (2.37–41.8) | 0.002 | 0.12 |
| Haemoglobin <11 g/dl | 11 (13) | 0.92 (0.51–1.66) | 0.78 | 0.25 |
| HIV antibody positive | 8 (21) | 1.68 (0.84–3.33) | 0.14 | 0.14 |
| Malaria slide positive | 0 | - | | |
| Bacteraemia | 14 (33) | 3.00 (1.48–5.95) | 0.002 | 0.20 |
| Blood glucose (mmols/l) | | | | |
| <2.6 | 33 (12) | 1.02 (0.67–1.56) | 0.91 | |
| 2.6 to 7.0 | 58 (12) | Reference | | 0.95 |
| >7.0 | 11 (21) | 1.88 (1.00–3.54) | 0.05 | |
| Missing blood glucose | 72 (13) | 1.11 (0.79–1.56) | 0.53 | |
| White blood cells ($10^{12}$ cells/L) | | | | |
| <4 | 3 (33) | 3.52 (1.25–9.91) | 0.02 | |
| 4-20 | 100 (10) | Reference | | 0.08 |
| >20 | 59 (28) | 3.10 (2.27–4.24) | <0.001 | |
| Missing | 12 (8.2) | 0.82 (0.45–1.48) | 0.50 | |
| Platelets ($10^9$ cells/L) | | | | |
| <150 | 35 (20) | 1.69 (1.18–2.44) | 0.005 | |
| ≥150 | 127 (12) | Reference | | 0.50 |
| Missing | 12 (8.2) | 0.66 (0.37–1.19) | 0.17 | |

SHR; sub-distribution hazard ratios; the SHR are from the Fine and Gray's proportional sub-hazards model.



For peer review only - http://bmjopen.bmj.com/site/about/guidelines.xhtml

Table S9. **Multivariable regression analysis of factors associated with inpatient and post-discharge mortality among children born at KCH only.**

| | Inpatient analysis | | Post-discharge analysis | |
|---|---|---|---|---|
| | Adjusted SHR* | P-value | Adjusted HR | P-value |
| **Demographics** | | | | |
| Age in days | | | | |
| 0 to 2 | 3.03 (1.33–6.94) | 0.009 | ¶ | |
| 3 to 7 | 4.08 (1.48–11.3) | 0.007 | ¶ | |
| 8 to 28 | 2.57 (0.90–7.29) | 0.08 | ¶ | |
| >28 | Reference | | | |
| **Anthropometry** | | | | |
| Low birth weight | 1.55 (1.10–2.20) | 0.01 | 2.76 (1.30–5.82) | 0.008 |
| **Clinical features** | | | | |
| Axillary temperature | | | | |
| <36˚C | 1.49 (0.97–2.28) | 0.07 | 1.44 (0.71–2.95) | 0.31 |
| 36 to 37.5˚C | Reference | | Reference | |
| >37.5˚C | 0.67 (0.39–1.13) | 0.13 | 0.22 (0.06–0.78) | 0.02 |
| Missing temperature | 3.37 (0.85–13.4) | 0.09 | 0.48 (0.09–2.64) | 0.40 |
| Respiratory rate/min | | | | |
| Bradypnoea | 2.22 (1.36–3.63) | 0.001 | ¶ | |
| Normal | Reference | | | |
| Tachypnoea | 0.78 (0.55–1.11) | 0.17 | ¶ | |
| Missing | - | | | |
| Heart rate/min | | | | |
| Bradycardia | 1.88 (1.14–3.12) | 0.01 | 3.56 (1.14–11.2) | 0.03 |
| Normal | Reference | | Reference | |
| Tachycardia | 1.57 (1.11–2.21) | 0.01 | 1.54 (0.77–3.07) | 0.22 |
| Missing | 0.25 (0.02–3.00) | 0.28 | - | |
| Hypoxia (SaO2 <90%) | 1.60 (1.14–2.24) | 0.006 | ¶ | |
| Lower chest wall indrawing | 1.42 (0.91–2.22) | 0.12 | ¶ | |
| Stridor | 3.74 (1.87–7.49) | <0.001 | ¶ | |
| Breathing difficulty | 2.13 (1.25–3.64) | 0.005 | ¶ | |
| Capillary refill >2 seconds | 1.94 (1.06–3.56) | 0.03 | ¶ | |
| Weak pulse | 2.15 (1.27–3.65) | 0.004 | 1.60 (0.24–10.5) | 0.63 |
| Pallor | 2.36 (1.46–3.83) | <0.001 | ¶ | |
| **Laboratory features** | | | | |
| Bacteraemia | 2.50 (1.20–5.22) | 0.02 | 0.21 (0.03–1.81) | 0.16 |
| Model performance | | | | |
| AUC (95% CI) | 0.85 (0.82–0.88) | | 0.79 (0.72–0.85) | |

SHR; sub-distribution hazard ratios; *the SHR are from the Fine and Gray's proportional subhazards model, HR-Hazard ratio from the Proportional Cox regression model, ¶; variables not selected for inclusion in the multivariable model, AUC; area under receiver operating characteristics.



For peer review only - http://bmjopen.bmj.com/site/about/guidelines.xhtml

Figure S1. **A: Monthly all young infant admissions, B: all admissions (<60 months old), C: all admissions (<60 months old) case fatality and D: 2–59 months old case fatality before and after July 2013.**

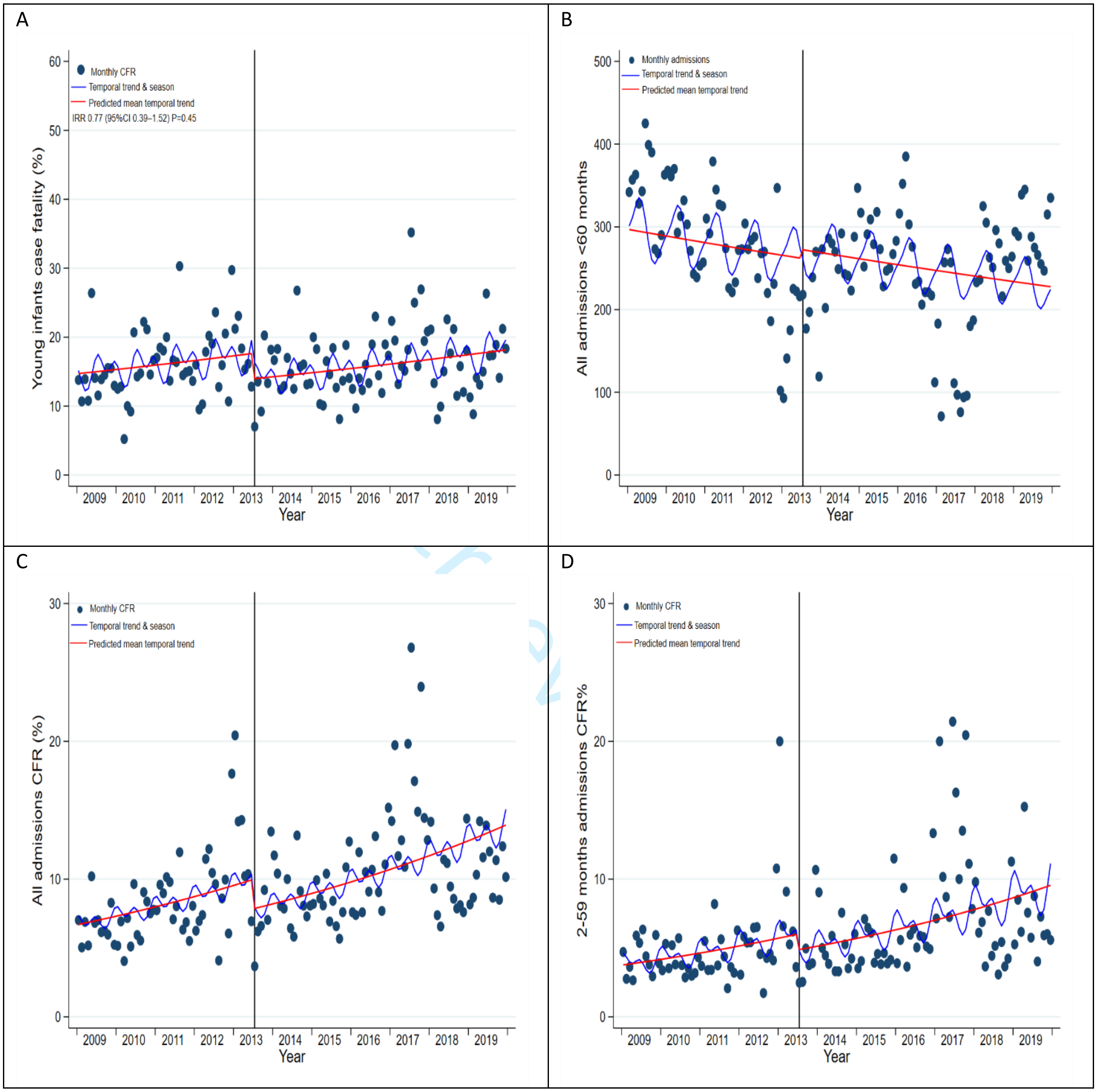

# Trends in inpatient and post-discharge mortality among young infants admitted to Kilifi County Hospital, Kenya, a retrospective cohort study.

| | |
|---|---|
| Journal: | *BMJ Open* |
| Manuscript ID | bmjopen-2022-067482.R1 |
| Article Type: | Original research |
| Date Submitted by the Author: | 30-Nov-2022 |
| Complete List of Authors: | Talbert, Alison; KEMRI-Wellcome Trust Research Programme<br>Ngari, Moses; KEMRI-Wellcome Trust Research Programme<br>Obiero, Christina; KEMRI-Wellcome Trust Research Programme; University of Amsterdam, Department of Global Health<br>Nyaguara, A.; KEMRI-Wellcome Trust Research Programme<br>Mwangome, MK; KEMRI-Wellcome Trust Research Programme<br>Mturi, Neema; KEMRI-Wellcome Trust Research Programme<br>Ouma, Nelson; KEMRI-Wellcome Trust Research Programme<br>Otiende, M.; KEMRI-Wellcome Trust Research Programme<br>Berkley, James ; KEMRI-Wellcome Trust Research Programme; University of Oxford, Centre for Tropical Medicine & Global Health |
| <b>Primary Subject Heading</b>: | Paediatrics |
| Secondary Subject Heading: | Epidemiology |
| Keywords: | Epidemiology < TROPICAL MEDICINE, PAEDIATRICS, NEONATOLOGY, EPIDEMIOLOGY |
| | |

SCHOLARONE™ Manuscripts

*I, the Submitting Author has the right to grant and does grant on behalf of all authors of the Work (as defined in the below author licence), an exclusive licence and/or a non-exclusive licence for contributions from authors who are: i) UK Crown employees; ii) where BMJ has agreed a CC-BY licence shall apply, and/or iii) in accordance with the terms applicable for US Federal Government officers or employees acting as part of their official duties; on a worldwide, perpetual, irrevocable, royalty-free basis to BMJ Publishing Group Ltd ("BMJ") its licensees and where the relevant Journal is co-owned by BMJ to the co-owners of the Journal, to publish the Work in this journal and any other BMJ products and to exploit all rights, as set out in our licence.*

*The Submitting Author accepts and understands that any supply made under these terms is made by BMJ to the Submitting Author unless you are acting as an employee on behalf of your employer or a postgraduate student of an affiliated institution which is paying any applicable article publishing charge ("APC") for Open Access articles. Where the Submitting Author wishes to make the Work available on an Open Access basis (and intends to pay the relevant APC), the terms of reuse of such Open Access shall be governed by a Creative Commons licence – details of these licences and which Creative Commons licence will apply to this Work are set out in our licence referred to above.*

*Other than as permitted in any relevant BMJ Author's Self Archiving Policies, I confirm this Work has not been accepted for publication elsewhere, is not being considered for publication elsewhere and does not duplicate material already published. I confirm all authors consent to publication of this Work and authorise the granting of this licence.*

For peer review only

# Trends in inpatient and post-discharge mortality among young infants admitted to Kilifi County Hospital Kenya, a retrospective cohort study.

Alison Talbert MRCP [1], Moses Ngari PhD[1,2], Christina Obiero MPH[1,3], Amek Nyaguara PhD[1], Martha Mwangome PhD[1,2], Neema Mturi MRCP[1], Nelson Ouma BSc[1], Mark Otiende MSc[1], and James A Berkley FRCPCH[1,2,4]

Institutions of affiliation

1. KEMRI/Wellcome Trust Research Programme, Centre for Geographic Medicine Research -Coast, PO Box 230 - 80108, Kilifi, Kenya.
2. The Childhood Acute Illness & Nutrition Network (CHAIN), Nairobi, Kenya.
3. Department of Global Health, University of Amsterdam, Amsterdam, The Netherlands
4. Center for Tropical Medicine & Global Health, University of Oxford, Oxford, UK.

Corresponding author: Alison Talbert Email: ATalbert@kemri-wellcome.org
P.O Box 230,Kilifi, 80108 Kenya Phone: +254 729 218993

Word count 3883

For peer review only



For peer review only - http://bmjopen.bmj.com/site/about/guidelines.xhtml

## Abstract

**Objectives:** to describe admission trends and estimate inpatient and post-discharge mortality and its associated exposures, among young infants (YI) admitted to a county hospital in Kenya

**Design:** retrospective cohort study

**Setting:** secondary level hospital

**Participants:** YI aged less than 60 days admitted to hospital January 2009 to December 2019: 12,271 admissions in 11,877 individuals. YI who were resident within a health and demographic surveillance system (KHDSS): n= 3,625 with 4,421 admissions were followed up for 1 year after discharge.

**Primary and secondary outcome measures:** Inpatient and 1-year post-discharge mortality, the latter in KHDSS residents.

**Results:** Of 12,271 YI admissions, 4,421 (36%) were KHDSS-resident. Neonatal sepsis, preterm complications and birth asphyxia accounted for 83% of admissions. The proportion of YI among under-fives admissions increased from 19% in 2009 to 34% in 2019 ($P_{trend}$ =0·02). Inpatient case fatality was 16%, with 66% of deaths occurring within 48 hours of admission. The introduction of free maternity care in 2013 was not associated with a change in admissions or inpatient mortality among YI. During 1-year post-discharge, 208/3625 (5·7%) YI died, 64·3 (95%CI 56·2–73·7) per 1,000 infant-years. 49% of post-discharge deaths occurred within one month of discharge, and 49% of post-discharge deaths occurred at home. Both inpatient and post-discharge deaths were associated with low admission weight. Inpatient mortality was




For peer review only - http://bmjopen.bmj.com/site/about/guidelines.xhtml

associated with clinical signs of disease severity, while post-discharge mortality was associated with length of hospitalization, leaving against advice and referral to a specialized hospital.

**Conclusions:** YIs accounted for an increasing proportion of paediatric admissions and their overall mortality remains high. Post-discharge mortality accounts for a lower proportion of deaths but mortality rate is higher than among children aged 2-59 months. Services to address post-discharge mortality are needed and should focus on infants at higher risk.

## Key words

Young infant; neonatal; mortality; inpatient; post-discharge; Africa; Kenya

291 words



For peer review only - http://bmjopen.bmj.com/site/about/guidelines.xhtml

## Article summary

## Strengths and limitations of this study

- Large sample size with systematic data collection
- Linkage of hospital admissions to a well-established demographic surveillance system, with low loss to follow up.
- Lack of accurate gestational age estimation or birthweight of most participants.
- Data are from a single hospital and only the population covered by demographic surveillance



For peer review only - http://bmjopen.bmj.com/site/about/guidelines.xhtml

## Background

The United Nations Sustainable Development Goal 3 aims to ensure healthy living and promote wellbeing for all ages, with all countries aiming to reduce neonatal and under-five mortality to below 12 and 25 per 1,000 live births by 2030 respectively. In sub-Saharan Africa, child mortality has declined by ~58% in the last 30 years. However, the estimated neonatal and under-five mortality rates in sub-Saharan Africa remained high in 2019 (27 and 76 per 1,000 live births respectively) with a similar neonatal mortality rate of 27 per 1,000 live births in Kenya.[1] Combined neonatal and post-neonatal infant mortality accounts for over three quarters of all under-five deaths in Kenyan children.[2]

Young infants aged <60 days old (YI) comprise around half of hospital admissions in sub-Saharan Africa and continue to face high risk of in-hospital mortality and long-term neuro-disability.[3-6] Post-discharge mortality is emerging as a major problem in children in low- and middle-income countries (LMICs),[7] however, there are limited data among YI. A systematic review of paediatric post-discharge mortality in developing countries included 24 studies published up to July 2017 with 19 from Africa.[8] Four studies included YI. Although young age was reported as a risk factor of mortality, no studies specifically identified deaths among infants aged <60 days. We have previously demonstrated excess post-discharge mortality among all hospitalised children, suggesting that hospitalisation itself selects vulnerable children with a sustained increased risk of dying over the longer term.[7 9]



For peer review only - http://bmjopen.bmj.com/site/about/guidelines.xhtml

For peer review only

Better understanding of YI deaths occurring during hospitalisation and after discharge from hospital is vital for development and use of targeted interventions aimed at improving survival.

This analysis aimed to describe admission trends and measure inpatient and post-discharge mortality and its associated exposures, including the introduction of free maternity care, among YI admitted to Kilifi County Hospital (KCH), Kenya and followed up through the Kilifi Health and Demographic Surveillance System (KHDSS).

For peer review only



For peer review only - http://bmjopen.bmj.com/site/about/guidelines.xhtml

# Methods

*Study participants and design*

KCH is a secondary-level referral hospital situated in Kilifi County along the Kenyan coast. It serves a rural and peri-urban population. It has a maternity unit. with approximately 6,000 deliveries per year, a general paediatric ward with a newborn unit for babies aged less than 1 month, and a paediatric High Dependency Unit (HDU) that also admits YIs. The year 2009 was selected as a starting point, because a previous analysis of mortality among YI covered admissions from 1990 to 2008[10]. Free maternity care was introduced by the Kenyan government on 1st June 2013 and led to a marked increase in health facility births.[11]

The KHDSS, established in 2002, covers a population of 279,158 within an area of 900km$^2$ centred on KCH.[12] Census rounds visit each household every four months to ascertain vital status and migration in and out of the hospital catchment area.

We conducted a retrospective cohort study of YIs resident within the KHDSS who were admitted to KCH between January 1st, 2009, and December 31st, 2019. Children discharged alive and followed up in KHDSS census rounds until March 2021 were eligible for analyses of post-discharge mortality. During the study period, there were 9 health workers' strikes with the last nurses' strike lasting for 150 days (5th June to 2nd November 2017).[13] **Supplementary Table S1.** For comparison, we also examined admissions aged 60 days to 59 months during the same period.



For peer review only - http://bmjopen.bmj.com/site/about/guidelines.xhtml

*Procedures*

At admission, standardised medical history, and clinical examination, including anthropometric measurements were obtained by trained clinical staff. Blood samples were systematically taken for complete blood count, slide for malaria microscopy, and clinical chemistry, Human Immunodeficiency Virus (HIV) antibody test and blood culture at hospital admission, as described previously.[14] A lumbar puncture for cerebrospinal fluid (CSF) analysis was done at admission in infants in whom sepsis was suspected and deferred in those seriously ill or with other contraindications. Clinical and laboratory data were recorded in real time on a ward surveillance database linked to the KHDSS database. Empiric antibiotics were initiated according to national guidelines[15] with ampicillin/benzylpenicillin plus gentamicin as first-line intravenous therapy. Second-line and subsequent antimicrobial therapy was guided by blood culture results and clinical progress. Mechanical ventilation was not available at KCH.

*Statistical methods*

Study variables

Outcomes of interest were death in hospital and during 1 year after discharge. Exposures of interest were demographic, nutritional, clinical features, and haematological, biochemical, and microbiological findings at the time of admission. De-identified study data were deposited in the Harvard Dataverse depository.[16]



For peer review only - http://bmjopen.bmj.com/site/about/guidelines.xhtml

Weight at admission and mid-upper arm circumference (MUAC) were categorised as shown on **Table 1**. Because approximately 40% of the YI were underweight (<2·5kg), and 60% were aged ≤2 days at admission, YI's admission weights rather than anthropometric Z scores using WHO standards were reported. Furthermore, most YI who were born at home or in other hospitals and referred to KCH were missing gestational age estimates and birth weight to be able to estimate gestational age at birth using the INTERGROWTH 21st Newborn Size Standards (INSS).

Prematurity was defined as gestation age <37 weeks and LBW as birth weight <2500 grams for YIs born at KCH. Admission blood glucose was categorized into <2·6, 2·6 to 7·0 and ≥7·0 mmol/l representing low, normal and high levels respectively.[15] Missing data were not assumed to be missing at random. We, therefore, created categorical variables and added a missing category which was included in the regression analysis.

Demographic, anthropometric, and clinical data are presented as frequencies and proportions for categorical variables and means (standard deviation (sd)) or median (interquartile range (IQR)) for continuous variables depending on the underlying distribution. Proportions of missing data for each variable are shown on **Supplementary Table S2**.

Monthly admissions and case fatality were plotted against time (month of admission) to visually inspect the trend from 2009 to 2019 and the predicted trend line superimposed on the curves. We used the Augmented Dickey Fuller test (ADF test) to test if the time series were stationary (no trend or seasonal effects). We also presented annual absolute admissions, proportion of YI among all admissions <60 months and case fatality. Monthly admissions and case fatality were



For peer review only - http://bmjopen.bmj.com/site/about/guidelines.xhtml

tested for annual linear trend using an extension of the Wilcoxon rank-sum test of trend across ordered groups.[17]

We used interrupted time series analysis to estimate the level and trend changes before and after introduction of free maternity care (1st June 2013). We created a time month variable coded sequentially from January 2009 to December 2019 and a binary variable coded as 0 and 1 for admissions before and after June 2013 respectively to represent to represent introduction of free maternity care . We defined seasonal effect variable using month of the year modelled on harmonic terms using the Fourier code in STATA. To measure the effect of free maternity care, we used the negative binomial regression model because of presence of overdispersion in the trends and reported regression coefficients transformed into incidence rate ratios (IRR). All the negative binomial regression models included the following independent variables: the time month variable, the binary pre- and post- free maternity care variable and the seasonal effect variable.

Since YIs could be admitted more than once whilst <60 days old, we included multiple admissions using unique IDs and adjusted for clustering by individual with robust standard errors. To identify exposures associated with inpatient death, we treated being discharged alive as a competing event and fitted the proportional sub-distribution hazard model using the Fine-Gray competing risk model.[18] The measure of effect reported from the model was the sub-distribution hazard ratios (SHR) and their respective 95% confidence intervals (CI). To build the multivariable regression model, a backward stepwise approach was used where all the independent variables assessed in the univariate models were included in the model and only those with a P-value <0·1 retained in the final multivariable model.



For peer review only - http://bmjopen.bmj.com/site/about/guidelines.xhtml

For the post-discharge analysis, only data from those YI discharged alive and resident within the KHDSS were analysed. Time at risk was defined from date of discharge to 365 days later or censure at date of death or outmigration from the KHDSS. We performed a 'multiple discharges' analysis where YI with multiple admissions had their follow-up time reset at each successive discharge date. Exposures associated with post-discharge were assessed using a Gamma distribution shared frailty Cox proportional hazards regression model accounting for YI with multiple discharges. The proportional hazards assumption was assessed using the scaled Schoenfeld residuals test (**Supplementary Tables S3 and S4**). All exposures assessed in the univariate models were considered for inclusion in the multivariable Cox proportional hazards regression model using a backward stepwise approach similar to the inpatient analysis. Both the inpatient and post-discharge multivariable regression models' discrimination performance were assessed using bootstrapped area under receiver operating characteristic curves (AUC) replicated 1000 times.

As sensitivity analysis, we assessed the YI born at KCH and enrolled to the Kilifi Perinatal and Maternal Research Project (KIPMAT), which had collected comprehensive birth data including birth weight and gestational age (weeks).[19] We estimated their birthweight Z scores using the INTERGROWTH Newborn Size Standards (INSS) and ran the regression models replacing admission weight with birthweight Z score.[20]

Statistical significance was evaluated using 95% CI and a two-tailed *P*-value <0·05. Statistical analyses were conducted using STATA Version 17.0 (College Station, TX, USA).

*Study size*



For peer review only - http://bmjopen.bmj.com/site/about/guidelines.xhtml

We used all available eligible YI data from 2009 to 2019 (4,421 for inpatient and 3,625 for post-discharge analyses) regardless of sample size.

*Ethical considerations*

Written consent was provided by the caregivers of all the surveillance study participants. Ethical approval to conduct this analysis was granted by the Kenya Medical Research Institute (KEMRI) National Ethics Review Committee (SCC 2778).

*Patient and public involvement*

There was no patient and public involvement in the planning or execution of this retrospective cohort study.



For peer review only - http://bmjopen.bmj.com/site/about/guidelines.xhtml

## Results

*Baseline characteristics*

During the study period, there were 42,742 paediatric admissions to KCH, of which 12,271 (29%) admission events among 11,877 individuals were aged <60 days. Of the 12,271 YI admission events, 4,421 (36%) were resident in the KHDSS and included in the analysis (**Figure 1**). This comprised 4,272 individual YI: 4,131 with one admission, 133 two admissions and 8 three admissions within the first 60 days of life.

*KHDSS-resident admissions*

Among the 4,421 YI admission events among KHDSS residents, 2,731 (62%) were ≤2 days old and 1,900 (43%) were female. Reported prematurity and low birth weight were 1,019 (23%) and 581 (13%) respectively. Low weight (<2·5kg) was observed in 1694 YIs (38%) while 1342 (30%) had MUAC <9·0cm. Common presenting clinical signs were lower chest wall indrawing (46%) and breathing difficulty (49%). Thirty percent had fever, 31% had hypothermia and 30% tachycardia. Nine hundred and thirty-two YI (21%) had hypoxia (SaO2 <90%) at admission and 250 (5·7%) had impaired consciousness. Presenting signs at admission for all the YI stratified by KHDSS residence are shown on **Table 1**. Malaria was rare (n=4, 0·09%) whilst 142 (3·2%) and 170 (3·9%) YI were HIV antibody positive and had bacteraemia respectively. **Supplementary Table S3** lists the bacterial isolates that were presumed pathogens, led by *Klebsiella pneumoniae, Escherichia coli, Staphylococcus aureus* and Group B Streptococcus.



For peer review only - http://bmjopen.bmj.com/site/about/guidelines.xhtml

**Table 1. Study participants characteristics at admission.**

| | All young infant admissions (N=12,271) [a] | Young infant admissions KHDSS residents (N=4,421) | Young infant admissions non-KHDSS residents (N=7,850) | p-value |
|---|---|---|---|---|
| **Demographics** | | | | |
| Age in days | | | | |
| 0 to 2 | 7856 (64) | 2731 (62) | 5125 (65) | <0·001 |
| 3 to 7 | 1384 (11) | 468 (11) | 916 (12) | |
| 8 to 28 | 1506 (12) | 587 (13) | 919 (12) | |
| >28 | 1525 (12) | 635 (14) | 890 (11) | |
| Sex (female) | 5245 (43) | 1900 (43) | 3345 (43) | 0·70 |
| Reported born premature | 2970 (24) | 1019 (23) | 1951 (25) | 0·005 |
| Reported low birth weight | 1782 (15) | 581 (13) | 1201 (15) | <0·001 |
| Born at KCH | | | | |
| Yes | 6757 (55) | 2743 (62) | 4014 (51) | <0.001 |
| No | 5514 (45) | 1678 (38) | 3836 (49) | |
| Anthropometry | | | | |
| Weight (kg) | | | | |
| <1·5 | 1767 (14) | 566 (13) | 1201 (15) | <0·001 |
| 1·5 to <2·5 | 3211 (26) | 1128 (26) | 2083 (27) | |
| ≥2·5 | 7193 (59) | 2684 (61) | 4509 (57) | |
| Missing | 100 (0·8) | 43 (1·0) | 57 (0·7) | |
| MUAC (cm) | | | | |
| <9 | 3933 (32) | 1342 (30) | 2591 (33) | <0·001 |
| 9 to 10 | 2492 (20) | 862 (20) | 1630 (21) | |
| 10 to 11 | 2926 (24) | 1035 (23) | 1891 (24) | |
| ≥11 | 2622 (21) | 1056 (24) | 1566 (20) | |
| Missing | 298 (2·4) | 126 (2·9) | 172 (2·2) | |
| Clinical features | | | | |
| Axillary temperature | | | | |
| <36˚C | 3553 (29) | 1358 (31) | 2195 (28) | <0·001 |
| 36 to 37•5˚C | 4692 (38) | 1711 (39) | 2981 (38) | |
| >37·5˚C | 3948 (32) | 1318 (30) | 2630 (34) | |
| Respiratory rate/min [b] | | | | |
| Bradypnoea | 540 (4·4) | 188 (4·3) | 352 (4·5) | 0·56 |
| Normal | 7333 (60) | 2647 (60) | 4686 (60) | |
| Tachypnoea | 4158 (34) | 1490 (34) | 2668 (34) | |
| Missing | 240 (2·0) | 96 (2·2) | 144 (1·8) | |
| Heart rate/min [c] | | | | |
| Bradycardia | 396 (3·2) | 158 (3·6) | 238 (3·0) | 0·11 |
| Normal | 8162 (67) | 2910 (66) | 5252 (67) | |
| Tachycardia | 3667 (30) | 1331 (30) | 2336 (30) | |
| Missing | 46 (0·4) | 22 (0·5) | 24 (0·3) | |

| | | | | |
|---|---|---|---|---|
| Hypoxia[d] | 2668 (22) | 932 (21) | 1736 (22) | 0·19 |
| Lower chest wall indrawing | 5562 (45) | 2051 (46) | 3511 (45) | 0·13 |
| Wheeze | 112 (0·9) | 46 (1·0) | 66 (0·8) | 0·41 |
| Stridor | 62 (0·5) | 19 (0·4) | 43 (0·6) | 0·48 |
| Breathing difficulty | 5966 (49) | 2172 (49) | 3794 (48) | 0·44 |
| Cyanosis | 560 (4·6) | 210 (4·8) | 350 (4·5) | 0·54 |
| Capillary refill >2 seconds | 301 (2·6) | 105 (2·4) | 196 (2·5) | 0·81 |
| Temperature gradient | 710 (5·8) | 258 (5·8) | 452 (5·8) | 0·73 |
| Weak pulse | 463 (3·8) | 157 (3·6) | 306 (3·9) | 0·05 |
| Lethargy | 971 (7·9) | 325 (7·4) | 646 (8·2) | 0·15 |
| Impaired consciousness[e] | 792 (6·5) | 250 (5·7) | 542 (6·9) | 0·007 |
| Bulging fontanel | 111 (0·9) | 32 (0·7) | 79 (1·0) | 0·21 |
| Stiff neck | 48 (0·4) | 10 (0·2) | 38 (0·5) | 0·05 |
| Convulsions | 689 (5·6) | 197 (4·5) | 492 (6·3) | <0·001 |
| Sunken eyes | 134 (1·1) | 44 (1·0) | 90 (1·2) | 0·44 |
| Reduced skin turgor | 308 (2·5) | 97 (2·2) | 211 (2·7) | 0·19 |
| Pallor | 633 (5·2) | 221 (5·0) | 412 (5·3) | 0·55 |
| **Laboratory features** | | | | |
| Meningitis [f] | 98 (0·8) | 33 (0·8) | 65 (0·8) | 0·87 |
| Haemoglobin <11 g/dl)[g] | 1207 (9·8) | 476 (11) | 731 (9·3) | 0·02 |
| HIV antibody positive | 441 (3·6) | 142 (3·2) | 299 (3·8) | 0·11 |
| Malaria slide positive | 5 (0·04) | 4 (0·09) | 1 (0·01) | 0·02 |
| Bacteraemia | 590 (4·8) | 170 (3·9) | 420 (5·4) | <0·001 |
| White blood cells ($10^{12}$ cells/L)[h] | | | | |
| <4 | 134 (1·1) | 54 (1·2) | 80 (1·0) | <0·001 |
| 4–20 | 8738 (71) | 3228 (73) | 5510 (70) | |
| >20 | 2202 (18) | 690 (16) | 1512 (19) | |
| unavailable | 1197 (9·8) | 449 (10) | 748 (9·5) | |
| Platelets ($10^9$ cells/L)[i] | | | | |
| <150 cells/L | 1615 (13) | 586 (13) | 1029 (13) | 0·59 |
| ≥150 | 9455 (77) | 3387 (77) | 6068 (77) | |
| unavailable | 1201 (9·8) | 448 (10) | 753 (9·6) | |
| Blood glucose (mmols/L) | | | | |
| <2·6 | 2479 (20) | 882 (20) | 1597 (20) | 0·29 |
| 2·6 to 7·0 | 5086 (41) | 1875 (42) | 3211 (41) | |
| >7·0 | 688 (5·6) | 231 (5·2) | 457 (5·8) | |
| unavailable | 4018 (33) | 1433 (32) | 2585 (33) | |



For peer review only - http://bmjopen.bmj.com/site/about/guidelines.xhtml

[a]-Eligible admissions were young infants aged <60days admitted from 2009 to 2019, [b]- Tachypnoea: respiratory rate ≥60 breaths/min, Bradypnoea: respiratory rate <30 breaths/min, [c]-Tachycardia: heart rate>160 beats/min, Bradycardia: heart rate<100 beats/min, [d]-Hypoxia: oxygen saturation<90%,[e]-Impaired consciousness level if 'prostrate' or 'unconscious', [f] Meningitis: positive CSF culture, or positive CSF microscopy, or positive CSF antigen test, or elevated CSF WBC count (≥20 in young infants aged 0-28 days OR, ≥10 in young infants aged 29-59 days) PLUS a positive blood culture for a known pathogen, [g] Anaemia: haemoglobin <11 g/dl, [h] Normal values WBC 4-20 x $10^{12}$ cells/L, Leucopoenia WBC <4 x $10^{12}$ cells/L, Leucocytosis WBC >20 x $10^{12}$ cells/L, [I] Normal values Platelets ≥150x$10^9$ cells/L, Thrombocytopenia <150x$10^9$ cells/L, KHDSS: Kilifi Health and Demographic Surveillance System, MUAC: Mid-upper arm circumference.

*Admissions over time*

The annual number of admissions are shown in **Supplementary Table S4.** The overall proportion of YI among all admissions under 5 years old was 28% (95%CI 27–29%), increasing from 19% in 2009 to 34% in 2019 (test of linear trend P=0·02) **Figure 2**. **Figure 3A** shows the upward trend of absolute YI admissions and downward trends for 2 to 59-month-olds and all admissions <60 months old (all P-values for tests for stationarity <0·05). There was no significant difference in monthly YI admissions before introduction of free maternity care in June 2013 (monthly median [IQR] of 76 [66–96] admissions) and after June 2013 (monthly median [IQR] of 95 [78–125] admissions) season-adjusted IRR 1·06 (95%CI 0·54–2·09) P=0·86 (**Supplementary Figure S1A**). The mean monthly YI admissions on day of birth did not differ before and after June 2013; season-adjusted IRR 0·88 (95%CI 0·44 to 1·76), P=0·72. The proportion of YI admissions to total admissions aged <60 months before and after June 2013 were not different; season-adjusted IRR 1·02 (95%CI 0·28–3·71) P=0·97 **Figure 3D**. We found no significant difference in monthly absolute admissions (all admissions <60 months old), before and after June 2013; season-adjusted IRR 1·01 (95%CI 0·51–2·00) P=0·97 (**Supplementary Figure S1B)**.

*Inpatient deaths*



For peer review only - http://bmjopen.bmj.com/site/about/guidelines.xhtml

Overall, 1,914/11,877 (16%) of YI died in hospital. The risk of inpatient death was not significantly different between 645/4,272 (15%) KHDSS residents and 1,269/7,605 (17%) non-residents of KHDSS (age- and sex-adjusted SHR 0·93 (95%CI 0·85–1·02) P=0·12) (**Figure 1**). The annual YI inpatient case fatality ratio was stable (11% in 2009 and 13% in 2019. P-value for trend=0·80), **Figure 2**. Monthly inpatient case fatality for YI, 2 to 59 months old and all <60 months old children are shown in **Figure 3B.**

During the study period there were 3,119 inpatient deaths among admissions <60 months old admitted at KCH, with YI admissions accounting for 61% (95%CI 60–63%) of the deaths and no significant linear trend from 2009 to 2019 (trend P=0·29). The mean monthly YI inpatient case fatality was 16% (sd 0·86) and 16% (sd 1·23) before and after June 2013 respectively; season-adjusted IRR 0·77 (95%CI 0·39–1·52) P=0·45 **Figure 3C**. The mean monthly case fatality for all admissions aged <60 months and admissions 2–59 months old did not differ before June 2013 and after June 2013; season-adjusted IRR 0·79 (95%CI 0·39–1·58) P=0·50 and IRR 0·81 (95%CI 0·39–1·69) P=0·57 respectively **Supplementary Figure S1 C** and **D**.

Among the 4,421 KHDSS-resident YI admissions, median [IQR] time to death was 2 [1–4] days, while the survivors were admitted for 5 [3–8] days. A total of 423/645 (66%) deaths occurred within the first 48 hours following admission. Forty-one YI left against medical advice, and 55 were referred to other hospitals for further care.

*Admission diagnosis & case fatality ratio*



For peer review only - http://bmjopen.bmj.com/site/about/guidelines.xhtml

The commonest reasons for hospital admission were neonatal sepsis (47%), preterm complications (20%) and birth asphyxia (16%) accounting for 83% of all YI admissions (**Table 2**). The case fatality ratios for YI with respiratory distress syndrome, preterm complications and birth asphyxia were 52%, 29% and 28% respectively (**Table 2**).

| **Discharge diagnosis[a]** | **No. (%) Diagnosis assigned by clinician at discharge** | |
|---|---|---|
| | **All admissions (N=4421)** | **Inpatient Deaths (N=645)** |
| Neonatal sepsis | 2097 (47) | 201 (9·6) |
| Preterm complications | 889 (20) | 262 (29) |
| Birth asphyxia | 724 (16) | 201 (28) |
| Neonatal jaundice | 611 (14) | 56 (9·2) |
| Lower respiratory tract infection | 486 (11) | 41 (8·4) |
| Respiratory distress syndrome | 263 (6·0) | 136 (52) |
| Congenital anomalies | 215 (4·9) | 55 (26) |
| Meningitis[b] | 112 (2·5) | 11 (9·8) |
| Anaemia | 78 (1·8) | 14 (18) |
| Malnutrition | 36 (0·8) | 1 (2·8) |
| None specified | 69 (1·6) | 4 (5·8) |
| Others | 266 (6·0)[c] | 13 (4·9) |

**Table 2. Discharge diagnosis assigned by clinician.**

For peer review only

[a] Young infant could be assigned up to 2 diagnoses
[b] Meningitis: positive CSF culture, or positive CSF microscopy, or positive CSF antigen test, or elevated CSF WBC count (≥20 in young infants aged 0-28 days OR, ≥10 in young infants aged 29-59 days) PLUS a positive blood culture for a known pathogen
[c] Accidents-3, Acute abdominal obstruction-15, bronchiolitis-12, burns-1, Candidiasis-1, Cellulitis abscess-21, Chickenpox-1, Chromosomal abnormality-5, CNS abscess-1, Conjunctivitis-2, Dehydration-2, Dental problems-1, Diabetes-1, Elective surgery-5, Encephalopathy-9, Epilepsy-7, Extra pulmonary TB-1, Febrile convulsions-5, Feeding difficulty-1, Gastroenteritis-15, Haemolytic uraemic syndrome-1, Hydrocephalus-11, LTB/croup-1, Immunosuppression-17, Malaria-2, Male genital problem-1, Meconium aspiration-33, Neonatal haemorrhage-14, Neonatal tetanus -10, Other skin disease-3, Otitis media-1, Poisoning (organophosphates)-1, Pyogenic arthritis-1, Rabies-1, Rash-4, renal failure-6, trauma/fractures/RTA-11, Urinary tract infection-10, upper respiratory tract infection (URTI)-24, Viral hepatitis-2, Viral infection-3.

*Exposures associated with inpatient death*

Variables assessed for association with inpatient death in univariate models are shown in **Supplementary Table S5**. In the multivariable analysis (**Table 3**), admissions at age ≤2 days and 3–7 days, compared to ≥28 days old, were associated with inpatient deaths. Being born at KCH was not associated with inpatient death, so was not included in the multivariate analysis. Very low admission weight (<1·5kg) and weight 1·5–2·4kg compared to ≥2·5kg were positively associated with inpatient deaths. Signs of clinical severity (bradypnoea, tachypnoea, bradycardia, hypoxia, lower chest wall indrawing, breathing difficulty, weak pulse, impaired consciousness, and hypothermia, but not fever), meningitis, bacteraemia, leucopoenia and leucocytosis but not an HIV antibody positive test (aSHR 1·15 (95%CI 0·81−1·63)) were positively associated with inpatient death. The multivariable model bootstrapped AUC was 0·88 (95%CI 0·86–0·89) **Table 3**. Performance of a multivariable model including only 4,272 single admissions did not differ from the model with multiple admissions (bootstrapped AUC 0·88 (95%CI 0·86–0·89) **Supplementary Table S6**.

**Table 3. Multivariable regression analysis of factors associated with inpatient and post-discharge mortality.**



For peer review only - http://bmjopen.bmj.com/site/about/guidelines.xhtml

| | Inpatient analysis | | Post-discharge analysis | |
|---|---|---|---|---|
| | Adjusted SHR* | P-value | Adjusted HR | P-value |
| **Demographics** | | | | |
| Age in days | | | | |
| 0 to 2 | 2·12 (1·46–3·06) | <0·001 | 1·30 (0·73–2·31) | 0·37 |
| 3 to 7 | 3·88 (2·46–6·10) | <0·001 | 0·80 (0·38–1·68) | 0·56 |
| 8 to 28 | 1·42 (0·90–2·25) | 0·13 | 1·45 (0·81–2·59) | 0·21 |
| >28 | Reference | | Reference | |
| Sex (female) | 0·91 (0·78–1·07) | 0·26 | 0·98 (0·74–1·31) | 0·94 |
| Born at KCH | | | | |
| Yes | ¶ | | Reference | |
| No | ¶ | | 1·59 (1·18–2·14) | 0·003 |
| Admission days (log) | ¶ | | 1·87 (1·54–2·26) | <0·001 |
| Type of discharge | | | | |
| Normal | ¶ | | Reference | |
| Absconded | ¶ | | 3·01 (1·22–7·46) | 0·02 |
| Transferred/referred | ¶ | | 12·8 (8·11–20·2) | <0·001 |
| **Anthropometry** | | | | |
| Weight (kg) | | | | |
| <1.5 | 2·16 (1·75–2·67) | <0·001 | 1·95 (1·38–2·74) | <0·001 |
| 1.5 to <2.5 | 1·42 (1·16–1·74) | 0·001 | 0·82 (0·48–1·42) | 0·49 |
| ≥2.5 | Reference | | Reference | |
| Missing weight | 3·85 (2·59–5·71) | <0·001 | - | |
| **Clinical features** | | | | |
| Axillary temperature | | | | |
| <36˚C | 1·44 (1·17–1·78) | 0·001 | 1·06 (0·74–1·53) | 0·75 |
| 36 to 37·5˚C | Reference | | Reference | |
| >37·5˚C | 1·09 (0·84–1·41) | 0·53 | 0·69 (0.47–0·99) | 0·04 |
| Missing temperature | 1·03 (0·38–2·75) | 0·96 | 1·09 (0·15–8·22) | 0·93 |
| Respiratory rate/min | | | | |
| Bradypnoea | 1·45 (1·09–1·93) | 0·01 | 1·66 (0·76–3·63) | 0·21 |
| Normal | Reference | | Reference | |



For peer review only - http://bmjopen.bmj.com/site/about/guidelines.xhtml

| | | | | |
|---|---|---|---|---|
| Tachypnoea | 0·80 (0·67–0·95) | 0·01 | 1·24 (0·93–1·66) | 0·14 |
| Missing | 1·51 (0·64–3·56) | 0·34 | 0·80 (0·11–5·82) | 0·82 |
| Heart rate/min | | | | |
| Bradycardia | 1·40 (1·08–1·82) | 0·01 | ¶ | |
| Normal | Reference | | | |
| Tachycardia | 1·14 (0·94–1·37) | 0·18 | ¶ | |
| Missing | 0·41 (0·03–5·13) | 0·49 | ¶ | |
| Hypoxia (SaO2 <90%) | 1·62 (1·37–1·92) | <0·001 | ¶ | |
| Capillary refill >2 seconds | 1·34 (0·97–1·86) | 0·08 | ¶ | |
| Lower chest wall indrawing | 1·41 (1·14–1·75) | 0·002 | ¶ | |
| Stridor | 1·93 (0·92–4·03) | 0·08 | ¶ | |
| Breathing difficulty | 1·45 (1·15–1·82) | 0·001 | ¶ | |
| Weak pulse | 1·61 (1·19–2·17) | 0·002 | 2·**22** (1·01–4·89) | 0·04 |
| Bulging fontanel | 2·45 (0·91–6·65) | 0·08 | 2·59 (0·92–7·26) | 0·07 |
| Impaired consciousness | 2·21 (1·72–2·84) | <0·001 | ¶ | |
| Pallor | 1·30 (0·98–1·71) | 0·07 | ¶ | |
| **Laboratory features** | | | | |
| Meningitis | 5·45 (2·50−11·8) | <0·001 | 2·16 (0·73–6·37) | 0·17 |
| HIV antibody positive | 1·15 (0·81–1·63) | 0·43 | 0·94 (0·43–2·05) | 0·87 |
| Bacteraemia | 2·21 (1·51–3·22) | <0·001 | ¶ | |
| White blood cells ($10^{12}$ cells/L) | | | | |
| <4 | 2·17 (1·30−3·62) | 0·003 | ¶ | |
| 4-20 | Reference | | ¶ | |
| >20 | 1·71 (1·43−2·04) | <0·001 | ¶ | |
| unavailable | 1·09 (0.82−1·44) | 0·57 | ¶ | |
| Model performance | | | | |
| Bootstrapped AUC (95% CI) | 0·88 (0·86–0·89) | | 0·76 (0·73–0·80) | |

**SHR;** sub-distribution hazard ratios; *the SHR are from the Fine and Gray's proportional sub-hazards model, HR-Hazard ratio from the shared frailty Cox regression model, **¶;** variables not selected for inclusion in the multivariable model, AUC; area under receiver operating characteristics. Meningitis: positive CSF culture, or positive CSF microscopy,



For peer review only - http://bmjopen.bmj.com/site/about/guidelines.xhtml

or positive CSF antigen test, or elevated CSF WBC count (≥20 in young infants aged 0-28 days OR, ≥10 in young infants aged 29-59 days) PLUS a positive blood culture for a known pathogen

Post-discharge death

There were 3,776 live discharges from 3,640 YI residents of KHDSS, of which 3,760 (from 3,625 individual YI) were followed up for 3,233 infant-years (**Figure 1**). During one-year follow-up, there were 208/3625 (5·7%) deaths: 64·3 (95%CI 56·2–73·7) deaths per 1,000 infant-years. The median [IQR] time to death after discharge was 35 [7–92] days. Of the 208 post-discharge deaths, 101 (49%), 160 (77%), 179 (86%) and 193 (93%) occurred within 1, 3, 6 and 9 months after discharge respectively. The annual YI post-discharge case fatality was 5·4% in 2009 and 6·3% in 2019 without evidence of linear trend (P-value for trend=0·77) (**Figure 2)**.

One hundred and one (49%) of the 208 post-discharge deaths occurred at home without hospital readmission, 67 (32%) occurred during readmission to KCH and 40 (19%) occurred at other health facilities. The five leading assigned causes of deaths for the 67 deaths at KCH were: neonatal sepsis (24%), preterm complications (22%), congenital heart disease (15%), neonatal jaundice (7·4%) and meningitis (7·4%) which were similar to index admission diagnosis **Supplementary Table S7**. Causes of other deaths were unknown.

Overall, we observed 853 (20%) deaths among 4,272 individual YIs: 645 inpatient and 208 post-discharge, hence 24% of deaths were post-discharge.

Exposures assessed for association with post-discharge mortality are shown on **Supplementary Table S8**. In the multivariable Cox regression model, born outside KCH, log days of hospital



For peer review only - http://bmjopen.bmj.com/site/about/guidelines.xhtml

admission, leaving against advice, and referral to more specialized hospital were positively associated with post-discharge mortality. Other exposures associated with post-discharge mortality were low admission weight, fever and weak pulse(**Table 3**). The multivariable model bootstrapped AUC was 0·76 (95%CI 0·73–0·80).

*Subgroup analysis*

In a subgroup analysis including 1,358 admissions of YIs born at KCH, their median [IQR] gestational age was 38 (36–40) weeks and birth weight 2,778 (2,000-3,195) grams respectively. In the univariate regression model, born premature, low birth weight and birth weight Z score <-2 were positively associated with inpatient mortality (**Supplementary Table S9**). In the multivariable model, low birth weight, admission age <8 days, bacteraemia and signs of clinical severity were associated with inpatient mortality (**Supplementary Table S10**).

Among the 1,142 YI followed up for 1,021 child-years of which 41/1,142 (3·6%) died, low birth weight (aHR 2·76 (95%CI 1·30–5·82)) was positively associated with post-discharge mortality in the multivariable model (**Supplementary Table S10**).



For peer review only - http://bmjopen.bmj.com/site/about/guidelines.xhtml

## Discussion

*Trends in admissions and proportions of young infants*

During the study period, we observed a marked increase in YI admissions and proportion of YI among admissions in under-fives increased from around a fifth in 2009, to more than one-third in 2019. However, this did not seem to be associated with the introduction of free maternity care in 2013. Lack of observable effect may be due to challenges faced during policy implementation arising from inadequate expansion of quality health care facilities and resources. Several authors reported an increase in mothers attending Kenyan health facilities for antenatal care and delivery,[11 21] however our results suggest this occurred in the context of a general trend which we previously observed during 1990-2008.[10]

Conversely, the number of admitted children older than 60 days decreased alongside a reduction in local malaria transmission,[22] introduction of routine childhood pneumococcal conjugate and rotavirus immunisation,[23] and expansion in numbers of health facilities in Kilifi County.[24] Variation in annual admissions over the years was due to multiple health workers' strikes.[13] During these periods, the general paediatric ward was closed and only the sickest children were admitted to the paediatric HDU due to limited staffing and bed capacity. The time series analysis indicated an increase in inpatient mortality during strikes (**Figure 3C)**.

The leading diagnoses at admission in our analysis were neonatal sepsis, preterm complications, and birth asphyxia, similar to the period 1990–2008.[10] Over a third of admissions from KCH maternity were preterm and the hospital also received referrals of preterm and very low birthweight infants from sub-county hospitals and local health centres. There are few African



For peer review only - http://bmjopen.bmj.com/site/about/guidelines.xhtml

published datasets of neonatal or YI inpatient diagnoses; in a network of 7 Nigerian and Kenyan hospitals, prematurity accounted for over half (52%), and birth asphyxia almost a quarter (24%) of neonatal admissions.[25] The leading bacterial isolates from blood cultures in our study (*Klebsiella pneumoniae, Escherichia coli*, *Staphylococcus aureus*) were similar to those among YI in rural settings of Tanzania and Burkina Faso.[26] Kenya attained elimination status of maternal and neonatal tetanus in 2018, following immunisation campaigns in high-risk regions.[27] Compared to 1990–2008,[10]neonatal tetanus was uncommon at our centre with only 10 cases in 11 years.

*Inpatient deaths*

The WHO has reported that in 2019, "47% of all under-5 deaths occurred in the newborn period with about one third dying on the day of birth and close to three quarters dying within the first week of life".[28] Delivery by a skilled health worker has been shown to be effective in reducing perinatal mortality.[29] We did not collect data on delivery by a skilled birth attendant but in 2018/9 69% of births in Kilifi County were reported to be attended by skilled health personnel which is slightly higher than the national average.[30] We found YI accounted for more than 60% of under-fives inpatient deaths, similar to a retrospective study of 16 Kenyan public hospitals in which neonatal deaths comprised 66% of inpatient paediatric deaths.[5] We found respiratory distress syndrome, birth asphyxia and preterm complications had the highest inpatient mortality. Mechanical ventilation was not available in Kilifi County Hospital. Improvements in peripartum care of mothers and infants together with appropriate technology such as non-invasive ventilation for management of respiratory complications of preterm birth are priorities for reduction in neonatal mortality in hospitals in LMICs.[5]



For peer review only - http://bmjopen.bmj.com/site/about/guidelines.xhtml

*Post-discharge deaths*

Less than a quarter (24%) of all deaths during 1-year of follow up occurred post-discharge. This reflects a high inpatient (16%) case fatality rate with many very early inpatient deaths compared to 6·6% in children aged ≥60 days.[7] Nevertheless, the post-discharge YI mortality rate (64·3 per 1,000 child/years) was more than twice that of a cohort of children aged 2–59 months admitted to KCH between 2007- 2015.[31] This reflects post-discharge mortality rates being highest in younger age groups, such as in Tanzania among under 1-year olds: 72 per 1,000 child/years (95%C.I. 67·2–77·2) falling to 6·9 (95%C.I. 5·5–8·7) per 1,000 child/years in 4 to <5 year olds.[32]

A greater proportion of YI post-discharge deaths occurred in hospital than among older children,[7] implying that caregivers may be more likely to seek re-admission for YI or may live closer to KCH. About half of post-discharge deaths occurred within the first month, highlighting the need for formal 'down-referral' for continuity of care after discharge in high risk YI.

Analysis of exposures revealed that some were common for both inpatient and post-discharge mortality: low admission weight, axillary temperature, and respiratory rate. Birth weight was not available for most YI but low admission weight <2·5kg was common (40%) in our participants. In young infants it is difficult to distinguish low birth weight from malnutrition, but we have reported the higher case fatality rates in the lower admission weight categories (Tables S3 and S4). Of known causes of post-discharge deaths, leading ones were related to problems in the early neonatal period.

*Strengths and limitations of the study*



For peer review only - http://bmjopen.bmj.com/site/about/guidelines.xhtml

Strengths of this study are large sample size, systematic collection of data and linkage to a well-established demographic surveillance system, with few losses to follow up. Limitations are lack of accurate gestational age estimation, unknown birthweight of most participants and that individual socioeconomic data were unavailable. We did not have clinical data collected at discharge, which may be of value in taking a risk-based approach to post-discharge care. This analysis is from a single hospital and excludes residents outside KHDSS who may have different exposures and risks.

*Conclusions*

Neonatal and YI admissions account for an increasing proportion of inpatient paediatric admissions, and their overall mortality rate remains high. Post-discharge mortality accounts for a lower proportion of all deaths than hospital admissions aged 2 to 59 months but the post-discharge mortality rate among young infants is higher.[31 33] This is likely because of the predominance of fatal neonatal conditions such as extreme prematurity or birth asphyxia. Services to address post-discharge mortality are needed and should focus on infants at higher risk.

*Acknowledgements*



For peer review only - http://bmjopen.bmj.com/site/about/guidelines.xhtml

We thank the parents, patients, and staff of Kilifi County Hospital and the KEMRI-Wellcome Trust Research Programme for their participation in the study. This study is published with the permission of the Director, KEMRI. This work was supported, in whole or in part, by the Bill & Melinda Gates Foundation [Grant Number INV-00791]. Under the grant conditions of the Foundation, a Creative Commons Attribution 4.0 Generic License has already been assigned to the Author Accepted Manuscript version that might arise from this submission.

I, the Submitting Author has the right to grant and does grant on behalf of all authors of the Work (as defined in the below author licence), an exclusive licence and/or a non-exclusive licence for contributions from authors who are: i) UK Crown employees; ii) where BMJ has agreed a CC-BY licence shall apply, and/or iii) in accordance with the terms applicable for US Federal Government officers or employees acting as part of their official duties; on a worldwide, perpetual, irrevocable, royalty-free basis to BMJ Publishing Group Ltd ("BMJ") its licensees and where the relevant Journal is co-owned by BMJ to the co-owners of the Journal, to publish the Work in BMJ Global Health and any other BMJ products and to exploit all rights, as set out in our licence.

The Submitting Author accepts and understands that any supply made under these terms is made by BMJ to the Submitting Author unless you are acting as an employee on behalf of your employer or a postgraduate student of an affiliated institution which is paying any applicable article publishing charge ("APC") for Open Access articles. Where the Submitting Author wishes to make the Work available on an Open Access basis (and intends to pay the relevant APC), the terms of reuse of such Open Access shall be governed by a Creative Commons licence – details of these licences and which Creative Commons licence will apply to this Work are set out in our licence referred to above.

For peer review only

## Figures

**Figure 1. Flow of study participants.**

**Figure 2. Annual proportion of YI admissions to all admissions <60 months, inpatient case fatality ratio (CFR) and post-discharge CFR.**

Proportions are plotted with 95% confidence intervals.

**Figure 3. A: Monthly hospital admissions (with predicted mean temporal trend), B: Monthly case fatality rates (with predicted mean temporal trend), C: Monthly young infant inpatient case fatality before and after June 2013 and D: Monthly proportions of young infants to admissions <60 months old.**

Ethics approval and consent to participate

Written consent was provided by the caregivers of all the surveillance study participants. Ethical approval to conduct this analysis was granted by the Kenya Medical Research Institute (KEMRI) National Ethics Review Committee (SCC 2778).

Consent for publication – not applicable

Availability of data and materials



For peer review only - http://bmjopen.bmj.com/site/about/guidelines.xhtml

Data are available in a public, open access repository. Deidentified participant data and analysis code have been deposited and may be requested at the Harvard Dataverse via this link https://doi.org/10.7910/DVN/0XJVFX

Competing interests

JAB declares the following: Chair of the DSMB for **"Efficacy and safety of whole-body chlorhexidine cleansing in reducing bacterial skin colonisation of hospitalised neonates - a pilot trial".** *St George's, University of London and global sites;* Treasurer of the Commonwealth Society for Paediatric Gastroenterology & Nutrition Other authors declare they have no competing interests.

Funding

Authors NM, AN, NO, MO, and staffing, facilities, and resources were funded by the Wellcome Trust (203077_Z_16_Z). MM was supported by a Wellcome Trust International Intermediate Fellowship (221997/Z/20/Z). JAB was supported by the Medical Research Council–Department for International Development–Wellcome Trust Joint Global Health Trials scheme (MR/M007367/1). JAB and MN were supported by the Bill & Melinda Gates Foundation (OPP1131320). CWO was supported by the Drugs for Neglected Diseases initiative/Global Antibiotic Research and Development Partnership (OXF-DND02). AT was supported by Crosslinks.

Role of the funding source

The funders did not have a role in study design, in the collection, analysis, and interpretation of data, in writing the report, or in the decision to submit the paper for publication.

Author contributions



For peer review only - http://bmjopen.bmj.com/site/about/guidelines.xhtml

AT: Conceptualization, investigation, methodology, formal analysis, writing – original draft, writing– review & editing; MN: Conceptualization, methodology, data curation, formal analysis, visualisation , writing – original draft, writing– review & editing; CWO: Conceptualization, investigation, methodology, formal analysis, validation, writing – original draft, writing– review & editing; AN: Investigation, methodology, project administration, writing– review & editing; MM: Conceptualization, methodology, writing– review & editing; NM: Investigation, project administration, funding acquisition, resources, supervision, writing– review & editing; NO: Data curation, writing– review & editing, MO: Data curation, writing– review & editing; JAB: Conceptualisation, investigation, methodology, funding acquisition, supervision, validation, writing– review & editing.

AT and MN contributed equally to this paper. AT, the guarantor, accepts full responsibility for the finished work and the conduct of the study, had access to the data, and controlled the decision to publish.

## References

1. UNICEF. Levels & Trends in Child Mortality: Report 2020, Estimates developed by the United Nations Inter-agency Group for Child Mortality Estimation. New York: United Nations Children's Fund, 2020.

2. Collaborators GBDCM. Global, regional, national, and selected subnational levels of stillbirths, neonatal, infant, and under-5 mortality, 1980-2015: a systematic analysis for the Global Burden



For peer review only - http://bmjopen.bmj.com/site/about/guidelines.xhtml

501 of Disease Study 2015. *Lancet* 2016;388(10053):1725-74. doi: 10.1016/S0140-6736(16)31575-6
502 [published Online First: 2016/10/14]
503 3. English M, Ngama M, Musumba C, et al. Causes and outcome of young infant admissions to a Kenyan
504 district hospital. *Arch Dis Child* 2003;88(5):438-43. doi: 10.1136/adc.88.5.438
505 4. van den Boogaard W, Manzi M, Harries AD, et al. Causes of pediatric mortality and case-fatality rates
506 in eight Médecins Sans Frontières-supported hospitals in Africa. *Public Health Action*
507 2012;2(4):117-21. doi: 10.5588/pha.12.0038
508 5. Irimu G, Aluvaala J, Malla L, et al. Neonatal mortality in Kenyan hospitals: a multisite, retrospective,
509 cohort study. *BMJ Glob Health* 2021;6(5) doi: 10.1136/bmjgh-2020-004475
510 6. Obiero CW, Mturi N, Mwarumba S, et al. Clinical features to distinguish meningitis among young
511 infants at a rural Kenyan hospital. *Arch Dis Child* 2021;106(2):130-36. doi: 10.1136/archdischild-
512 2020-318913 [published Online First: 20200820]
513 7. CHAIN, Network. Childhood mortality during and after acute illness in Africa and south Asia: a
514 prospective cohort study. *Lancet Glob Health* 2022;10(5):e673-e84. doi: 10.1016/s2214-
515 109x(22)00118-8
516 8. Nemetchek B, English L, Kissoon N, et al. Paediatric postdischarge mortality in developing countries: a
517 systematic review. *BMJ open* 2018;8(12):e023445. doi: 10.1136/bmjopen-2018-023445
518 [published Online First: 2018/12/30]
519 9. Moisi JC, Gatakaa H, Berkley JA, et al. Excess child mortality after discharge from hospital in Kilifi,
520 Kenya: a retrospective cohort analysis. *Bull World Health Organ* 2011;89(10):725-32, 32A. doi:
521 10.2471/BLT.11.089235 [published Online First: 2011/11/16]
522 10. Mwaniki MK, Gatakaa HW, Mturi FN, et al. An increase in the burden of neonatal admissions to a
523 rural district hospital in Kenya over 19 years. *BMC Public Health* 2010;10:591. doi:
524 10.1186/1471-2458-10-591 [published Online First: 2010/10/12]

For peer review only



For peer review only - http://bmjopen.bmj.com/site/about/guidelines.xhtml

11. Lang'at E, Mwanri L, Temmerman M. Effects of implementing free maternity service policy in Kenya: an interrupted time series analysis. *BMC Health Serv Res* 2019;19(1):645. doi: 10.1186/s12913-019-4462-x [published Online First: 2019/09/08]

12. KEMRI-WellcomeTrust, Programme. R. Health-research-linked-to-a-demographic-surveillance-system [Available from: https://kemri-wellcome.org/programme/health-research-linked-to-a-demographic-surveillance-system/ accessed May 10 2021.

13. Ong'ayo G, Ooko M, Wang'ondu R, et al. Effect of strikes by health workers on mortality between 2010 and 2016 in Kilifi, Kenya: a population-based cohort analysis. *Lancet Glob Health* 2019;7(7):e961-e67. doi: 10.1016/s2214-109x(19)30188-3 [published Online First: 2019/05/28]

14. Berkley JA, Lowe BS, Mwangi I, et al. Bacteremia among children admitted to a rural hospital in Kenya. *N Engl J Med* 2005;352(1):39-47. doi: 10.1056/NEJMoa040275 [published Online First: 2005/01/07]

15. Ministry of Health, Kenya. Basic Paediatric Protocols 4th ed, 2016.

[dataset] 16. Ngari M, Talbert A, Ouma N, et al. Replication Data for: Inpatient and post-discharge mortality among young infants admitted to a rural Kenyan hospital.: Harvard Dataverse, June 9 2022. https://doi.org/10.7910/DVN/0XJVFX

17. Cuzick J. A Wilcoxon-type test for trend. *Stat Med* 1985;4(1):87-90. doi: 10.1002/sim.4780040112

18. Fine JP, Gray RJ. A Proportional Hazards Model for the Subdistribution of a Competing Risk. *J Am Stat Assoc* 1999;94(446):496-509. doi: 10.1080/01621459.1999.10474144

19. Seale AC, Barsosio HC, Koech AC, et al. Embedding surveillance into clinical care to detect serious adverse events in pregnancy. *Vaccine* 2015;33(47):6466-8. doi: 10.1016/j.vaccine.2015.07.086 [published Online First: 20150805]



For peer review only - http://bmjopen.bmj.com/site/about/guidelines.xhtml

20. Papageorghiou AT, Kennedy SH, Salomon LJ, et al. The INTERGROWTH-21(st) fetal growth standards: toward the global integration of pregnancy and pediatric care. *Am J Obstet Gynecol* 2018;218(2S):S630-S40. doi: 10.1016/j.ajog.2018.01.011 [published Online First: 2018/02/10]

21. Orangi S, Kairu A, Malla L, et al. Impact of free maternity policies in Kenya: an interrupted time-series analysis. *BMJ Glob Health* 2021;6(6) doi: 10.1136/bmjgh-2020-003649

22. Njuguna P, Maitland K, Nyaguara A, et al. Observational study: 27 years of severe malaria surveillance in Kilifi, Kenya. *BMC Med* 2019;17(1):124. doi: 10.1186/s12916-019-1359-9 [published Online First: 2019/07/10]

23. Hammitt LL, Etyang AO, Morpeth SC, et al. Effect of ten-valent pneumococcal conjugate vaccine on invasive pneumococcal disease and nasopharyngeal carriage in Kenya: a longitudinal surveillance study. *Lancet (London, England)* 2019;393(10186):2146-54. doi: 10.1016/s0140-6736(18)33005-8 [published Online First: 20190415]

24. Munywoki J, Kagwanja N, Chuma J, et al. Tracking health sector priority setting processes and outcomes for human resources for health, five-years after political devolution: a county-level case study in Kenya. *Int J Equity Health* 2020;19(1):165. doi: 10.1186/s12939-020-01284-3 [published Online First: 20200921]

25. Nabwera HM, Wang D, Tongo OO, et al. Burden of disease and risk factors for mortality amongst hospitalized newborns in Nigeria and Kenya. *PLoS One* 2021;16(1):e0244109. doi: 10.1371/journal.pone.0244109 [published Online First: 20210114]

26. Mduma E, Halidou T, Kaboré B, et al. Etiology of severe invasive infections in young infants in rural settings in sub-Saharan Africa. *PLoS One* 2022;17(2):e0264322. doi: 10.1371/journal.pone.0264322 [published Online First: 20220225]

For peer review only



For peer review only - http://bmjopen.bmj.com/site/about/guidelines.xhtml

27. WHO Afro. Kenya now eliminates maternal and neonatal tetanus 2019 [Available from: https://www.afro.who.int/news/kenya-now-eliminates-maternal-and-neonatal-tetanus accessed February 2 2022.

28. World Health Organization. Newborns: improving survival and well-being 2020 [Available from: https://www.who.int/news-room/fact-sheets/detail/newborns-reducing-mortality accessed May 13 2022.

29. Bhutta ZA, Das JK, Bahl R, et al. Can available interventions end preventable deaths in mothers, newborn babies, and stillbirths, and at what cost? *Lancet (London, England)* 2014;384(9940):347-70. doi: 10.1016/s0140-6736(14)60792-3 [published Online First: 20140519]

30. ThinkWell. A reivew of the Transforming Health Systems for Universal Care project in Kilifi County, 2021.

31. Talbert A, Ngari M, Bauni E, et al. Mortality after inpatient treatment for diarrhea in children: a cohort study. *BMC Med* 2019;17(1):20. doi: 10.1186/s12916-019-1258-0 [published Online First: 20190128]

32. Mukasa O, Masanja H, DeSavigny D, et al. A cohort study of survival following discharge from hospital in rural Tanzanian children using linked data of admissions with community-based demographic surveillance. *Emerg Themes Epidemiol* 2021;18(1):4. doi: 10.1186/s12982-021-00094-4 [published Online First: 20210318]

33. Ngari MM, Fegan G, Mwangome MK, et al. Mortality after Inpatient Treatment for Severe Pneumonia in Children: a Cohort Study. *Paediatric and perinatal epidemiology* 2017;31(3):233-42. doi: 10.1111/ppe.12348 [published Online First: 2017/03/21]



For peer review only - http://bmjopen.bmj.com/site/about/guidelines.xhtml

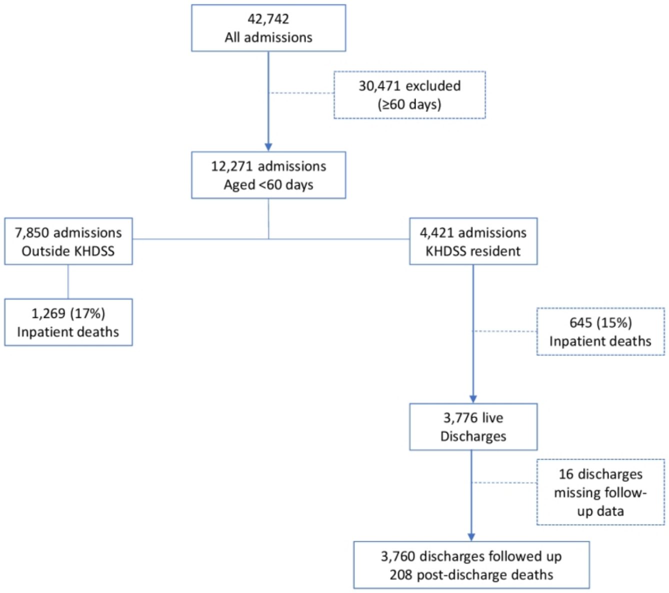


275x190mm (200 x 200 DPI)

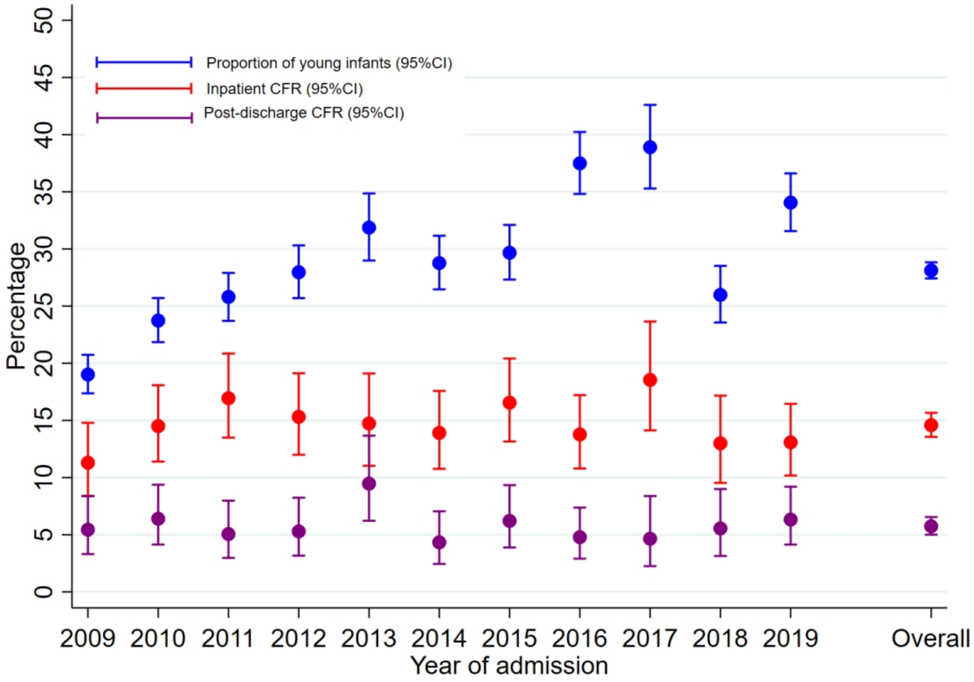


547x398mm (72 x 72 DPI)

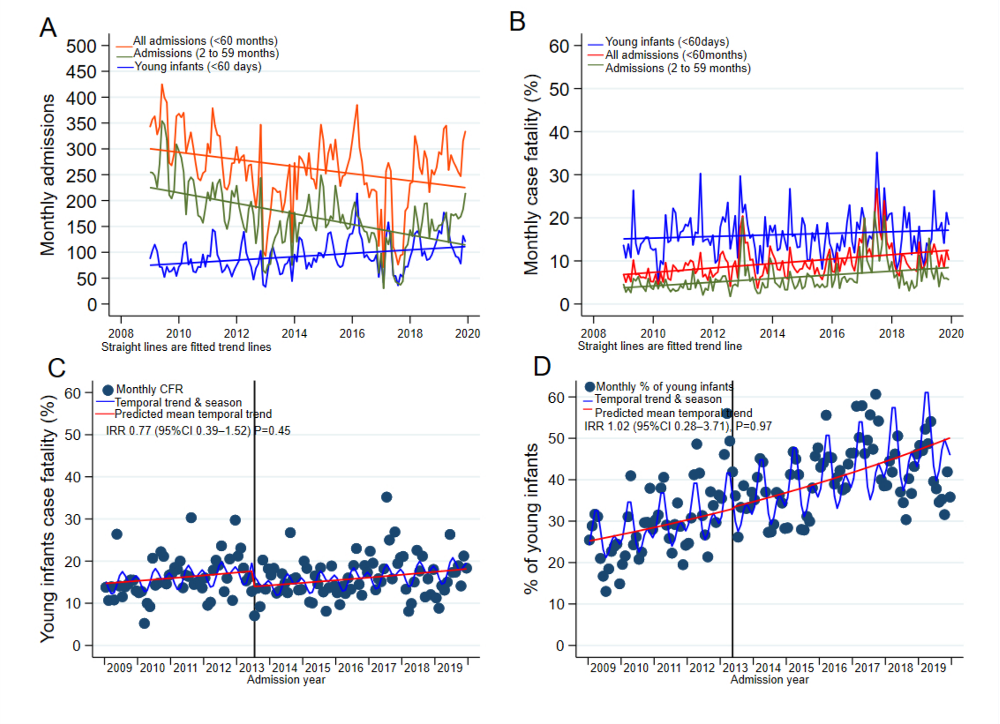


399x290mm (72 x 72 DPI)

**Supplementary materials**

**Table of Contents**


| | |
|---|---|
| Table S1. List of health workers strikes during study period. | Page 2 |
| Table S2. Proportion of missing data | 3 |
| Table S3 Pathogens isolated from blood and CSF cultures of young infants resident in KHDSS during inpatient period. | 4 |
| Table S4. Annual admissions and case fatality ratios | 5 |
| Table S5. Univariate analysis of admission features associated with inpatient deaths. | 6,7 |
| Table S6. Multivariable regression analysis of factors associated with inpatient mortality (single vs multiple admissions). | 8,9 |
| Table S7. Estimated causes of post-discharge deaths during readmission at KCH (67 deaths). | 10 |
| Table S8. Univariate analysis of admission features associated with post-discharge deaths. | 11,12 |
| Table S9. Univariate analysis of admission features associated with inpatient deaths among babies born at KCH only. | 13,14 |
| Table S10. Multivariable regression analysis of factors associated with inpatient and post-discharge mortality among children born at KCH only. | 15 |
| Figure S1. A: Monthly all young infant admissions, B: all admissions (<60 months old), C: all admissions (<60 months old) case fatality and D: 2–59 months old case fatality before and after July 2013. | 16 |




For peer review only - http://bmjopen.bmj.com/site/about/guidelines.xhtml

Table S1. **List of health workers strikes during study period.**

| Strike dates | Health workers on strike | Duration of strike in days |
|---|---|---|
| 5 to 13 December 2011 | Doctors | 9 |
| 1 to 15 March 2012 | Nurses | 15 |
| 13 September to 4 October 2012 | Doctors | 22 |
| 3 December 2012 to 13 January 2013 | Nurses | 42 |
| 16 January to 11 February 2013 | Nurses | 26 |
| 10 to 23 December 2013 | Doctors & nurses | 14 |
| 5 to 14 December 2016 | Nurses | 11 |
| 5 December 2016 to 15 March 2017 | Doctors | 102 |
| 5 June to 2 November 2017 | Nurses | 150 |

For peer review only



For peer review only - http://bmjopen.bmj.com/site/about/guidelines.xhtml

Table S2. **Proportion of missing data**

| **N=4,421** | N missing | % missing |
|---|---|---|
| Demographics | | |
| Age in days | 0 | |
| Sex (female) | 0 | |
| BCG scar | 76 | 1.7 |
| Reported born premature | 321 | 7.3 |
| Reported low birth weight | 322 | 7.3 |
| **Anthropometry** | | |
| Weight (kg) | 43 | 1.0 |
| MUAC (cm) | 126 | 2.9 |
| **Clinical features** | | |
| Axillary temperature | 34 | 0.8 |
| Tachypnea | 208 | 4.7 |
| Tachycardia | 43 | 1.0 |
| Hypoxia (SaO2 <90%) | 20 | 0.5 |
| Lower chest wall indrawing | 68 | 1.5 |
| Wheeze | 69 | 1.6 |
| Stridor | 71 | 1.6 |
| Breathing difficulty | 64 | 1.5 |
| Cyanosis | 70 | 1.6 |
| Capillary refill ≥2 seconds | 34 | 0.8 |
| Temperature gradient | 81 | 1.8 |
| Weak pulse | 71 | 1.6 |
| Lethargy | 70 | 1.6 |
| Impaired consciousness | 70 | 1.6 |
| Bulging fontanel | 72 | 1.6 |
| Stiff neck | 71 | 1.6 |
| Convulsions | 64 | 1.5 |
| Sunken eyes | 73 | 1.7 |
| Reduced skin turgor | 71 | 1.6 |
| Pallor | 70 | 1.6 |
| **Laboratory features** | | |
| HIV antibody positive | 756 | 17 |
| Malaria slide positive | 372 | 8.4 |
| Bacteraemia | 2281 | 52 |
| Haemoglobin | 448 | 10 |
| WBC | 449 | 10 |
| Platelets | 448 | 10 |
| Blood glucose (mmols/L) | 1433 | 32 |

For peer review only



For peer review only - http://bmjopen.bmj.com/site/about/guidelines.xhtml

Table S3 **Pathogens isolated from blood and CSF cultures of young infants resident in KHDSS during inpatient period**.

| **Blood culture Isolates** | | **CSF culture isolates** | |
|---|---|---|---|
| **Pathogen (N=178)** | No. (%) | **Pathogen (N=24)** | No. (%) |
| *Klebsiella pneumoniae* | 53 (30) | *Escherichia coli* | 6 (25) |
| *Escherichia coli* | 25 (14) | Group B Streptococcus | 6 (25) |
| *Staphylococcus aureus* | 22 (12) | *Klebsiella pneumoniae* | 3 (13) |
| Group B Streptococcus | 19 (11) | *Streptococcus pneumoniae* | 3 (13) |
| Non-typhoidal Salmonella species | 9 (5.1) | *Enterobacter cloacae* | 3 (13) |
| *Enterobacter cloacae* | 8 (4.5) | Non-typhoidal Salmonella species | 2 (8.2) |
| *Pseudomonas aeruginosa* | 6 (3.4) | *Acinetobacter lwoffi* | 1 (4.2) |
| *Streptococcus pneumoniae* | 5 (2.8) | | |
| *Streptococcus pyogenes* | 3 (1.7) | | |
| Acinetobacter species | 3 (1.7) | | |
| *Aeromonas hydrophila* | 3 (1.7) | | |
| Group A Streptococcus | 3 (1.7) | | |
| *Serratia marcescens* | 2 (1.1) | | |
| *Acinetobacter calcoaceticus/baumannii* | 2 (1.1) | | |
| *Acinetobacter lwoffi* | 1 (0.6) | | |
| *Aeromonas sobria* | 1 (0.6) | | |
| *Chryseobacterium indologenes* | 1 (0.6) | | |
| *Enterobacter aerogenes* | 1 (0.6) | | |
| Enterococci species | 1 (0.6) | | |
| *Haemophilus influenzae* | 1 (0.6) | | |
| *Proteus mirabilis* | 1 (0.6) | | |

CSF; cerebrospinal fluid, Out of the 33 Meningitis cases, only the 24 presented had positive CSF culture.

For peer review only



For peer review only - http://bmjopen.bmj.com/site/about/guidelines.xhtml

Table S4. **Annual admissions and case fatality ratios (CFR).**

| Admissions/Year | **2009** | **2010** | **2011** | **2012** | **2013** | **2014** | **2015** | **2016** | **2017** | **2018** | **2019** |
|---|---|---|---|---|---|---|---|---|---|---|---|
| <60 days | 407 | 455 | 425 | 418 | 319 | 424 | 429 | 472 | 275 | 323 | 474 |
| 2 to 59 months | 1734 | 1462 | 1222 | 1177 | 682 | 1050 | 1017 | 787 | 432 | 920 | 918 |
| Proportion of YI admissions | 19% | 24% | 26% | 28% | 32% | 29% | 30% | 37% | 39% | 26% | 34% |
| YI inpatient deaths | 46 | 66 | 72 | 64 | 47 | 59 | 71 | 65 | 51 | 42 | 62 |
| YI inpatient CFR | 11% | 15% | 17% | 15% | 15% | 14% | 17% | 14% | 19% | 13% | 13% |
| YI post-discharge 1-year deaths | 19 | 24 | 17 | 18 | 25 | 15 | 21 | 19 | 10 | 15 | 25 |
| YI Post-discharge 1- year CFR | 5.4% | 6.4% | 5.1% | 5.3% | 9.5% | 4.3% | 6.2% | 4.8% | 4.7% | 5.6% | 6.3% |

For peer review only



For peer review only - http://bmjopen.bmj.com/site/about/guidelines.xhtml

Table S5. **Univariate analysis of admission features associated with inpatient deaths.**

| **N=4,421** | **Deaths (N=645) N (%)** | **Crude SHR** | **P-value** | **Scaled Schoenfeld residuals P-value** |
|---|---|---|---|---|
| Demographics | | | | |
| Age in days | | | | |
| 0 to 2 | 511 (19) | 3.31 (2.39–4.58) | <0.001 | 0.14 |
| 3 to 7 | 56 (12) | 2.07 (1.38–3.11) | <0.001 | |
| 8 to 28 | 40 (6.8) | 1.15 (0.74–1.78) | 0.54 | |
| >28 | 38 (6.0) | Reference | | |
| Sex (female) | 268 (14) | 0.99 (0.91–1.09) | 0.93 | 0.77 |
| Reported born premature | 294 (29) | 2.53 (2.32–3.77) | <0.001 | 0.06 |
| Reported low birth weight | 222 (38) | 3.25 (2.97–3.56) | <0.001 | 0.18 |
| Born at KCH | | | | |
| Yes | 382 (14) | Reference | | |
| No | 263 (16) | 1.13 (0.97–1.32) | 0.12 | 0.61 |
| **Anthropometry** | | | | |
| Weight (kg) | | | | |
| <1.5 | 213 (38) | 4.95 (4.13–5.93) | <0.001 | 0.09 |
| 1.5 to <2.5 | 174 (15) | 1.86(1.53–2.26) | <0.001 | |
| ≥2.5 | 229 (8.5) | Reference | | |
| Missing weight | 29 (67) | 10.7 (7.60–14.9) | <0.001 | |
| MUAC (cm) | | | | |
| <9.0 | 333 (25) | 3.57 (3.07–4.15) | <0.001 | 0.08 |
| 9 to 10 | 106 (12) | 1.73 (1.45–2.07) | <0.001 | |
| 10 to 11 | 96 (9.3) | 1.48 (1.24–1.77) | <0.001 | |
| ≥11 | 75 (7.1) | Reference | | |
| Missing MUAC | 35 (28) | 3.89 (3.02–5.00) | <0.001 | |
| **Clinical features** | | | | |
| Axillary temperature | | | | |
| <36˚C | 390 (29) | 3.13 (2.82–3.47) | <0.001 | 0.34 |
| 36 to 37.5˚C | 159 (9.3) | Reference | | |
| >37.5˚C | 84 (6.3) | 0.77 (0.67–0.88) | <0.001 | |
| Missing temperature | 12 (35) | 3.78 (2.65–5.39) | <0.001 | |
| Respiratory rate/min | | | | |
| Bradypnoea | 108 (57) | 6.09 (4.98–7.46 | <0.001 | 0.71 |
| Normal | 329 (12) | Reference | | |
| Tachypnoea | 197 (13) | 1.07 (0.90–1.27) | 0.45 | |
| Missing | 11 (11) | 0.91 (0.51–1.65) | 0.76 | |
| Heart rate/min | | | | |
| Bradycardia | 74 (47) | 4.05 (3.21–5.11) | <0.001 | 0.50 |
| Normal | 403 (14) | Reference | | |
| Tachycardia | 163 (12) | 0.88 (0.73–1.05) | 0.15 | |
| Missing | 5 (23) | 1.68 (0.72–3.93) | 0.23 | |
| Hypoxia (SaO2 <90%) | 309 (33) | 3.91 (3.58–4.26) | <0.001 | 0.48 |
| Lower chest wall indrawing | 448 (22) | 2.86 (2.60–3.15) | <0.001 | 0.41 |
| Wheeze | 0 | - | | |



For peer review only - http://bmjopen.bmj.com/site/about/guidelines.xhtml

| | | | | |
|---|---|---|---|---|
| Stridor | 6 (32) | 1.48 (0.89–2.47) | 0.13 | 0.23 |
| Breathing difficulty | 481 (22) | 3.62 (3.26–4.02) | <0.001 | 0.28 |
| Cyanosis | 98 (47) | 4.01 (3.55–4.53) | <0.001 | 0.14 |
| Capillary refill >2 seconds | 61 (58) | 5.61 (4.41–7.14) | <0.001 | 0.54 |
| Temperature gradient | 98 (38) | 2.85 (2.51–3.23) | <0.001 | 0.36 |
| Weak pulse | 108 (69) | 5.85 (5.21–6.57) | <0.001 | 0.71 |
| Lethargy | 64 (20) | 1.20 (1.04–1.40) | 0.02 | 0.34 |
| Impaired consciousness | 140 (56) | 5.43 (4.91–6.00) | <0.001 | 0.42 |
| Bulging fontanel | 6 (19) | 1.34 (0.90–1.99) | 0.15 | 0.08 |
| Stiff neck | 4 (40) | 2.08 (1.30–3.32) | 0.002 | 0.17 |
| Convulsions | 16 (8.1) | 0.75 (0.61–0.93) | 0.01 | 0.53 |
| Sunken eyes | 5 (11) | 1.04 (0.69–1.56) | 0.85 | 0.52 |
| Reduced skin turgor | 21 (22) | 1.18 (0.91–1.52) | 0.22 | 0.56 |
| Pallor | 72 (33) | 2.47 (2.15–2.83) | <0.001 | 0.63 |
| **Laboratory features** | | | | |
| Meningitis | 8 (24) | 8.06 (3.96–16.4) | <0.001 | 0.13 |
| Anaemia (haemoglobin <11 g/dl) | 50 (11) | 0.67 (0.51–0.90) | 0.007 | 0.51 |
| HIV antibody positive | 29 (20) | 1.39 (1.13–1.71) | 0.002 | 0.28 |
| Malaria slide positive | 0 | - | | |
| Bacteraemia | 63 (37) | 2.92 (2.10–4.06) | <0.001 | 0.13 |
| Blood glucose (mmols/L) | | | | |
| <2.6 | 137 (16) | 1.18 (1.04–1.33) | 0.009 | |
| 2.6 to 7.0 | 229 (12) | Reference | | 0.42 |
| >7.0 | 71 (31) | 2.85 (2.47–3.29) | <0.001 | |
| Missing blood glucose | 208 (15) | 1.18 (1.06–1.32) | 0.002 | |
| White blood cells ($10^{12}$ cells/L) | | | | |
| <4 | 14 (26) | 2.43 (1.47–4.03) | 0.001 | |
| 4-20 | 362 (11) | Reference | | 0.70 |
| >20 | 210 (30) | 2.97 (2.52–3.50) | <0.001 | |
| Missing | 59 (13) | 1.19 (0.91–1.57) | 0.20 | |
| Platelets ($10^9$ cells/L) | | | | |
| <150 | 139 (24) | 1.89 (1.57–2.27) | <0.001 | |
| ≥150 | 447 (13) | Reference | | 0.10 |
| Missing | 59 (13) | 1.01 (0.77–1.32) | 0.95 | |
| SHR: sub-distribution hazard ratios; the SHR are from the Fine and Gray's proportional sub-hazards model. | | | | |



For peer review only - http://bmjopen.bmj.com/site/about/guidelines.xhtml

Table S6. **Multivariable regression analysis of factors associated with inpatient mortality (single vs multiple admissions).**

| | Multiple admissions (N=4421) | | Single admissions (N=4272) | |
|---|---|---|---|---|
| | Adjusted SHR* | P-value | Adjusted SHR* | P-value |
| Demographics | | | | |
| Age in days | | | | |
| 0 to 2 | 2·12 (1·46–3·06) | <0·001 | 2·14 (1·49–3·10) | <0·001 |
| 3 to 7 | 3·88 (2·46–6·10) | <0·001 | 3·92 (2·48–5·16) | <0·001 |
| 8 to 28 | 1·42 (0·90–2·25) | 0·13 | 1·50 (0·95–2·38) | 0·08 |
| >28 | Reference | | Reference | |
| Sex (female) | 0·91 (0·78–1·07) | 0·26 | 0·90 (0·77–1·06) | 0·20 |
| **Anthropometry** | | | | |
| Weight (kg) | | | | |
| <1.5 | 2·16 (1·75–2·67) | <0·001 | 2·26 (1·83–2·79) | <0·001 |
| 1.5 to <2.5 | 1·42 (1·16–1·74) | 0·001 | 1·43 (1·17–1·76) | <0·001 |
| ≥2.5 | Reference | | Reference | |
| Missing weight | 3·85 (2·59–5·71) | <0·001 | 3·78 (2·56–5·58) | <0·001 |
| **Clinical features** | | | | |
| Axillary temperature | | | | |
| <36˚C | 1·44 (1·17–1·78) | 0·001 | 1·45 (1·17–1·79) | 0·001 |
| 36 to 37·5˚C | Reference | | Reference | |
| >37·5˚C | 1·09 (0·84–1·41) | 0·53 | 1·07 (0.83–1·39) | 0·57 |
| Missing temperature | 1·03 (0·38–2·75) | 0·96 | 0·9**2** (0·33–2·51) | 0·87 |
| Respiratory rate/min | | | | |
| Bradypnoea | 1·45 (1·09–1·93) | 0·01 | 1·44 (1·08–1·92) | 0·01 |
| Normal | Reference | | Reference | |
| Tachypnoea | 0·80 (0·67–0·95) | 0·01 | 0·79 (0·66–0·94) | 0·009 |
| Missing | 1·51 (0·64–3·56) | 0·34 | 1·48 (0·62–3·55) | 0·38 |
| Heart rate/min | | | | |
| Bradycardia | 1·40 (1·08–1·82) | 0·01 | 1·42 (1·09–1·85) | 0·008 |
| Normal | Reference | | Reference | |
| Tachycardia | 1·14 (0·94–1·37) | 0·18 | 1·15 (0·96–1·39) | 0·14 |
| Missing | 0·41 (0·03–5·13) | 0·49 | 0·45 (0·04–5·14) | 0·52 |
| Hypoxia (SaO2 <90%) | 1·62 (1·37–1·92) | <0·001 | 1·60 (1·36–1·90) | <0·001 |
| Capillary refill >2 seconds | 1·34 (0·97–1·86) | 0·08 | 1·31 (0·94–1·83) | 0·11 |
| Lower chest wall indrawing | 1·41 (1·14–1·75) | 0·002 | 1·42 (1·14–1·77) | 0·002 |
| Stridor | 1·93 (0·92–4·03) | 0·08 | 1·85 (0·89–3·85) | 0·10 |
| Breathing difficulty | 1·45 (1·15–1·82) | 0·001 | 1·44 (1·15–1·81) | 0·002 |
| Weak pulse | 1·61 (1·19–2·17) | 0·002 | 1·61 (1·18–2·18) | 0·002 |
| Bulging fontanel | 2·45 (0·91–6·65) | 0·08 | 2·41 (0·90–6·55) | 0·08 |
| Impaired consciousness | 2·21 (1·72–2·84) | <0·001 | 2·17 (1·68–2·78) | <0·001 |
| Pallor | 1·30 (0·98–1·71) | 0·07 | 1·28 (0·97–1·69) | 0·08 |
| **Laboratory features** | | | | |
| Meningitis | 5·45 (2·50–11·8) | <0·001 | 5·18 (2·39–11·2) | <0·001 |
| HIV antibody positive | 1·15 (0·81–1·63) | 0·43 | 1·14 (0·80–1·62) | 0·47 |



For peer review only - http://bmjopen.bmj.com/site/about/guidelines.xhtml

| | | | | |
|---|---|---|---|---|
| Bacteraemia | 2·21 (1·51–3·22) | <0·001 | 2·20 (1·51–3·20) | <0·001 |
| White blood cells ($10^{12}$ cells/L) | | | | |
| <4 | 2·17 (1·30–3·62) | 0·003 | 2·11 (1·27–3·50) | 0·003 |
| 4-20 | Reference | | Reference | |
| >20 | 1·71 (1·43–2·04) | <0·001 | 1·70 (1·43–2·03) | <0·001 |
| unavailable | 1·09 (0.82–1·44) | 0·57 | 1·06 (0.79–1·41) | 0·71 |
| Model performance | | | | |
| Bootstrapped AUC (95% CI) | 0·88 (0·86–0·89) | | 0·88 (0·86–0·89) | |

SHR; sub-distribution hazard ratios; *the SHR are from the Fine and Gray's proportional sub-hazards model, AUC; area under receiver operating characteristics. Meningitis: positive CSF culture, or positive CSF microscopy, or positive CSF antigen test, or elevated CSF WBC count (≥20 in young infants aged 0-28 days OR, ≥10 in young infants aged 29-59 days) PLUS a positive blood culture for a known pathogen

For peer review only

Table S7. **Estimated causes of post-discharge deaths during readmission at KCH (67 deaths)**.

| **Index admission diagnosis (N=67)** | **No. (%)** | **Causes of post-discharge deaths (N=67)** | **No. (%)** |
|---|---|---|---|
| Neonatal sepsis | 15 (22) | Neonatal sepsis | 16 (24) |
| Preterm complications | 15 (22) | Preterm complications | 15 (22) |
| Heart disease-Congenital | 9 (13) | Heart disease-Congenital | 10 (15) |
| Neonatal jaundice | 5 (7.5) | Neonatal jaundice | 5 (7.4) |
| Meningitis | 4 (6.0) | Meningitis | 5 (7.4) |
| Birth asphyxia | 4 (6.0) | Birth asphyxia | 5 (7.4) |
| Lower respiratory tract infection | 4 (6.0) | Lower respiratory tract infection | 4 (6.0) |
| Encephalopathy - unknown | 0 | Encephalopathy - unknown | 1 (1.5) |
| Hydrocephalus | 1 (1.5) | Hydrocephalus | 1 (1.5) |
| Malnutrition | 1 (1.5) | None specified | 5 (7.4) |
| None specified | 9 (13) | | |

Index admission diagnosis and causes of death were assigned by treating clinician.



For peer review only - http://bmjopen.bmj.com/site/about/guidelines.xhtml

Table S8. **Univariate analysis of admission features associated with post-discharge deaths.**

| N=3625 | Deaths (N=208) | Crude HR | P-value | Scaled Schoenfeld residuals P-value |
|---|---|---|---|---|
| Demographics | | | | |
| Age in days | | | | |
| 0 to 2 | 124 (5.6) | 0.98 (0.67–1.44) | 0.92 | 0.10 |
| 3 to 7 | 15 (3.7) | 0.63 (0.34–1.16) | 0.14 | |
| 8 to 28 | 36 (6.6) | 1.17 (0.73–1.87) | 0.52 | |
| >28 | 33 (5.5) | Reference | | |
| Sex (female) | 89 (5.5) | 0.98 (0.74–1.28) | 0.86 | 0.79 |
| Reported born premature | 58 (8.0) | 1.79 (1.32–2.44) | <0.001 | 0.07 |
| Reported low birth weight | 33 (9.3) | 1.99 (1.37–2.90) | <0.001 | 0.13 |
| Born at KCH | | | | |
| Yes | 102 (4.3) | Reference | | |
| No | 106 (7.6) | 1.75 (1.34–2.30) | <0.001 | 0.12 |
| Length of hospitalization (days)-log transformed | - | 1.96 (1.68–2.27) | <0.001 | 0.38 |
| Discharged over weekend | | | | |
| No | 173 (5.7) | Reference | | |
| Yes | 35 (4.8) | 0.85 (0.59–1.23) | 0.39 | 0.16 |
| Type of discharge | | | | |
| Normal discharge | 180 (4.9) | Reference | | 0.75 |
| Absconded | 5 (12) | 2.60 (1.07–6.33) | 0.04 | |
| Transferred/referred | 23 (44) | 11.8 (7.64–18.2) | <0.001 | |
| **Anthropometry** | | | | |
| Weight (kg) | | | | |
| <1.5 | 30 (8.5) | 2.49 (1.65–3.77) | <0.001 | 0.17 |
| 1.5 to <2.5 | 87 (9.2) | 2.64 (1.97–3.54) | <0.001 | |
| ≥2.5 | 91 (3.7) | Reference | | |
| Missing weight | 0 | - | | |
| MUAC (cm) | | | | |
| <9.0 | 88 (8.8) | 4.05 (2.56–6.41) | <0.001 | 0.17 |
| 9 to 10 | 44 (5.8) | 2.56 (1.55–4.24) | <0.001 | |
| 10 to 11 | 42 (4.5) | 1.92 (1.15–3.18) | 0.01 | |
| ≥11 | 23 (2.4) | Reference | | |
| Missing MUAC | 11 (12) | 5.83 (2.84–12.0) | <0.001 | |
| **Clinical features** | | | | |
| Axillary temperature | | | | |
| <36˚C | 78 (8.1) | 1.45 (1.07–1.96) | 0.02 | 0.80 |
| 36 to 37.5˚C | 88 (5.7) | Reference | | |
| >37.5˚C | 41 (3.3) | 0.57 (0.40–0.83) | 0.003 | |
| Missing temperature | 1 (5.0) | 0.99 (0.14–7.12) | 0.99 | |
| Respiratory rate/min | | | | |
| Bradypnoea | 7 (8.8) | 1.92 (0.90–4.13) | 0.09 | 0.30 |
| Normal | 108 (4.7) | Reference | | |
| Tachypnoea | 87 (6.8) | 1.44 (1.09–1.92) | 0.01 | |
| Missing | 6 (7.2) | 1.65 (0.72–3.76) | 0.23 | |
| Heart rate/min | | | | |



For peer review only - http://bmjopen.bmj.com/site/about/guidelines.xhtml

| | | | | |
|---|---|---|---|---|
| Bradycardia | 9 (11) | 2.10 (1.07–4.13) | 0.03 | 0.73 |
| Normal | 137 (5.5) | Reference | | |
| Tachycardia | 62 (5.3) | 0.97 (0.72–1.31) | 0.85 | |
| Missing | 0 | - | | |
| Hypoxia (SaO2 <90%) | 51 (8.2) | 1.68 (1.23–2.31) | 0.001 | 0.54 |
| Lower chest wall indrawing | 108 (6.8) | 1.54 (1.17–2.02) | 0.002 | 0.18 |
| Wheeze | 2 (4.4) | 0.77 (0.19–3.10) | 0.71 | 0.20 |
| Stridor | 0 | - | | |
| Breathing difficulty | 109 (6.5) | 1.40 (1.07–1.85) | 0.02 | 0.26 |
| Cyanosis | 7 (6.3) | 1.14 (0.54–2.43) | 0.73 | 0.17 |
| Capillary refill ≥2 seconds | 4 (9.4) | 1.81 (0.67–4.87) | 0.24 | 0.40 |
| Temperature gradient | 12 (7.5) | 1.44 (0.80–2.57) | 0.23 | 0.22 |
| Weak pulse | 7 (15) | 3.10 (1.46–6.59) | 0.003 | 0.38 |
| Lethargy | 14 (5.8) | 1.06 (0.63–1.80) | 0.82 | 0.19 |
| Impaired consciousness | 6 (5.5) | 0.98 (0.44–2.21) | 0.96 | 0.50 |
| Bulging fontanel | 4 (15) | 3.04 (1.13–8.18) | 0.03 | 0.06 |
| Stiff neck | 1 (17) | 2.84 (0.40–20.2) | 0.30 | 0.15 |
| Convulsions | 9 (5.0) | 0.88 (0.46–1.75) | 0.75 | 0.31 |
| Sunken eyes | 6 (15) | 3.31 (1.47–7.45) | 0.004 | 0.20 |
| Reduced skin turgor | 8 (11) | 2.19 (1.08–4.43) | 0.03 | 0.20 |
| Pallor | 15 (10) | 2.09 (1.23–3.53) | 0.006 | 0.23 |
| **Laboratory features** | | | | |
| Meningitis | 4 (16) | 3.98 (1.45–10.9) | 0.007 | 0.13 |
| Anaemia (haemoglobin <11 g/dl) | 26 (6.2) | 1.19 (0.79–1.80) | 0.41 | 0.68 |
| HIV antibody positive | 7 (6.2) | 1.17 (0.55–2.49) | 0.69 | 0.76 |
| Malaria slide positive | 0 | - | | |
| Bacteraemia | 10 (9.4) | 1.02 (0.50–2.06) | 0.96 | 0.28 |
| Blood glucose (mmols/L) | | | | |
| <2.6 | 51 (6.9) | 1.31 (0.92–1.85) | 013 | |
| 2.6 to 7.0 | 86 (5.3) | Reference | | 0.25 |
| >7.0 | 10 (6.3) | 1.21 (0.63–2.32) | 0.57 | |
| Missing blood glucose | 61 (5.0) | 0.95 (0.68–1.32) | 0.75 | |
| White blood cells ($10^{12}$ cells/L) | | | | |
| <4 | 1 (2.5) | 0.45 (0.06–3.20) | 0.42 | |
| 4-20 | 157 (5.5) | Reference | | 0.79 |
| >20 | 29 (6.1) | 1.10 (0.74–1.64) | 0.63 | |
| Missing | 21 (5.4) | 0.99 (0.63–1.56) | 0.97 | |
| Platelets ($10^9$ cells/L) | | | | |
| <150 | 38 (8.6) | 1.69 (1.18–2.41) | 0.004 | |
| ≥150 | 149 (5.1) | Reference | | 0.70 |
| Missing | 21 (5.4) | 1.08 (0.68–1.70) | 0.75 | |

HR: hazard ratios; the HR are from the Cox proportional hazards model.



For peer review only - http://bmjopen.bmj.com/site/about/guidelines.xhtml

Table S9. **Univariate regression analysis of factors associated with inpatient and post-discharge mortality among children born at KCH only.**

| **N=1,358** | **Deaths (N=174) N (%)** | **Crude SHR** | **P-value** | **Scaled Schoenfeld residuals P-value** |
|---|---|---|---|---|
| Demographics | | | | |
| Age in days | | | | |
| 0 to 2 | 151 (15) | 2.48 (1.19–5.16) | 0.02 | 0.26 |
| 3 to 7 | 8 (7.0) | 1.22 (0.45–3.30) | 0.69 | |
| 8 to 28 | 8 (6.8) | 1.21 (0.46–3.23) | 0.70 | |
| >28 | 7 (5.5) | Reference | | |
| Sex (female) | 84 (14) | 1.11 (0.83–1.48) | 0.50 | 0.31 |
| Born premature | 101 (21) | 2.65 (1.97–3.56) | <0.001 | 0.10 |
| Born low birth weight | 107 (21) | 2.74 (2.03–3.71) | <0.001 | 0.44 |
| **Anthropometry** | | | | |
| Weight z score at birth | | | | |
| <-2.0 | 32 (17) | 1.49 (1.02–2.17) | 0.04 | 0.12 |
| ≥-2.0 | 142 (12) | Reference | | |
| MUAC (cm) | | | | |
| <9.0 | 97 (23) | 3.14 (2.05–4.83) | <0.001 | 0.17 |
| 9 to 10 | 26 (9.9) | 1.26 (0.74–2.17) | 0.40 | |
| 10 to 11 | 25 (7.3) | 0.91 (0.53–1.57) | 0.73 | |
| ≥11 | 26 (7.9) | Reference | | |
| Missing MUAC | 0 | - | | |
| **Clinical features** | | | | |
| Axillary temperature | | | | |
| <36˚C | 113 (26) | 3.02 (2.13–4.27) | <0.001 | 0.30 |
| 36 to 37.5˚C | 41 (9.2) | Reference | | |
| >37.5˚C | 18 (3.9) | 0.42 (0.24–0.72) | 0.002 | |
| Missing temperature | 2 (50) | 6.90 (1.84–25.8) | 0.004 | |
| Respiratory rate/min | | | | |
| Bradypnoea | 32 (52) | 6.44 (4.39–9.45) | <0.001 | 0.17 |
| Normal | 91 (10) | Reference | | |
| Tachypnoea | 50 (12) | 1.21 (0.86–1.69) | 0.28 | |
| Missing | 1 (5.3) | 0.49 (0.07–3.37) | 0.47 | |
| Heart rate/min | | | | |
| Bradycardia | 21 (43) | 4.76 (3.06–7.43) | <0.001 | 0.81 |
| Normal | 98 (10) | Reference | | |
| Tachycardia | 55 (15) | 1.48 (1.07–2.05) | 0.02 | |



For peer review only - http://bmjopen.bmj.com/site/about/guidelines.xhtml

For peer review only

| | | | | |
|---|---|---|---|---|
| Missing | 0 | - | | |
| Hypoxia (SaO2 <90%) | 79 (29) | 3.62 (2.71–4.83) | <0.001 | 0.81 |
| Lower chest wall indrawing | 125 (21) | 3.57 (2.57–4.95) | <0.001 | 0.47 |
| Wheeze | 0 | - | | |
| Stridor | 5 (63) | 6.96 (3.09–15.7) | <0.001 | 0.60 |
| Breathing difficulty | 481 (22) | 5.40 (3.69–7.88) | <0.001 | 0.18 |
| Cyanosis | 29 (48) | 5.15 (3.54–7.50) | <0.001 | 0.18 |
| Capillary refill >2 seconds | 14 (64) | 7.22 (4.43–11.7) | <0.001 | 0.58 |
| Temperature gradient | 26 (30) | 2.69 (1.81–4.01) | <0.001 | 0.49 |
| Weak pulse | 25 (58) | 6.52 (4.44–9.56) | <0.001 | 0.77 |
| Lethargy | 17 (16) | 1.29 (0.79–2.10) | 0.31 | 0.56 |
| Impaired consciousness | 48 (52) | 6.75 (4.93–9.24) | <0.001 | 0.13 |
| Bulging fontanel | 1 (25) | 2.03 (0.31–13.2) | 0.46 | 0.59 |
| Stiff neck | 1 (5.3) | - | | 0.31 |
| Convulsions | 1 (2.9) | 0.21 (0.03–1.48) | 0.12 | 0.21 |
| Sunken eyes | 1 (17) | 1.35 (0.19–9.77) | 0.77 | 0.57 |
| Reduced skin turgor | 1 (5.9) | 0.44 (0.06–3.19) | 0.42 | 0.56 |
| Pallor | 21 (42) | 4.00 (2.61–6.12) | <0.001 | 0.59 |
| **Laboratory features** | | | | |
| Meningitis | 2 (29) | 9.95 (2.37–41.8) | 0.002 | 0.12 |
| Haemoglobin <11 g/dl | 11 (13) | 0.92 (0.51–1.66) | 0.78 | 0.25 |
| HIV antibody positive | 8 (21) | 1.68 (0.84–3.33) | 0.14 | 0.14 |
| Malaria slide positive | 0 | - | | |
| Bacteraemia | 14 (33) | 3.00 (1.48–5.95) | 0.002 | 0.20 |
| Blood glucose (mmols/l) | | | | |
| <2.6 | 33 (12) | 1.02 (0.67–1.56) | 0.91 | |
| 2.6 to 7.0 | 58 (12) | Reference | | 0.95 |
| >7.0 | 11 (21) | 1.88 (1.00–3.54) | 0.05 | |
| Missing blood glucose | 72 (13) | 1.11 (0.79–1.56) | 0.53 | |
| White blood cells ($10^{12}$ cells/L) | | | | |
| <4 | 3 (33) | 3.52 (1.25–9.91) | 0.02 | |
| 4-20 | 100 (10) | Reference | | 0.08 |
| >20 | 59 (28) | 3.10 (2.27–4.24) | <0.001 | |
| Missing | 12 (8.2) | 0.82 (0.45–1.48) | 0.50 | |
| Platelets ($10^9$ cells/L) | | | | |
| <150 | 35 (20) | 1.69 (1.18–2.44) | 0.005 | |
| ≥150 | 127 (12) | Reference | | 0.50 |
| Missing | 12 (8.2) | 0.66 (0.37–1.19) | 0.17 | |

SHR; sub-distribution hazard ratios; the SHR are from the Fine and Gray's proportional sub-hazards model.



For peer review only - http://bmjopen.bmj.com/site/about/guidelines.xhtml

Table S10. **Multivariable regression analysis of factors associated with inpatient and post-discharge mortality among children born at KCH only.**

| | Inpatient analysis | | Post-discharge analysis | |
|---|---|---|---|---|
| | Adjusted SHR* | P-value | Adjusted HR | P-value |
| **Demographics** | | | | |
| Age in days | | | | |
| 0 to 2 | 3.03 (1.33–6.94) | 0.009 | ¶ | |
| 3 to 7 | 4.08 (1.48–11.3) | 0.007 | ¶ | |
| 8 to 28 | 2.57 (0.90–7.29) | 0.08 | ¶ | |
| >28 | Reference | | | |
| **Anthropometry** | | | | |
| Low birth weight | 1.55 (1.10–2.20) | 0.01 | 2.76 (1.30–5.82) | 0.008 |
| **Clinical features** | | | | |
| Axillary temperature | | | | |
| <36˚C | 1.49 (0.97–2.28) | 0.07 | 1.44 (0.71–2.95) | 0.31 |
| 36 to 37.5˚C | Reference | | Reference | |
| >37.5˚C | 0.67 (0.39–1.13) | 0.13 | 0.22 (0.06–0.78) | 0.02 |
| Missing temperature | 3.37 (0.85–13.4) | 0.09 | 0.48 (0.09–2.64) | 0.40 |
| Respiratory rate/min | | | | |
| Bradypnoea | 2.22 (1.36–3.63) | 0.001 | ¶ | |
| Normal | Reference | | | |
| Tachypnoea | 0.78 (0.55–1.11) | 0.17 | ¶ | |
| Missing | - | | | |
| Heart rate/min | | | | |
| Bradycardia | 1.88 (1.14–3.12) | 0.01 | 3.56 (1.14–11.2) | 0.03 |
| Normal | Reference | | Reference | |
| Tachycardia | 1.57 (1.11–2.21) | 0.01 | 1.54 (0.77–3.07) | 0.22 |
| Missing | 0.25 (0.02–3.00) | 0.28 | - | |
| Hypoxia (SaO2 <90%) | 1.60 (1.14–2.24) | 0.006 | ¶ | |
| Lower chest wall indrawing | 1.42 (0.91–2.22) | 0.12 | ¶ | |
| Stridor | 3.74 (1.87–7.49) | <0.001 | ¶ | |
| Breathing difficulty | 2.13 (1.25–3.64) | 0.005 | ¶ | |
| Capillary refill >2 seconds | 1.94 (1.06–3.56) | 0.03 | ¶ | |
| Weak pulse | 2.15 (1.27–3.65) | 0.004 | 1.60 (0.24–10.5) | 0.63 |
| Pallor | 2.36 (1.46–3.83) | <0.001 | ¶ | |
| **Laboratory features** | | | | |
| Bacteraemia | 2.50 (1.20–5.22) | 0.02 | 0.21 (0.03–1.81) | 0.16 |
| Model performance | | | | |
| AUC (95% CI) | 0.85 (0.82–0.88) | | 0.79 (0.72–0.85) | |

SHR; sub-distribution hazard ratios; *the SHR are from the Fine and Gray's proportional subhazards model, HR-Hazard ratio from the Proportional Cox regression model, ¶; variables not selected for inclusion in the multivariable model, AUC; area under receiver operating characteristics.

For peer review only



For peer review only - http://bmjopen.bmj.com/site/about/guidelines.xhtml

Figure S1. **A: Monthly all young infant admissions, B: all admissions (<60 months old), C: all admissions (<60 months old) case fatality and D: 2–59 months old case fatality before and after July 2013.**

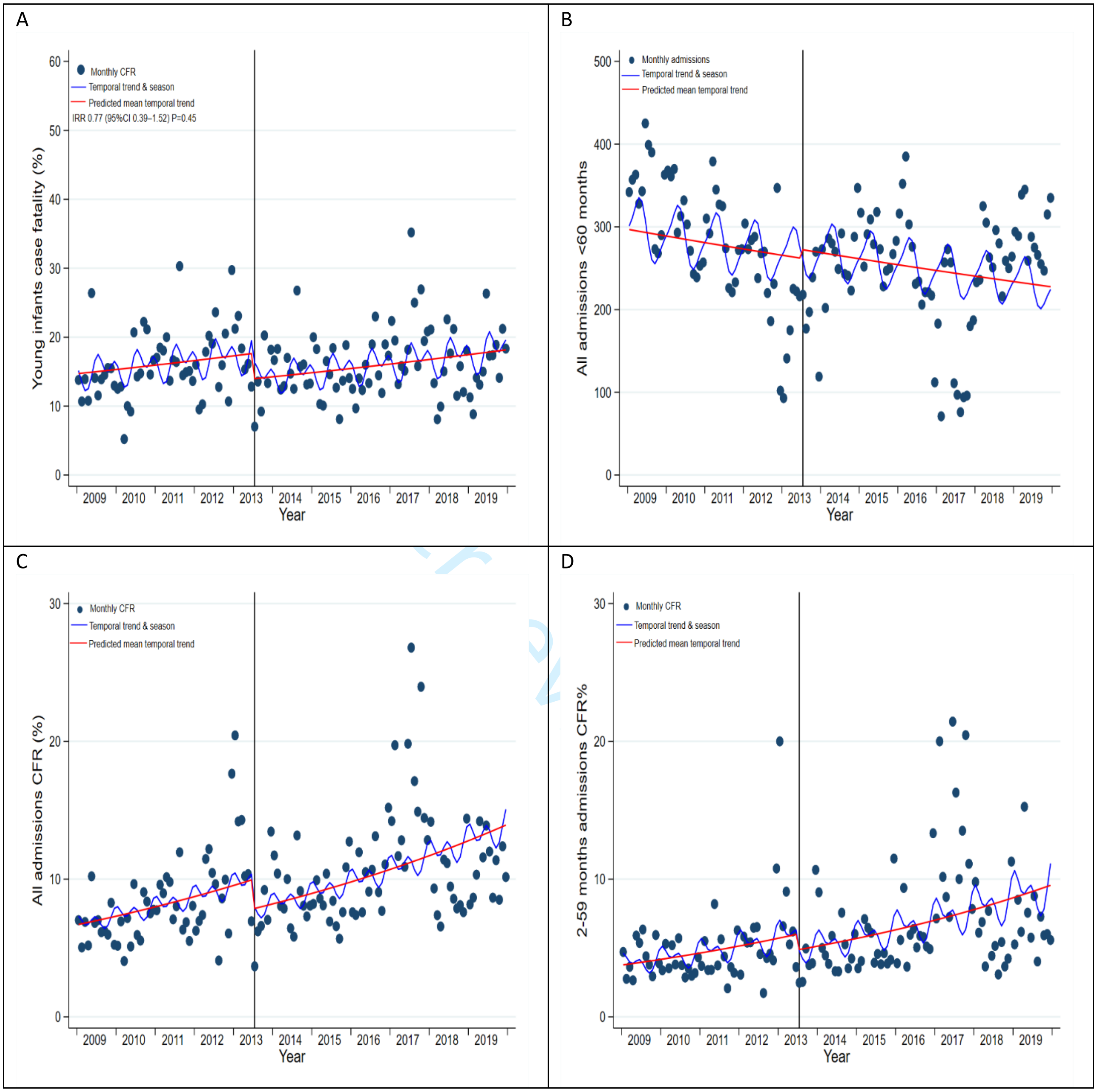

Supplement: Author's manuscript [file bmjopen-2022-067482.draft_revisions.pdf]
